# Non-Isothermal Crystallization Behavior and Crystal Morphology of EPDM/PP Thermoplastic Vulcanizates Containing TAC and OV-POSS Coagents

**DOI:** 10.3390/polym18212675

**Published:** 2026-10-28

**Authors:** Nazlı Yazıcı Çakır, Özgenur İnan Coşkun, Merve Ergün, Mehmet Kodal, Güralp Özkoç

**Affiliations:** 1 Department of Chemical Engineering, Kocaeli University, 41001 Kocaeli, Türkiye; mervemetin1576@gmail.com (M.E.); mehmet.kodal@kocaeli.edu.tr (M.K.); 2 Department of Machinery and Metal Technologies, Hereke Asım Kocabıyık Vocational School, Kocaeli University, 41800 Kocaeli, Türkiye; 3 Polymer Science and Technology Graduate Program, Kocaeli University, 41001 Kocaeli, Türkiye; inannozgee@gmail.com; 4 Department of Chemistry, Istinye University, 34396 Istanbul, Türkiye; guralp.ozkoc@istinye.edu.tr; 5 Xplore Instruments B.V., 6135 KT Sittard, The Netherlands

**Keywords:** EPDM/PP, thermoplastic vulcanizates, coagents, OV-POSS, non-isothermal crystallization kinetics, dynamic vulcanization

## Abstract

Ethylene propylene diene monomer rubber/polypropylene (EPDM/PP) thermoplastic vulcanizates containing triallyl cyanurate (TAC) or octavinyl polyhedral oligomeric silsesquioxane (OV-POSS) coagents were prepared by dynamic vulcanization. The effects of coagent type, loading level, and EPDM/PP ratio on non-isothermal crystallization were investigated using differential scanning calorimetry (DSC), polarized optical microscopy (POM), and X-ray diffraction (XRD). Crystallization kinetics were evaluated using the Avrami, Ozawa, and combined Avrami-Ozawa models, while nucleation activity and apparent activation energies were assessed using the Dobreva-Gutzow, Kissinger, Takhor, and Friedman approaches. Both coagents, particularly OV-POSS, shifted crystallization onset toward higher temperatures, demonstrating enhanced heterogeneous nucleation. The decreasing Avrami-derived half-times *t_0.5_* with increasing cooling rate were qualitatively consistent with the experimental DSC half-times *t_1/2_*. The Ozawa model did not adequately describe the investigated systems, while the combined Avrami-Ozawa model provided satisfactory correlations. The influence of the coagents on the overall crystallization rate depended on composition, loading, and cooling rate. In particular, high OV-POSS loading promoted earlier nucleation but did not consistently shorten *t_0.5_*, especially in the EPDM-rich TPVs. POM revealed earlier crystal formation, increased nucleation density, and smaller spherulites in the coagent-containing samples. DSC analysis revealed an increase in PP-normalized crystallinity upon OV-POSS incorporation, while XRD confirmed an increased crystalline fraction, predominantly associated with the α-crystalline form. This effect was particularly pronounced in the PP-rich formulations. Although OV-POSS promoted heterogeneous nucleation and crystalline structure development, its influence on the overall crystallization rate remained dependent on blend composition, coagent loading, and cooling conditions, suggesting a complex interplay between nucleation and subsequent crystal growth.

## 1. Introduction

Thermoplastic vulcanizates (TPVs) are multiphase materials designed to combine the elastic response of crosslinked rubbers with the melt-processability of thermoplastics. This combination is achieved by dynamic vulcanization, a reactive melt-mixing process in which the elastomer phase is selectively crosslinked while being fragmented and dispersed within a molten thermoplastic phase. The resulting morphology generally consists of crosslinked elastomeric domains embedded in a continuous or semi-continuous thermoplastic matrix, thereby enabling the material to retain rubber-like behavior while remaining reprocessable using conventional thermoplastic-processing techniques [1,2,3].

Among the different TPV combinations, ethylene propylene diene monomer rubber/polypropylene (EPDM/PP) blends are particularly important because of their favorable balance of elasticity, low density, environmental resistance, thermal stability, and processability. The final properties of EPDM/PP TPVs are strongly governed by the morphology developed during dynamic vulcanization. Rubber-domain size and distribution, phase continuity, interfacial adhesion, viscosity ratio, crosslink density, and mixing history collectively determine the mechanical and rheological behavior of these materials [2,4,5]. Recent studies have also shown that recycling history, thermal ageing, and the molecular characteristics of EPDM can alter the morphology and macroscopic performance of EPDM/PP TPVs [6,7,8,9].

The thermoplastic PP phase remains molten during dynamic vulcanization and subsequently solidifies through crystallization during cooling. Its crystallization behavior is therefore a critical but sometimes underemphasized component of TPV structure development. The onset and rate of crystallization influence processing cycle time and solidification, whereas crystal morphology and the final degree of crystallinity affect stiffness, dimensional stability, thermal resistance, and mechanical performance. In dynamically vulcanized blends, PP crystallizes in a heterogeneous environment containing crosslinked rubber particles, interfacial regions, residual stresses, and reactive additives. Consequently, the crystallization of PP is closely coupled with the morphology created during dynamic vulcanization rather than being an independent property of the thermoplastic component.

The dispersed elastomeric phase can exert competing effects on PP crystallization. Rubber particles and polymer–rubber interfaces may provide heterogeneous nucleation sites, promoting crystallization at higher temperatures. Conversely, the presence of crosslinked elastomer domains can restrict PP-chain diffusion and reduce the space available for lamellar organization and crystal growth. The balance between these mechanisms depends on the EPDM/PP ratio, rubber-domain dimensions, interfacial interactions, crosslinking level, and cooling conditions. Cao et al. [10] demonstrated that dynamic vulcanization and in situ compatibilization significantly altered both the phase morphology and non-isothermal crystallization behavior of PP/EPDM blends. Song et al. [9] similarly reported that differences in the molecular characteristics of EPDM affected the crystallization behavior and structure of EPDM/PP TPVs. These observations indicate that an increase in crystallization onset temperature should not automatically be interpreted as an increase in the final degree of crystallinity. In a complementary study on β-nucleated PP, Krajenta and Pawlak [11] showed that reducing macromolecular entanglements affected crystallization behavior, highlighting the sensitivity of crystal development to molecular constraints. More recently, Wang et al. [12] investigated the non-isothermal crystallization behavior of concentrated EPDM/PP TPVs and discussed the competing effects of the dispersed rubber phase on heterogeneous nucleation and chain mobility. Their findings further emphasize the importance of distinguishing crystallization onset from the subsequent development of crystallinity.

Peroxide curing is frequently employed in EPDM/PP TPV production to crosslink the elastomer phase. However, peroxide-initiated reactions may also cause PP degradation through β-chain scission and involve competing termination reactions, including disproportionation. Coagents are therefore incorporated to improve crosslinking efficiency and mitigate undesirable side reactions [13]. Multifunctional auxiliary crosslinking agents, commonly referred to as coagents, can be incorporated to improve peroxide utilization, increase crosslinking efficiency, and suppress undesirable side reactions. Triallyl cyanurate (TAC) is a conventional organic coagent containing three reactive allyl groups, whereas octavinyl polyhedral oligomeric silsesquioxane (OV-POSS) contains eight vinyl functionalities attached to a rigid organic–inorganic silsesquioxane cage.

POSS compounds are molecularly defined hybrid structures composed of a nanoscale Si-O cage surrounded by chemically tailorable organic groups [14]. Depending on their functionality and compatibility with the surrounding polymers, POSS derivatives can modify interfacial interactions and phase morphology. For example, Tian et al. [15] demonstrated that functionalized POSS nanoparticles improved compatibility and refined the dispersed phase in TPU/MVQ blends, highlighting the importance of POSS surface chemistry in multiphase polymer systems. Consequently, POSS structures should not always be regarded as conventional inert nanofillers. Their effects are determined by their molecular architecture, functional groups, dispersion state, loading level, and ability to interact or react with the surrounding polymer chains.

The incorporation of POSS into semicrystalline polymers may affect crystal nucleation and crystal growth in different ways. Chen et al. [16] showed that relatively low POSS concentrations could modify the isothermal crystallization behavior of isotactic PP, while Fina et al. [17] reported that the chemical structure and compatibility of POSS affected the morphology and crystalline behavior of PP/POSS nanocomposites. More recent studies have demonstrated that appropriately designed POSS-supported nucleating structures can accelerate PP nucleation and modify its crystallization and rheological behavior [18,19]. Silsesquioxane-containing modifiers have also been shown to influence the crystallization and physical properties of nucleated PP films [20]. However, POSS does not universally increase crystallinity. Strong polymer-POSS interactions, increased network density, aggregation, or restricted molecular mobility may facilitate the formation of crystal nuclei while simultaneously limiting subsequent lamellar growth.

In TPV systems, the effects of POSS are further complicated by the dynamically generated phase morphology. Xu et al. [21] demonstrated that POSS modification influenced the thermal and combustion behavior of TPV composites, indicating that silsesquioxane structures can modify the thermal response of dynamically vulcanized systems. Nevertheless, these improvements should not be generalized to all POSS types or TPV formulations because the response depends on POSS functionality, matrix composition, interfacial interactions, and dispersion. Studies involving other modifiers, such as magnesium acrylate and cellulose nanocrystals, have similarly demonstrated that interfacial compatibilization and morphology refinement can substantially alter the properties of EPDM/PP TPVs [5,22]. More recently, Xu and Zong [23] investigated reactive interfacial compatibilization in sea-island-structured EPDM/PP TPVs using magnesium maleic and maleic anhydride and reported improvements in mechanical and shape-memory performance. This work further illustrates the importance of interfacial modification in determining the response of EPDM/PP TPVs. These findings reinforce the importance of distinguishing the direct nucleating effect of a modifier from its indirect influence through changes in phase morphology.

In our previous study [13], OV-POSS was investigated as a reactive coagent for the peroxide-initiated dynamic vulcanization of EPDM/PP blends and compared with conventional TAC. That study focused on establishing the feasibility of OV-POSS-assisted TPV production and evaluating the resulting network formation, phase morphology, and mechanical and rheological properties. SEM and AFM analyses revealed changes in phase organization and refinement of the dispersed EPDM domains, while swelling measurements provided information on crosslink density. Together, these results established relationships between coagent selection, the dynamically generated phase structure, and the bulk properties of the TPVs.

The present study addresses a different question: how these coagent-containing multiphase systems crystallize during cooling and how coagent selection affects the resulting crystalline morphology and structure. Although similar formulation families are investigated, the previous work did not provide a systematic analysis of non-isothermal crystallization kinetics or the development of crystalline morphology across different cooling conditions. Here, the comparison of TAC and OV-POSS is extended to crystallization behavior in both PP-rich and EPDM-rich compositions using DSC, kinetic analyses, POM, and XRD. The specific research gap therefore concerns the relationship between reactive coagent selection, blend composition, and crystalline-phase development, rather than the feasibility of TPV preparation or its mechanical and rheological performance. The present work builds on the previously established network and phase-morphology results while providing a distinct investigation of crystallization kinetics and crystal structure. This unresolved issue is particularly relevant because OV-POSS may affect crystallization through several simultaneous mechanisms. Its cage-like structure and the refined EPDM phase morphology may increase the number of heterogeneous nucleation sites and initiate PP crystallization at higher temperatures. At the same time, the participation of its eight vinyl groups in peroxide-induced reactions may increase network density, restrict polymer-chain mobility, and suppress subsequent crystal growth. Therefore, enhanced heterogeneous nucleation does not necessarily lead to faster overall crystallization, as increased network density and restricted chain mobility may influence subsequent crystal growth. The resulting degree of crystallinity depends on the balance between these competing effects, which may vary with blend composition and coagent loading. To the best of our knowledge, this competition between nucleation enhancement and growth restriction has not previously been systematically examined for OV-POSS used as a reactive coagent in EPDM/PP TPVs. Moreover, direct comparisons between OV-POSS and a conventional coagent such as TAC across both PP-rich and EPDM-rich formulations remain limited.

The crystallization kinetics were evaluated using the Avrami, Ozawa, and combined Avrami–Ozawa models in this study. Apparent crystallization activation energies were determined using the Kissinger, Takhor, and Friedman approaches, while heterogeneous nucleation activity was assessed using the Dobreva-Gutzow method. Polarized optical microscopy was employed to monitor crystallization onset, nucleation density, and spherulitic development, and X-ray diffraction was used to characterize the resulting crystalline structure. Through this integrated approach, the study aims to distinguish the nucleation-promoting effect of OV-POSS from possible contributions of network-related constraints to subsequent crystal development. The findings are expected to provide a mechanistic basis for controlling the solidification behavior and crystalline morphology of dynamically vulcanized EPDM/PP systems through coagent selection and blend composition.

## 2. Materials and Methods

### 2.1. Materials

Polypropylene (PP; Moplen HP456J, LyondellBasell, Belgium) and ethylene propylene diene monomer rubber (EPDM; Dutral TER 4548, Versalis, Eschborn, Germany) were used as the thermoplastic and elastomeric phases, respectively. The EPDM grade contained 50 wt.% paraffinic oil and had an ENB content of 4.5 wt.%. The reported EPDM/PP ratios were calculated on a dry-polymer mass basis, excluding the paraffinic oil contained in the commercial EPDM. Dynamic vulcanization was carried out using dicumyl peroxide (DCP, Luperox DC40P, Arkema, Colombes, France) as the peroxide curing agent. Triallyl cyanurate (TAC/GR 50, 50 wt.% active TAC in granular form, Kettlitz, Rennertshofen, Germany) was used as the conventional coagent. Octavinyl polyhedral oligomeric silsesquioxane (OV-POSS, Hybrid Plastics Inc., Hattiesburg, MS, USA) was utilized as the reactive coagent. A combination of Irganox^®^ and Irgafos^®^ antioxidants (BASF, Ludwigshafen, Germany) was incorporated to minimize thermo-oxidative degradation during processing.

Detailed material specifications and physicochemical characteristics of all constituents were reported in our previous study [13].

### 2.2. Processing

The EPDM/PP-based thermoplastic vulcanizates (TPVs) were prepared through peroxide-initiated dynamic vulcanization using the procedure established in our previous study [13]. Melt compounding was carried out in a laboratory-scale co-rotating twin-screw microcompounder (MC 15 HT, Xplore Instruments, Sittard, The Netherlands) equipped with a recirculation channel. The barrel temperature was maintained at 175 °C and the screw speed was 100 rpm throughout the process. The total mixing time was 5 min. Initially, PP, EPDM, the selected coagent (TAC/GR 50 or OV-POSS), and antioxidants were compounded under melt-mixing conditions. Dicumyl peroxide was added after 3 min of mixing, and mixing was continued for a further 2 min, giving a total mixing time of 5 min. During this stage, crosslinking of EPDM and breakup of the rubber phase occurred simultaneously, leading to the development of the characteristic TPV morphology. At the end of the compounding step, the materials were transferred into a micro-injection molding machine (12 mL Xplore micro-injection molding machine, Sittard, The Netherlands) and molded into standard specimens. The melt and mold temperatures were 175 °C and 25 °C, respectively, while both injection and holding pressures were maintained at 8 bar.

In the present study, the coagent/DCP ratio was taken as 3 and 7 and only the TPV formulations containing TAC and OV-POSS and possessing EPDM/PP weight ratios of 70/30 and 30/70 were selected for crystallization investigations. These compositions were chosen because they represent elastomer-rich and thermoplastic-rich TPV morphologies, respectively, allowing the influence of phase composition on crystallization kinetics to be evaluated systematically.

The samples were designated according to the EPDM/PP composition, coagent type, and coagent-to-DCP mass ratio, as illustrated by 70/30 EPDM/PP/Coagent(X). Here, X denotes the mass ratio of the commercial coagent product to the commercial DCP product added (Table 1). Thus, X = 3 and X = 7 indicate coagent masses equal to three and seven times the DCP product mass, respectively. The coagent amounts (phr) were calculated by multiplying the DCP content (phr) by the corresponding coagent-to-DCP mass ratio (X). Accordingly, the coagent loadings were 5.25 and 12.25 phr for the 70/30 formulations and 2.25 and 5.25 phr for the 30/70 formulations at X = 3 and X = 7, respectively. For TAC-containing formulations, these amounts refer to the commercial TAC/GR 50 product, which contains 50 wt.% active TAC, rather than to the active TAC mass alone. Therefore, TAC and OV-POSS were compared at equal commercial-product coagent-to-DCP mass ratios rather than equivalent reactive-group concentrations. Differences in functionality, molar mass, and active content should be considered when interpreting their relative effects.

### 2.3. Characterization

Since the materials, formulations, and dynamic vulcanization procedure employed in this study were identical to those reported in our previous work [13], only the characterization techniques used to investigate the crystallization behavior of the TPV samples are described herein.

The non-isothermal crystallization behavior of the TPV samples was investigated using a Mettler Toledo DSC 1 Star System differential scanning calorimeter under a nitrogen atmosphere. Samples weighing approximately 20 mg were used. Three replicate DSC measurements were performed for each formulation at each cooling rate, and the reported crystallization temperatures represent the mean values of these measurements. The samples were first heated from room temperature to 220 °C at a heating rate of 10 °C/min and maintained at this temperature for 5 min to eliminate their previous thermal history. Subsequently, the samples were cooled to room temperature in separate runs at cooling rates of 5, 10, 20, and 30 °C/min. For crystallinity determination, the samples cooled at 10 °C/min were subsequently reheated to 220 °C at a heating rate of 10 °C/min. The onset crystallization temperature (T_c,o_), peak crystallization temperature (T_c,p_), and end crystallization temperature (T_c,e_) were determined from the exothermic crystallization curves. The crystallization interval (ΔT), calculated as the difference between T_c,o_ and T_c,e_, was used to evaluate the overall crystallization range of the samples. In addition, the relative crystallinity as a function of crystallization time was obtained from the DSC exotherms, and the crystallization half-time (*t_1/2_*), corresponding to the time required to achieve 50% of the total crystallinity, was determined to compare the crystallization rates of the TPV formulations. The degree of crystallinity of the PP phase (X_c,DSC_) was calculated from the melting enthalpy (ΔH_m_) determined by integrating the endothermic peaks recorded during the second heating scan at 10 °C/min. The calculations were normalized to the PP mass fraction in the total formulation, taking into account the paraffinic oil contained in the commercial EPDM and the other formulation components.

The crystallization kinetics were further analyzed using the Avrami, Ozawa, and combined Avrami-Ozawa (Mo) models. Furthermore, the activation energy of crystallization was estimated using the Kissinger, Takhor, and Friedman methods, while the nucleation activity was evaluated according to the Dobreva and Gutzow approaches.

The crystalline morphology and crystal growth characteristics of the TPV samples were examined using a Nikon LV100 POL polarized optical microscope (POM) equipped with a Linkam THMS 600 heating stage. Thin-film specimens were prepared by placing the samples between glass slides and heating them at 220 °C for 2 min. Prior to the crystallization experiments, the samples were heated to 220 °C and held for 5 min to erase their thermal history. The specimens were then cooled to 115 °C at a cooling rate of 5 °C/min and maintained under isothermal conditions while the evolution of crystalline structures was monitored. Images were recorded during continuous cooling at selected temperatures of 135, 125, and 115 °C. The obtained micrographs were used to evaluate the effects of EPDM/PP composition, coagent type, and coagent loading on nucleation density, crystal growth, and spherulitic morphology.

The crystalline structure of the TPV samples was further characterized by X-ray diffraction (XRD) using a Bruker AXS D8 Advance diffractometer. Diffraction patterns were recorded over a 2θ range of 5–50° at a scanning rate of 2°/min. The diffraction profiles were analyzed to identify the crystalline phases present in the TPVs and to evaluate the influence of OV-POSS and blend composition on crystal structure development. The overall degree of crystallinity (X_c,XRD_) was determined by separating the crystalline diffraction peaks from the amorphous scattering contribution and integrating the corresponding areas. The crystalline contributions associated with the α- and β-forms of PP were also evaluated separately based on their integrated diffraction peak areas. The calculated crystallinity values were used to assess the effects of blend composition, coagent type, and coagent loading on crystalline structure development. The XRD findings were correlated with the DSC and POM results to provide a comprehensive assessment of the crystallization behavior of the investigated TPV systems.

## 3. Results and Discussion

### 3.1. Non-Isothermal Crystallization Kinetics of the TPVs

**Figure 1:**
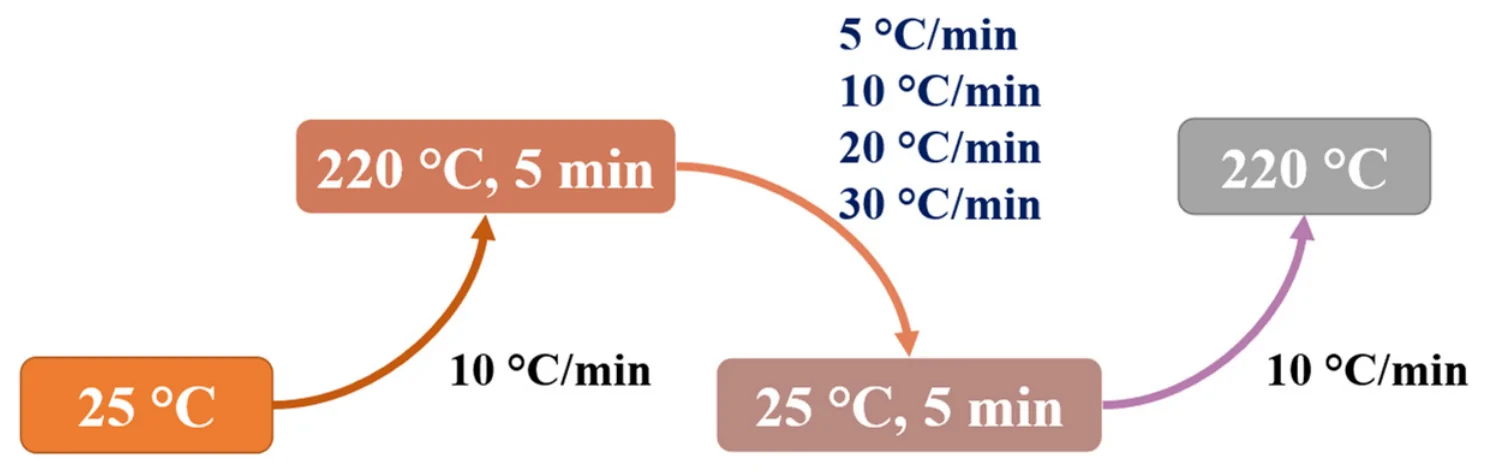
Procedure followed in DSC analyses for the study of non-isothermal crystallization kinetics.

In this part of the study, the non-isothermal crystallization kinetics of neat PP, 30/70 and 70/30 EPDM/PP blends, EPDM/PP/TAC TPVs, and EPDM/PP/OV-POSS TPVs prepared under the processing conditions of 175 °C and 100 rpm using peroxide-initiated dynamic vulcanization were investigated. The thermal protocol employed for the DSC measurements is schematically illustrated in Figure 1.

**Figure 2:**
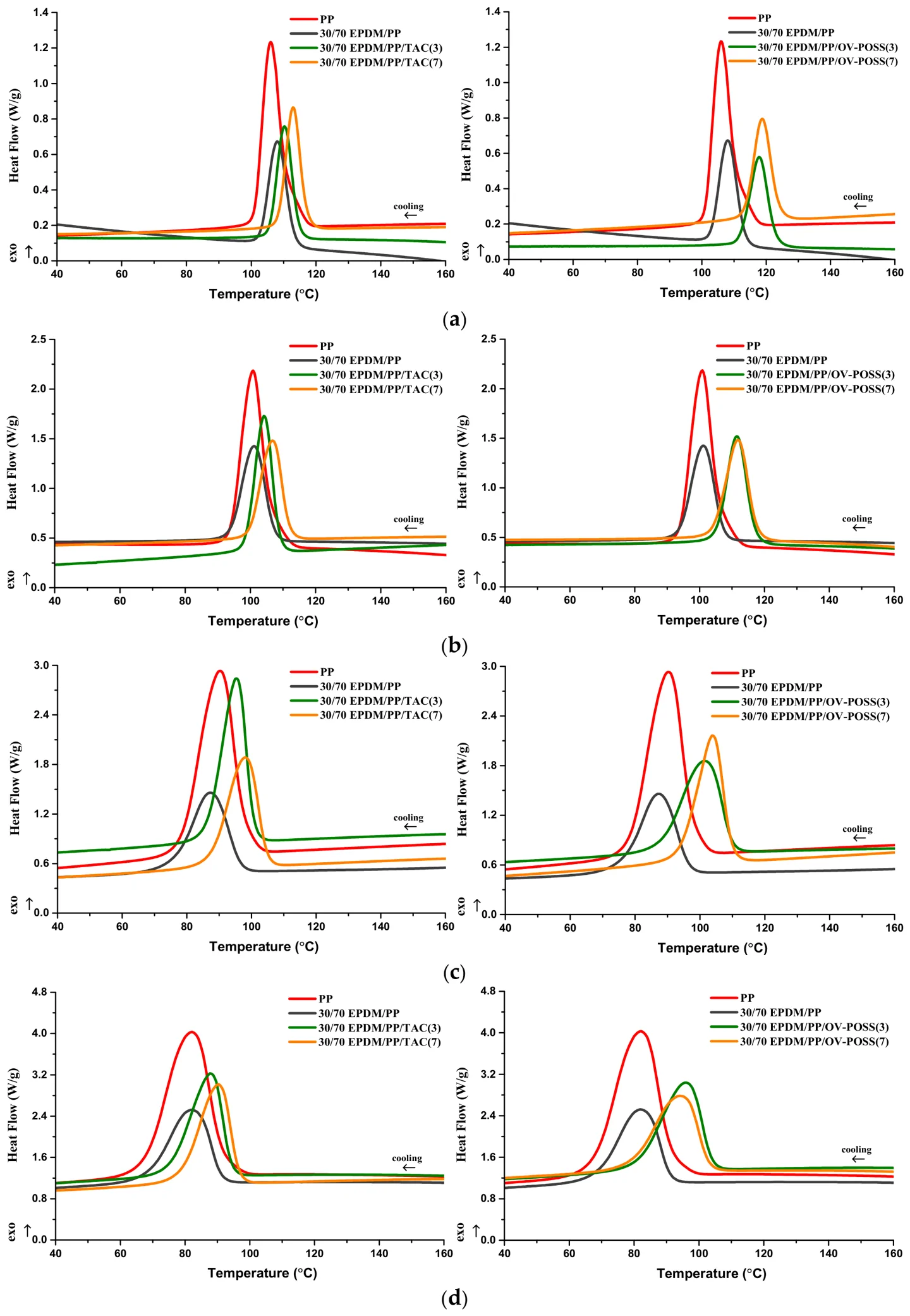
DSC cooling thermograms of neat PP, 30/70 EPDM/PP, EPDM/PP/TAC, and EPDM/PP/OV-POSS TPVs obtained at cooling rates of (**a**) 5 °C/min, (**b**) 10 °C/min, (**c**) 20 °C/min, and (**d**) 30 °C/min.

The non-isothermal crystallization thermograms of neat PP, 30/70 EPDM/PP blend, and the corresponding TPVs prepared with TAC or OV-POSS at coagent-to-DCP mass ratios of 3 and 7, obtained at different cooling rates are shown in Figure 2. The characteristic crystallization parameters, including the onset crystallization temperature (T_c,o_), peak crystallization temperature (T_c,p_), end crystallization temperature (T_c,e_), crystallization interval (ΔT = T_c,o_ − T_c,e_), and crystallization half-time (*t_1/2_*), were determined from the DSC cooling curves and are summarized in Table 2.

As can be seen from the DSC results, increasing the cooling rate shifted the melt crystallization exotherms toward lower temperatures for all formulations. Accordingly, the values of T_c,o_, T_c,p_, and T_c,e_ gradually decreased with increasing cooling rate, indicating that a greater degree of supercooling was required to initiate and complete crystallization. At the same time, the difference between the onset and end crystallization temperatures (ΔT) increased significantly, leading to a broader crystallization peak. This behavior is commonly observed in semicrystalline polymers and is attributed to the reduced time available for polymer chain diffusion, segmental rearrangement, and crystal growth during rapid cooling.

In semicrystalline polymer systems, crystallization involves the transformation of randomly distributed molecular chains in the melt into ordered crystalline structures. When the cooling rate is increased, polymer chains are unable to reorganize sufficiently to follow the rapid temperature decrease, resulting in delayed crystallization and broader crystallization exotherms. Moreover, the progressive increase in melt viscosity during rapid cooling restricts chain mobility, thereby reducing crystal growth efficiency and extending the overall crystallization process. Consequently, crystallization occurs at lower temperatures and over a wider temperature range as the cooling rate increases.

**Figure 3:**
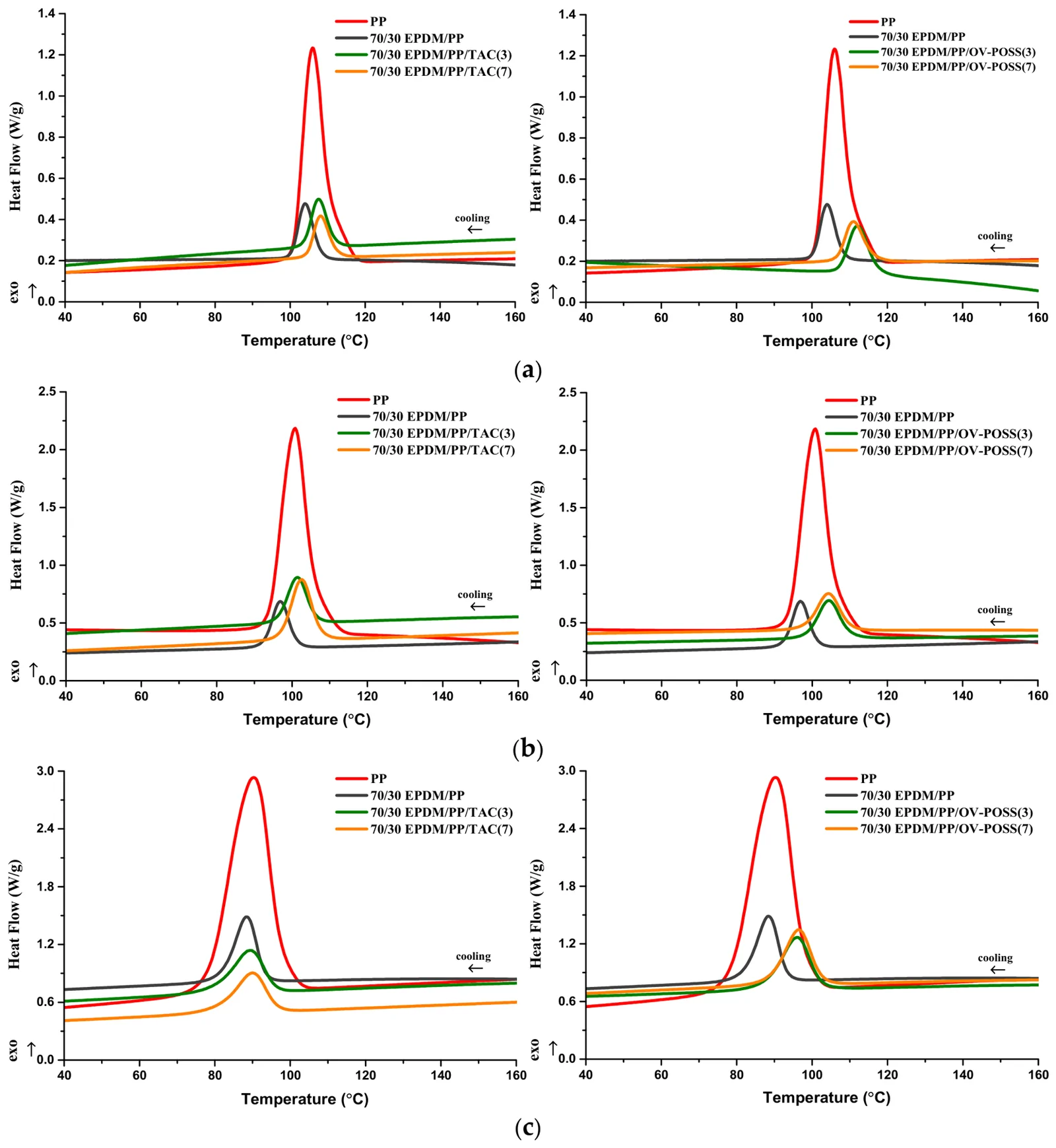

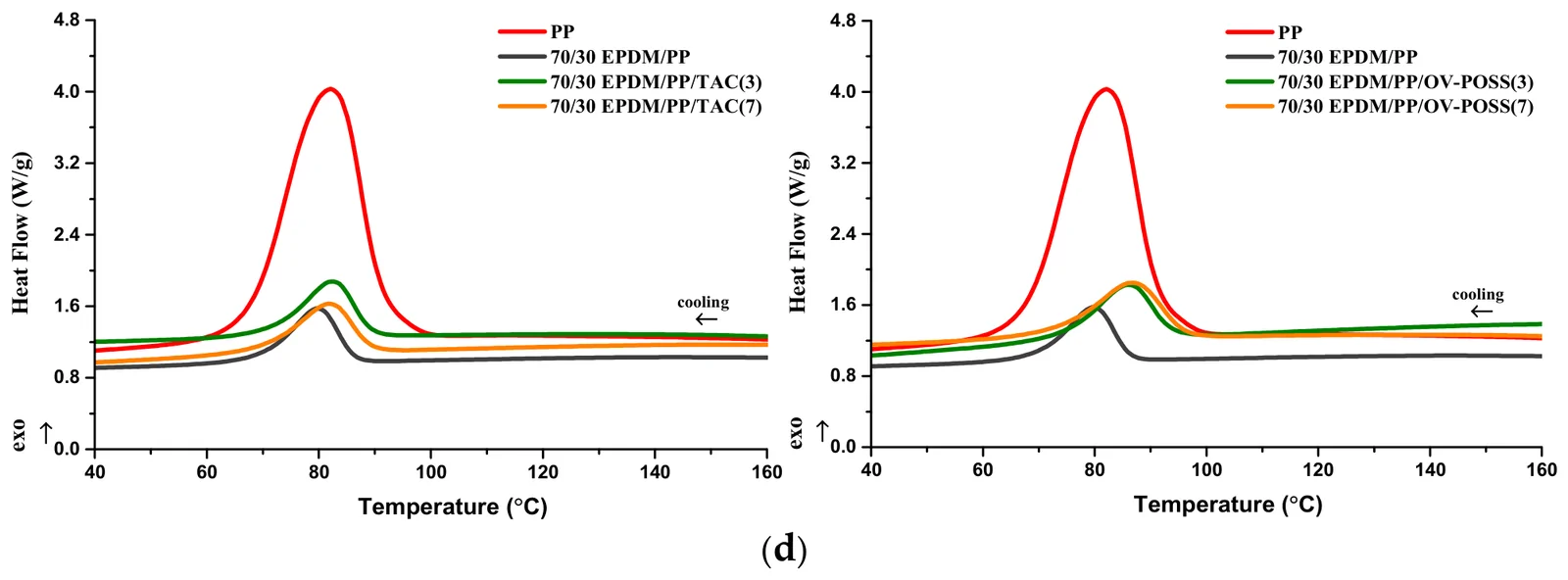
DSC cooling thermograms of neat PP, 70/30 EPDM/PP, EPDM/PP/TAC, and EPDM/PP/OV-POSS TPVs obtained at cooling rates of (**a**) 5 °C/min, (**b**) 10 °C/min, (**c**) 20 °C/min, and (**d**) 30 °C/min.

The non-isothermal crystallization thermograms of neat PP, 70/30 EPDM/PP blend, EPDM/PP/TAC TPVs, and EPDM/PP/OV-POSS TPVs obtained at different cooling rates are presented in Figure 3. The corresponding crystallization parameters calculated from the cooling thermograms are summarized in Table 3.

The DSC results obtained at different cooling rates for neat PP, the 70/30 EPDM/PP blend, and the corresponding TPVs prepared with TAC or OV-POSS at coagent-to-DCP mass ratios of 3 and 7 are presented in Table 3. Examination of the data obtained from the DSC thermograms revealed that, similar to the results obtained for the 30/70 EPDM/PP composition, the crystallization onset temperature (T_c,o_) shifted to lower temperatures with increasing cooling rate for all samples. In addition, the difference between the crystallization onset and end temperatures increased, resulting in a broader crystallization peak.

As can be seen from Table 2, compared with neat PP, melt crystallization of PP in the presence of 30 phr EPDM (30/70 EPDM/PP) was found to start at lower temperatures. This behavior can be attributed to the restriction of intermolecular chain mobility resulting from the incorporation of EPDM into the PP matrix.

The incorporation of TAC and OV-POSS at coagent-to-DCP mass ratios of 3 and 7 into the 30/70 EPDM/PP formulations resulted in higher T_c,o_ values. In particular, samples containing higher coagent loadings exhibited crystallization onset temperatures shifted toward higher values at any given cooling rate. A comparison between OV-POSS and TAC further demonstrated that OV-POSS possesses a greater ability to act as a nucleating agent. Moreover, as previously discussed based on the rheological, morphological, and mechanical characterization results, the dispersed EPDM phase was distributed more homogeneously within the PP matrix as smaller particles, especially in the presence of OV-POSS [13]. This morphology increased the number of active sites available for heterogeneous nucleation. Consequently, the onset temperature of melt crystallization shifted to considerably higher temperatures in the presence of OV-POSS compared with TAC. Similar findings have also been reported in the literature [10].

This behavior can be attributed to the ability of OV-POSS to act as a heterogeneous nucleating agent, thereby increasing the nucleation rate of PP. In summary, it can be concluded that TAC and OV-POSS at all investigated loading levels, exhibited a nucleating effect in TPVs containing a 30/70 EPDM/PP composition.

It should be noted, however, that the increase in T_c,o_ was not accompanied by a uniform reduction in *t_1/2_* or ΔT (Table 2). While TAC(3) exhibited shorter *t_1/2_* and narrower ΔT than the blend at all cooling rates, the OV-POSS-containing TPVs, particularly OV-POSS(7), showed comparable or longer *t_1/2_* and broader ΔT at low cooling rates. Because crystallization in these samples begins at a lower degree of supercooling, the lamellar growth rate in the early stage is reduced. In addition, the higher crosslink densities of the OV-POSS-containing TPVs [13] and the earlier impingement of densely nucleated crystals may restrict subsequent growth. Thus, T_c,o_ primarily reflects nucleation, whereas *t_1/2_* and ΔT reflect the combined contributions of nucleation and growth.

As shown in Table 3, compared with neat PP, the 70/30 EPDM/PP blend exhibited a noticeably higher apparent melt crystallization rate, lower ΔT values, and lower crystallization onset temperatures. Although the higher EPDM content reduced the crystallization enthalpy due to the lower PP fraction, the PP-normalized degree of crystallinity remained comparable between the two reference blends. Nevertheless, the narrower crystallization interval and shorter crystallization half-time observed for the EPDM-rich formulation indicate differences in the overall crystallization kinetics, which should be distinguished from the final degree of crystallinity.

The incorporation of TAC or OV-POSS at coagent-to-DCP mass ratios of 3 and 7 into the 70/30 EPDM/PP samples resulted in higher T_c,o_ values. When OV-POSS and TAC were compared, the crystallization onset temperatures of the OV-POSS-containing samples were shifted to higher temperatures at all applied cooling rates, indicating a stronger nucleating effect of OV-POSS (Table 3). Only slight changes in T_c,o_ values were observed with increasing OV-POSS loading. These results suggest that OV-POSS acts as a heterogeneous nucleating agent and that its nucleating efficiency is particularly pronounced at lower loading levels.

The effect of OV-POSS should be considered in relation to the distinct starting morphologies of the two blend compositions. In the PP-rich 30/70 system, PP already forms the continuous phase, and the refinement of dispersed EPDM domains in the presence of OV-POSS may increase the number of interfaces at which PP crystallization begins. In the EPDM-rich 70/30 system, dynamic vulcanization additionally induces phase inversion, converting the initially continuous EPDM phase into dispersed crosslinked domains within PP [13]. Consequently, OV-POSS can affect both the development of the continuous PP phase and the density of potential nucleation sites in this composition. Although the OV-POSS-containing TPVs generally show earlier crystallization onset than their TAC-containing counterparts, the magnitude of this effect and the subsequent overall crystallization rate remain dependent on composition, loading, and cooling rate. Thus, the more pronounced morphological reorganization in the 70/30 system provides a plausible explanation for its sensitivity to coagent selection, rather than evidence that OV-POSS accelerates every stage of crystallization. A similar competition between heterogeneous nucleation and restricted chain mobility was discussed by Wang et al. [12] for concentrated EPDM/PP TPVs. This comparison supports distinguishing an earlier crystallization onset from faster overall conversion, without implying that the same mechanism has been directly established in the present formulations.

In addition to the crystallization temperatures and kinetic parameters presented in Table 2 and Table 3, the degree of crystallinity of the PP phase was evaluated to further investigate the influence of blend composition, coagent type, and coagent-to-DCP ratio on the extent of crystallization. The DSC-based crystallinity (X_c,DSC_) was calculated from the melting enthalpy (ΔH_m_) obtained during the second heating scan recorded at a heating rate of 10 °C/min, using the following Equation (1):

(1) $$X_{c,DSC}\left(\%\right)=\frac{\Delta H_{m}}{w_{PP}\times \Delta H_{m}^{0}}\times 100$$

where ΔH_m_ is the melting enthalpy (J/g), w_PP_ is the PP mass fraction in the total formulation, and $\Delta H_{m}^{0}$ is the theoretical melting enthalpy of 100% crystalline PP, taken as 209 J/g [24]. The PP mass fraction was determined by considering all formulation components, including the paraffinic oil present in the commercial EPDM. The calculated crystallinity values are summarized in Table 4.

As shown in Table 4, the reference 30/70 and 70/30 EPDM/PP blends exhibited relatively comparable PP-normalized crystallinity values of 35.1% and 32.7%, respectively, despite their substantially different melting enthalpies. This indicates that the difference in melting enthalpy was largely associated with the different PP contents, while the proportion of crystalline PP was only moderately affected by blend composition.

The incorporation of coagents influenced the crystallinity of both EPDM/PP systems, although the extent of the effect depended on the coagent type and loading. TAC addition resulted in moderate increases in crystallinity, particularly in the PP-rich 30/70 formulations, while relatively minor changes were observed in the EPDM-rich 70/30 system. In contrast, OV-POSS generally produced a more pronounced increase in crystallinity, especially at the higher coagent-to-DCP ratio. For the 30/70 and 70/30 formulations, the crystallinity increased from 35.1% and 32.7% for the reference blends to 41.9% and 40.5% for OV-POSS(7), respectively. These changes corresponded to increases of 6.8 and 7.8 percentage points, indicating that OV-POSS promoted crystalline structure development in both compositions.

The higher crystallinity observed in the OV-POSS-containing TPVs may be associated with changes in heterogeneous nucleation and phase morphology during dynamic vulcanization. However, the final degree of crystallinity reflects the combined effects of nucleation and subsequent crystal growth. Therefore, the increased crystallinity should not be attributed exclusively to enhanced nucleation without considering possible changes in polymer-chain mobility and crystal growth behavior.

While the DSC-based degree of crystallinity determined from melting enthalpy provides information on the crystalline fraction of the PP phase prior to heating, the relative crystallinity, X(t), describes the progress of crystallization during cooling as a function of temperature or time. Accordingly, the non-isothermal crystallization process was further evaluated using the relative crystallinity calculated from the DSC cooling exotherms according to Equation (2).

**Figure 4:**
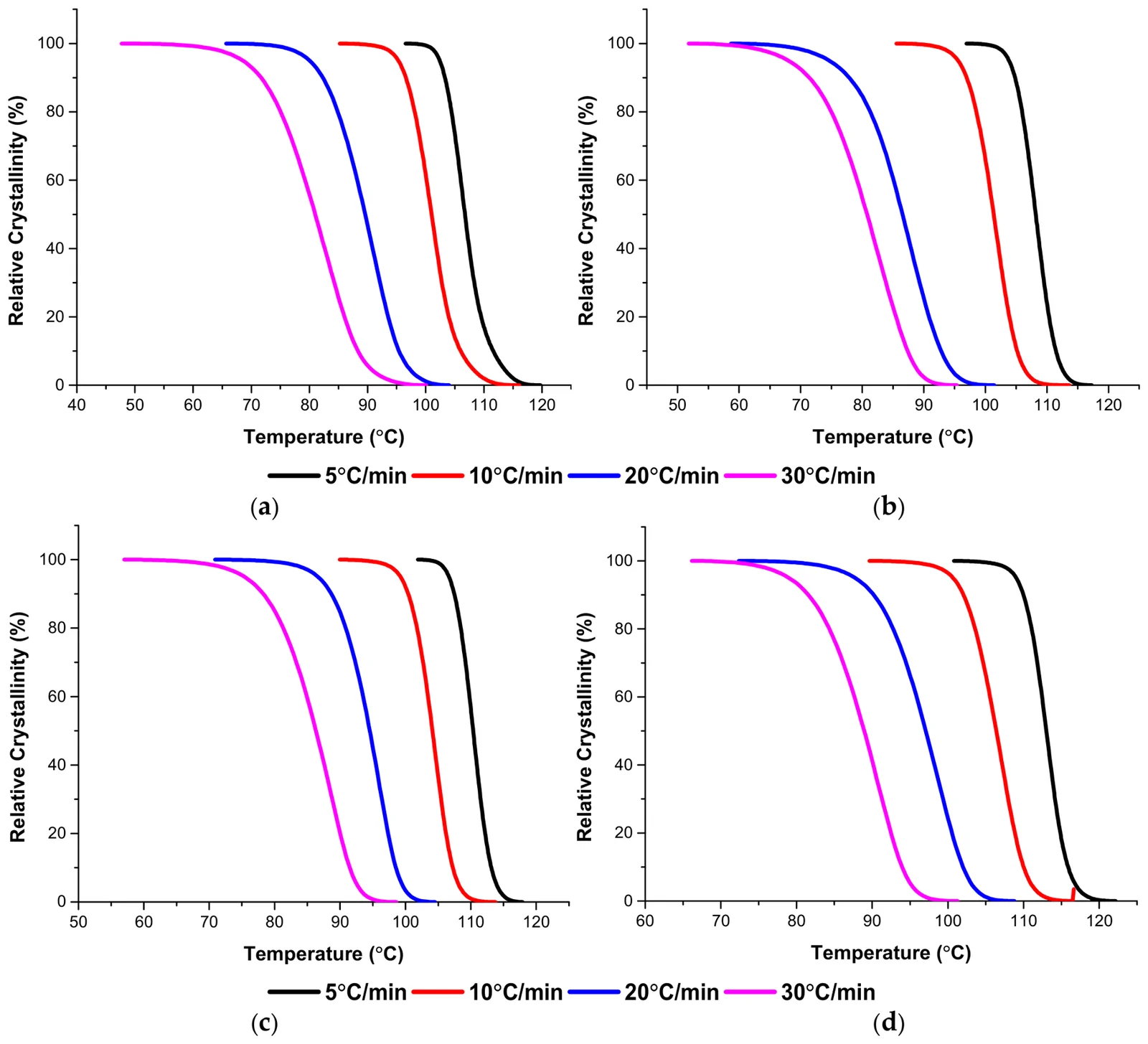

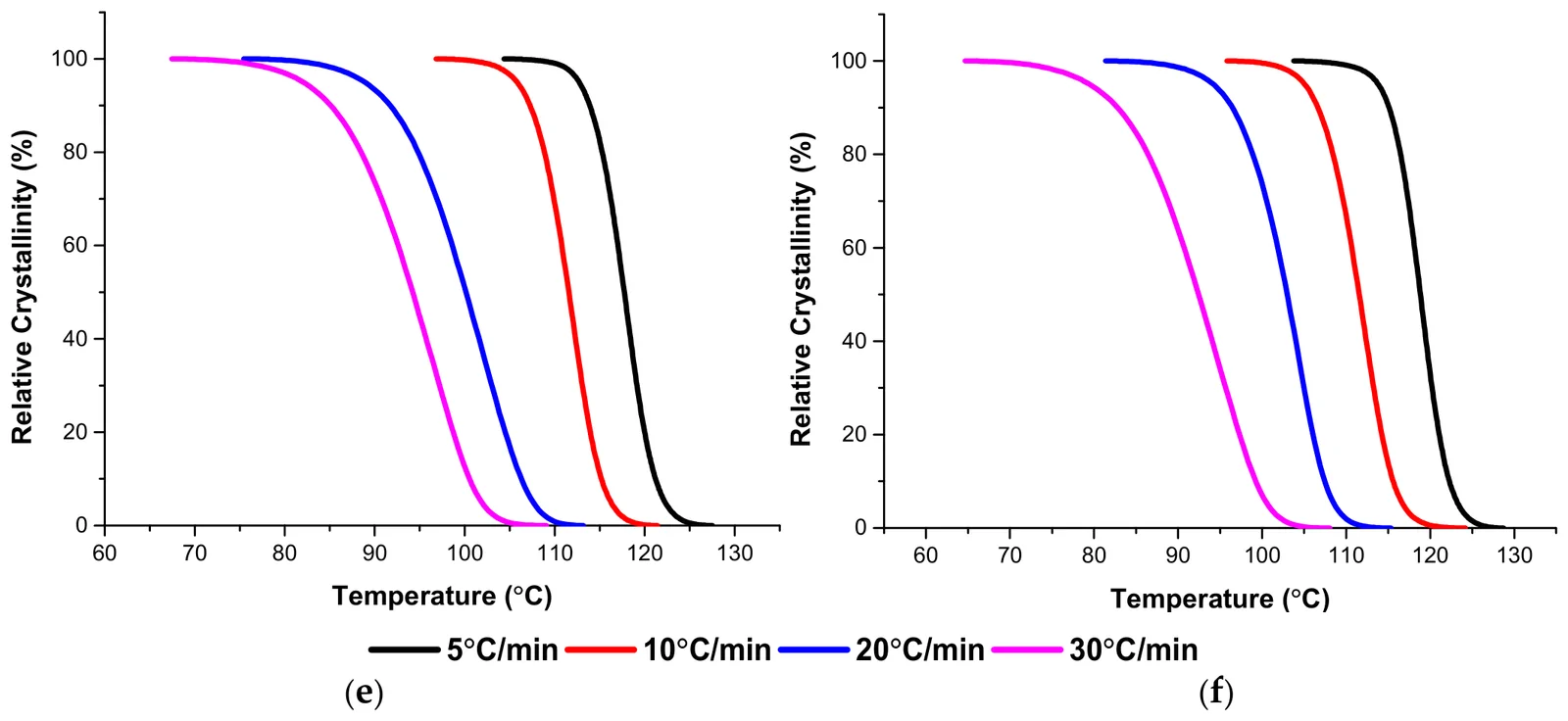
Variation in relative crystallinity with temperature at different cooling rates for 30/70 EPDM/PP TPVs: (**a**) PP, (**b**) EPDM/PP, (**c**) EPDM/PP/TAC(3), (**d**) EPDM/PP/TAC(7), (**e**) EPDM/PP/OV-POSS(3), and (**f**) EPDM/PP/OV-POSS(7).

**Figure 5:**
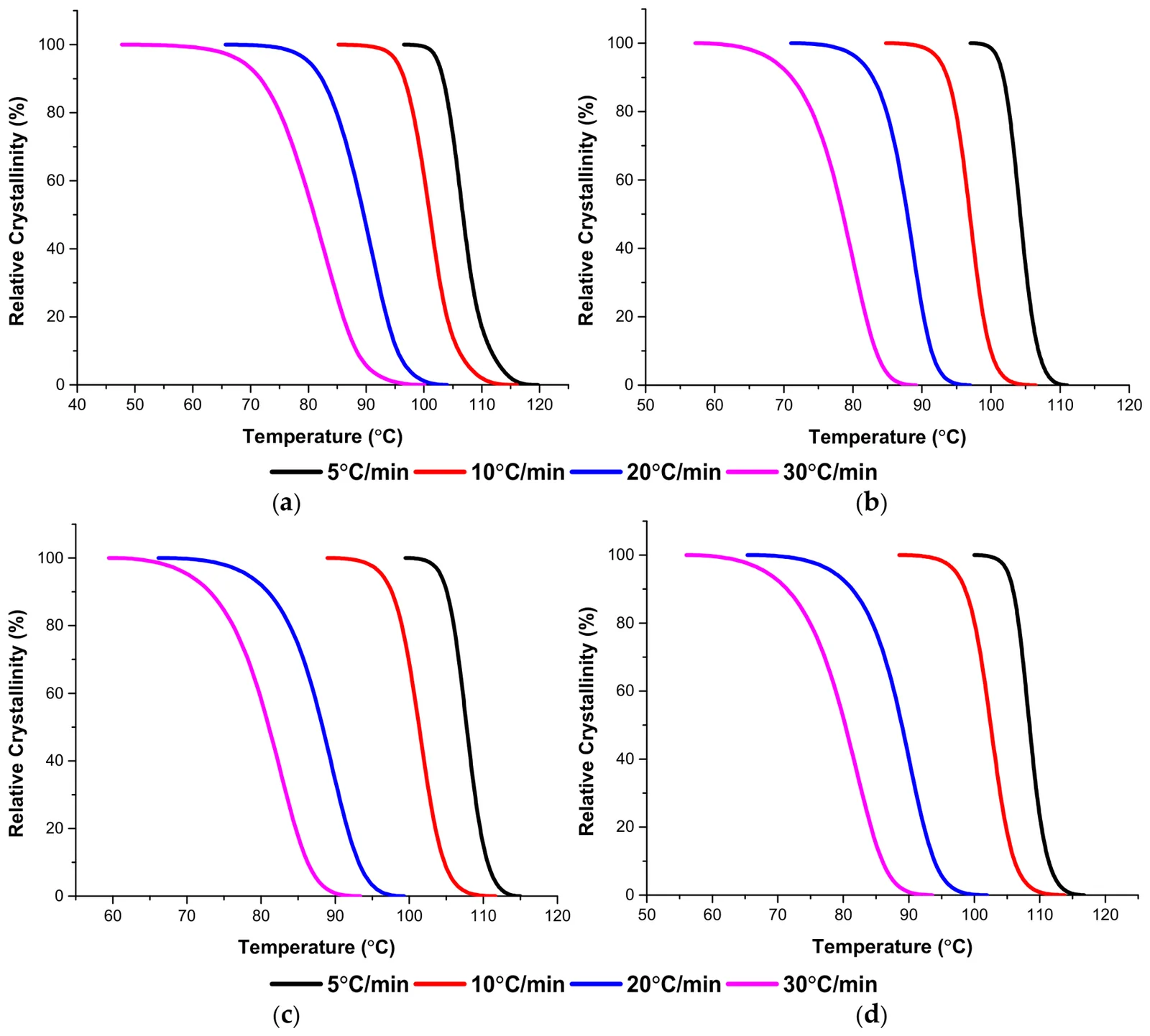

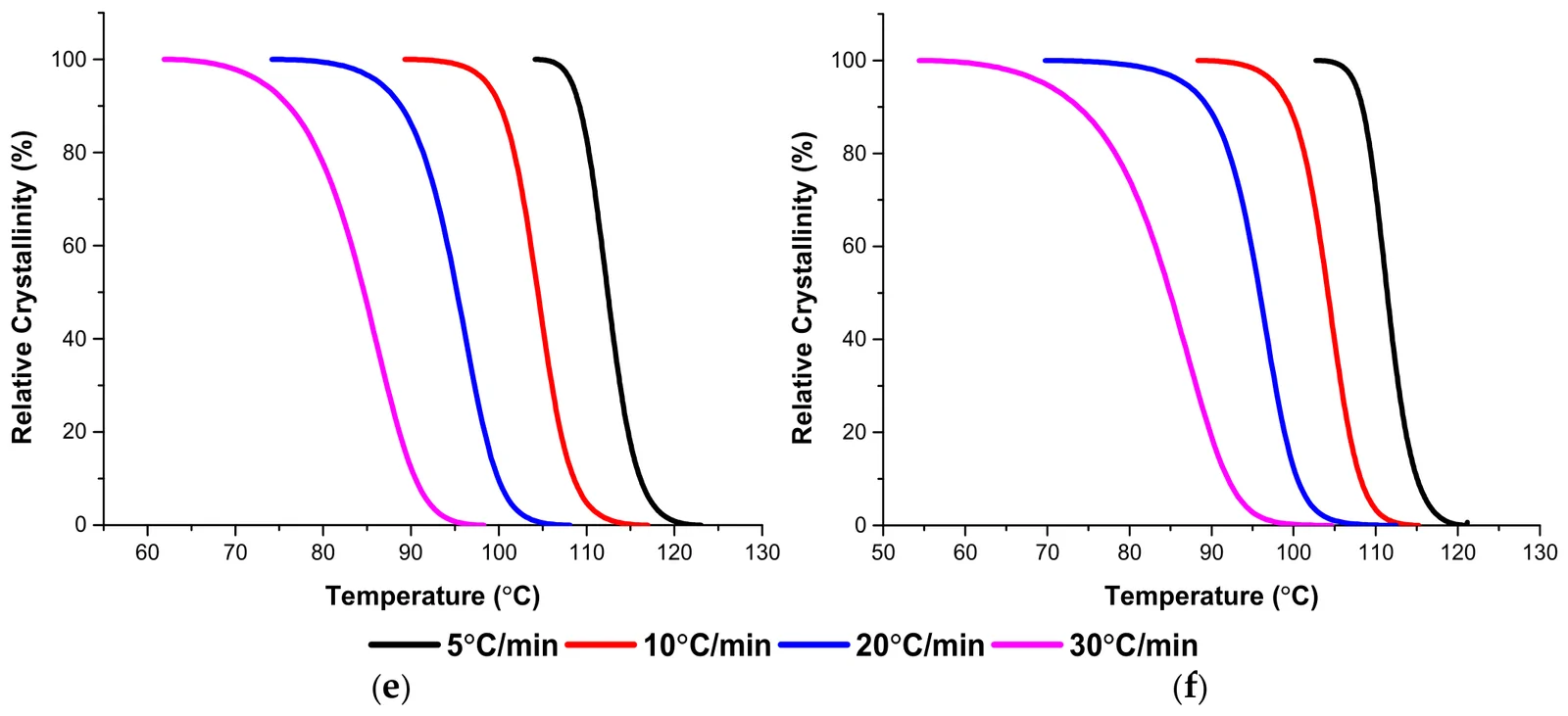
Variation in relative crystallinity with temperature at different cooling rates for 70/30 EPDM/PP TPVs: (**a**) PP, (**b**) EPDM/PP, (**c**) EPDM/PP/TAC(3), (**d**) EPDM/PP/TAC(7), (**e**) EPDM/PP/OV-POSS(3), and (**f**) EPDM/PP/OV-POSS(7).

**Figure 6:**
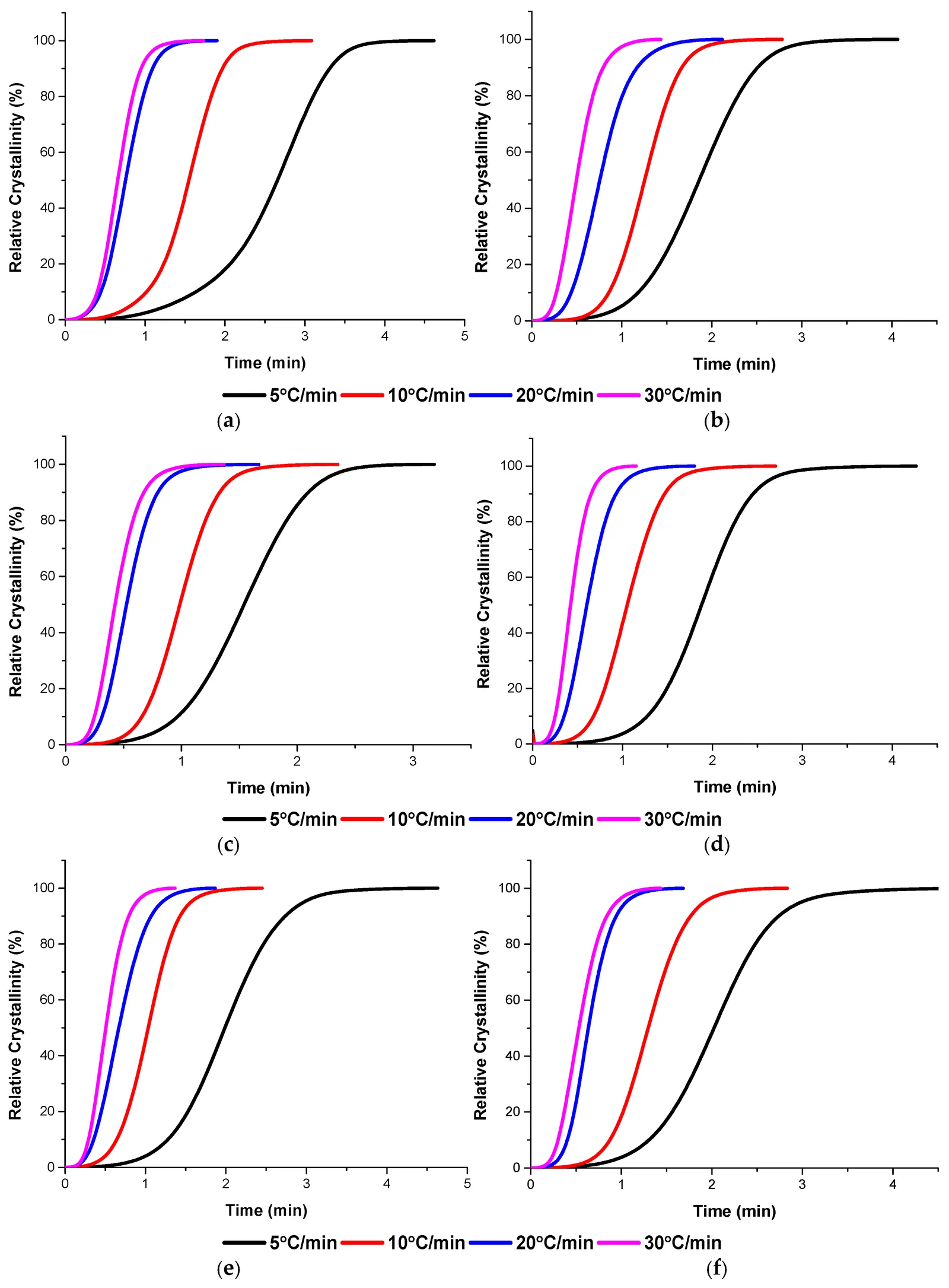
Variation in relative crystallinity with time at different cooling rates for 30/70 EPDM/PP TPVs: (**a**) PP, (**b**) EPDM/PP, (**c**) EPDM/PP/TAC(3), (**d**) EPDM/PP/TAC(7), (**e**) EPDM/PP/OV-POSS(3), and (**f**) EPDM/PP/OV-POSS(7).

**Figure 7:**
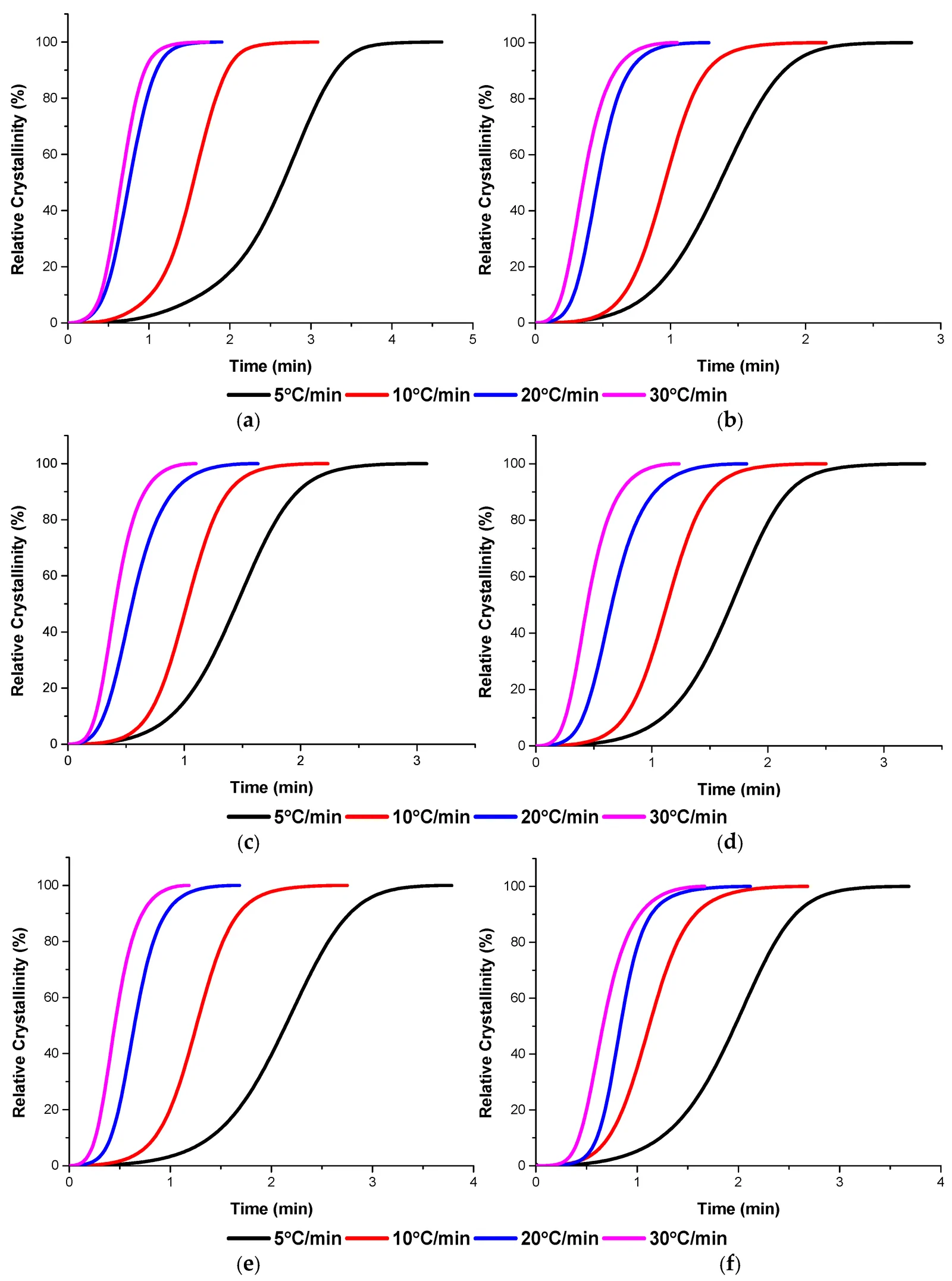
Variation in relative crystallinity with time at different cooling rates for 70/30 EPDM/PP TPVs: (**a**) PP, (**b**) EPDM/PP, (**c**) EPDM/PP/TAC(3), (**d**) EPDM/PP/TAC(7), (**e**) EPDM/PP/OV-POSS(3), and (**f**) EPDM/PP/OV-POSS(7).

Based on non-isothermal crystallization analyses, the relative crystallinity, X(t), as a function of crystallization temperature (T) was calculated using Equation (2):

(2) $$X\left(T\right)=\int_{T_{0}}^{T}\left(\frac{dH_{c}}{\mathrm{dT}}\right)\mathrm{dT}/\int_{T_{0}}^{T_{\infty }}\left(\frac{dH_{c}}{\mathrm{dT}}\right)\cdot \;\mathrm{dT}$$

where T_0_ and T_∞_ represent the onset and end temperatures of crystallization, respectively. T denotes any temperature during the crystallization process, while dH/dT represents the heat flow at temperature T. The relative crystallinity values as functions of temperature and time at different cooling rates are presented in Figure 4, Figure 5, Figure 6 and Figure 7.

Figure 4 shows the variation in relative crystallinity with temperature for neat PP, 30/70 EPDM/PP, and EPDM/PP/TAC and EPDM/PP/OV-POSS TPVs at different cooling rates. Similarly, Figure 5 illustrates the temperature dependence of relative crystallinity for neat PP, 70/30 EPDM/PP, and EPDM/PP/TAC and EPDM/PP/OV-POSS TPVs under various cooling conditions.

All curves obtained for neat PP, EPDM/PP blends, and TPVs exhibit a characteristic sigmoidal shape. The initial plateau region observed in the early stage of crystallization corresponds to the induction period, during which crystal nuclei are formed. Subsequently, crystal growth begins, accelerating the crystallization process until the growing crystals impinge upon one another. Once crystal impingement occurs, the crystallization rate decreases, which is reflected by the declining slope in the upper part of the curves and the appearance of a second plateau region. This region corresponds to secondary crystallization.

With increasing cooling rate, the crystallization temperature shifted toward lower temperatures, and the crystallization temperature range became broader. In general, at a given cooling rate, the TPVs containing OV-POSS exhibited higher crystallization temperatures than the other samples (Figure 4 and Figure 5). These findings are also consistent with the results presented in Table 2 and Table 3.

For a non-isothermal crystallization process at a constant cooling rate, the relationship between crystallization temperature, T, and crystallization time, t, can be expressed by Equation (3):

(3) $$t=\frac{T_{0}-T}{\beta }$$

where t is the crystallization time, T is the crystallization temperature at time t, and β is the cooling rate. In addition, this equation can be used to calculate the crystallization half-time (*t_1/2_*), which is defined as the time required to achieve 50% relative crystallinity.

Figure 6 presents the variation in X(t) with time for neat PP, 30/70 EPDM/PP, and EPDM/PP/TAC and EPDM/PP/OV-POSS TPVs at different cooling rates, whereas Figure 7 shows the corresponding curves for neat PP, 70/30 EPDM/PP, and EPDM/PP/TAC and EPDM/PP/OV-POSS TPVs.

As can be seen from Figure 6 and Figure 7, for neat PP as well as all blend compositions, the time required to attain the same X(t) value increases with decreasing cooling rate. At lower cooling rates, crystallization starts at higher temperatures and proceeds over a longer period. This behavior is likely attributable to the fact that slower cooling provides polymer chains with sufficient time to adopt the conformational arrangements necessary for crystallization, thereby promoting the formation of more ordered crystalline structures [25].

Furthermore, compared with neat PP, the crystallization times of both the 30/70 and 70/30 EPDM/PP blends and their corresponding TPVs were generally shortened at all cooling rates. The shorter crystallization times of the EPDM/PP blends indicate differences in overall crystallization kinetics rather than direct evidence of enhanced nucleation by EPDM. In the coagent-containing TPVs, morphological changes and the incorporation of TAC or OV-POSS may further influence crystallization behavior. For the 70/30 EPDM/PP systems, the shorter crystallization half-times and narrower crystallization intervals suggest that crystallization proceeded over a more restricted temperature range and within a shorter period, without necessarily indicating suppression of PP crystal growth by EPDM.

The Avrami equation was originally developed to describe isothermal crystallization and has also been applied to non-isothermal polymer crystallization as an approximate analytical approach [26,27,28]. In this study, linear regression of ln[−ln(1 − X(t))] against ln t was performed over the relative crystallinity range of 20-80% for all samples at each cooling rate. The same conversion range was used to provide a consistent basis for comparison among formulations. The Avrami equation can be expressed as follows (Equation (4)):

(4) $$1-X(t)=exp(-Zt^{n})$$

where X(t) is the relative crystallinity at time t, Z is the crystallization rate constant associated with nucleation and crystal growth, and n is the Avrami exponent.

To determine the values of the rate constant and the Avrami exponent, Equation (4) is transformed into its double-logarithmic form, as given in Equation (5):

(5) ln(−ln(1−X(t)))=lnZ+n⋅lnt

By plotting ln[−ln(1 − X(t))] against ln t, n is obtained from the slope, whereas the intercept corresponds to ln Z. The value of Z is therefore calculated by exponentiating the intercept.

The Avrami equation represented by Equation (4) was originally developed for isothermal crystallization conditions. To account for the influence of temperature under non-isothermal crystallization conditions, Jeziorny modified the Avrami model by correcting the crystallization rate constant, Z, with respect to the cooling rate, yielding the modified Avrami equation shown in Equation (6):

(6) $$InZ_{c}=InZ/\beta$$

**Figure 8:**
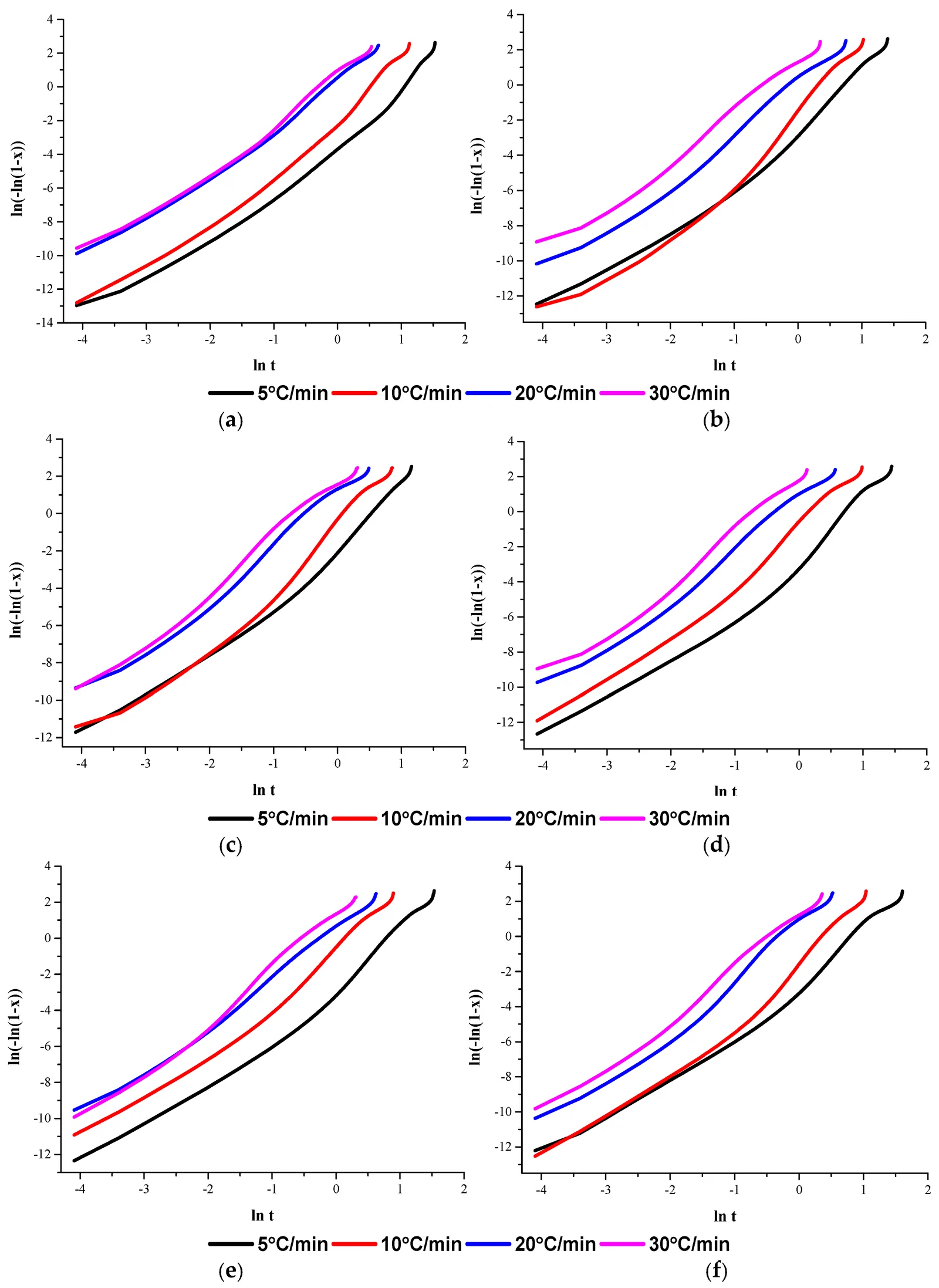
Avrami plots of 30/70 EPDM/PP TPVs: (**a**) PP, (**b**) EPDM/PP, (**c**) EPDM/PP/TAC(3), (**d**) EPDM/PP/TAC(7), (**e**) EPDM/PP/OV-POSS(3), and (**f**) EPDM/PP/OV-POSS(7).

**Figure 9:**
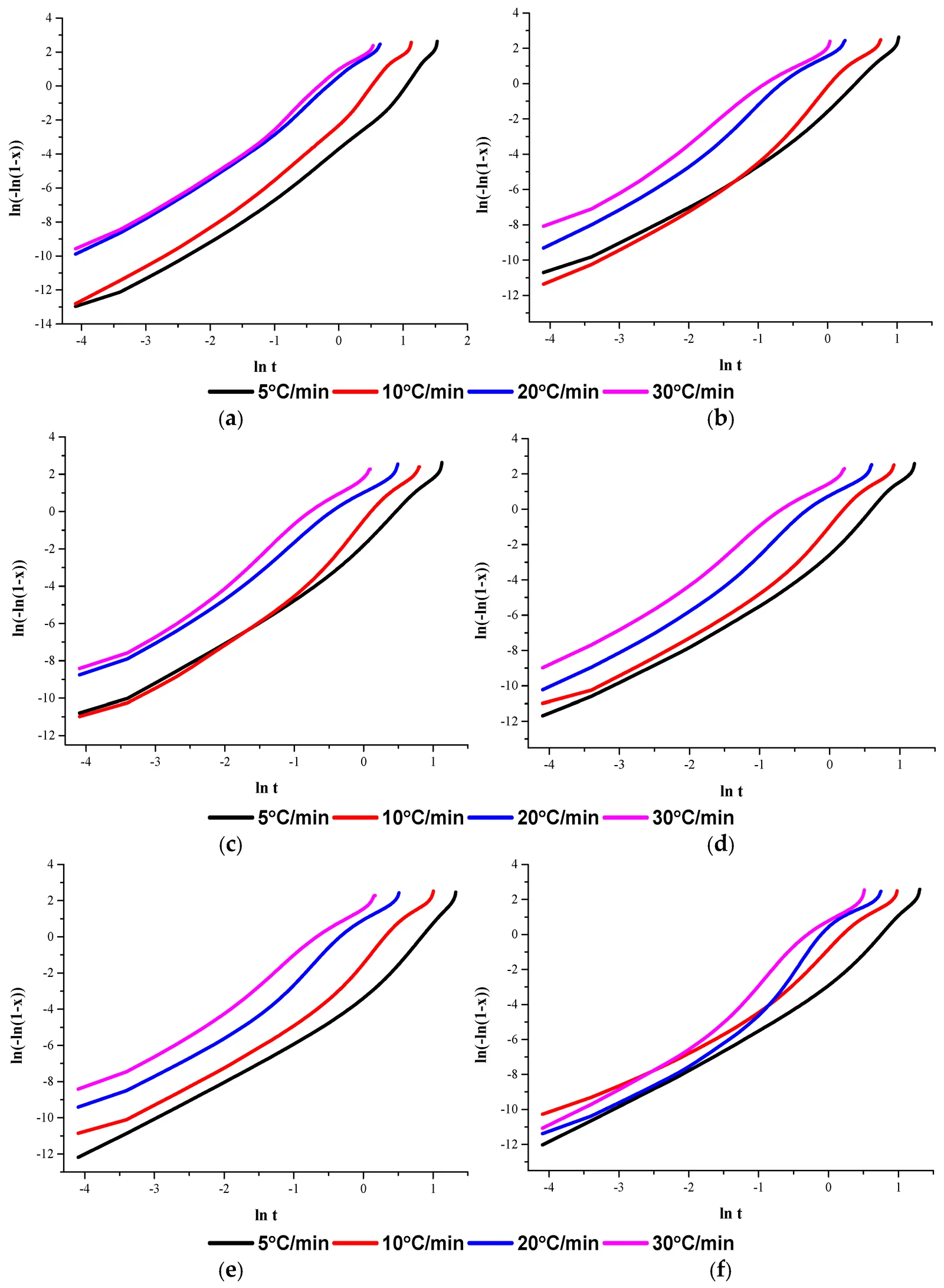
Avrami plots of 70/30 EPDM/PP TPVs: (**a**) PP, (**b**) EPDM/PP, (**c**) EPDM/PP/TAC(3), (**d**) EPDM/PP/TAC(7), (**e**) EPDM/PP/OV-POSS(3), and (**f**) EPDM/PP/OV-POSS(7).

The Avrami plots obtained for neat PP, the 30/70 EPDM/PP blend, and the corresponding TPVs are presented in Figure 8, while those for neat PP, the 70/30 EPDM/PP blend, and the corresponding TPVs are shown in Figure 9.

Avrami plots may exhibit regions with different slopes as crystallization progresses. Primary crystallization involves nucleation and crystal growth, which contribute to the macroscopic development of crystallinity [29]. At later stages, secondary crystallization may involve further ordering and reorganization of the crystalline structures. Under non-isothermal conditions, however, changes in slope and departures from linearity cannot be attributed exclusively to secondary crystallization, because temperature-dependent changes in nucleation and growth may also affect the plots. In this study, the analysis was performed over the same relative crystallinity range of 20–80% for all samples and cooling rates. The fitted parameters were therefore interpreted as apparent descriptors of crystallization within this interval, and a specific crystal-growth geometry was not assigned solely on the basis of the Avrami exponent.

Since the dimensions of the Avrami rate constant depend on the value of the Avrami exponent, direct comparison of the crystallization kinetics based exclusively on Z or Z_c_ may be misleading. Therefore, the Avrami-derived crystallization half-time, *t_0.5_* defined as the time required to reach 50% relative crystallinity according to the fitted Avrami equation, was calculated using Equation (7) [30]:

(7) $$t_{0.5}={\left(\frac{\ln2}{Z}\right)}^{1/n}$$

where *Z* is the Avrami crystallization rate constant and n is the Avrami exponent. A lower *t_0.5_* value indicates a higher overall crystallization rate [30]. The Avrami-derived *t_0.5_* should be distinguished from the experimental crystallization half-time, *t_1/2_*, determined directly from the DSC relative crystallinity–time curves. While *t_1/2_* represents the experimentally observed time required to reach 50% relative crystallinity, *t_0.5_* is calculated from the kinetic parameters obtained by fitting the primary crystallization region to the Avrami equation.

Table 5 summarizes the apparent Avrami exponent (n), rate constant (Z), Jeziorny-corrected parameter (Z_c_), model-derived crystallization half-time (*t_0.5_*), and coefficient of determination (R^2^) for neat PP and the 30/70 EPDM/PP formulations. As shown in Table 5, The n values ranged from 2.15 to 5.11 and varied with formulation and cooling rate. These values characterize the fitted non-isothermal conversion behavior over the selected interval and should not be interpreted as direct evidence of a specific crystal-growth dimensionality or lamellar morphology.

As shown in Table 5, the Jeziorny-corrected crystallization rate constant, Z_c_, generally increased with cooling rate, whereas the Avrami-derived crystallization half-time, *t_0.5_*, decreased for all investigated formulations. For neat PP, *t_0.5_* decreased from 2.64 min at 5 °C/min to 0.66 min at 30 °C/min, while the corresponding values for the 30/70 EPDM/PP blend decreased from 1.86 to 0.50 min. These results indicate that 50% relative crystallinity was reached within a shorter time as the cooling rate increased, although crystallization shifted toward lower temperatures, as observed by DSC.

The 30/70 EPDM/PP blend exhibited shorter t*_0.5_* values than neat PP at cooling rates of 5, 10, and 30 °C/min, while both samples exhibited approximately the same value of 0.76 min at 20 °C/min. Thus, EPDM incorporation generally shortened the time required to reach 50% relative crystallinity under the investigated conditions. However, this result describes the overall relative conversion behavior and does not independently establish an increase in nucleation rate or crystal-growth rate.

At 5 °C/min, the shortest *t_0.5_* among the investigated 30/70 formulations was obtained for TAC(3), with a value of 1.54 min, compared with 1.86 min for the unvulcanized blend. Increasing the coagent-to-DCP mass ratio to 7 increased *t_0.5_* to 1.88 min, close to that of the blend. At cooling rates of 10-30 °C/min, both TAC-containing TPVs exhibited shorter *t_0.5_* values than the corresponding blend. TAC(3) exhibited shorter half-times than TAC(7) at 10 and 20 °C/min, whereas the order reversed at 30 °C/min, with values of 0.43 and 0.29 min, respectively. These results indicate that the effect of TAC loading on the apparent crystallization kinetics depended on the cooling rate.

A loading- and cooling-rate-dependent response was observed for the OV-POSS-containing TPVs. As discussed previously in relation to Table 2, the incorporation of OV-POSS shifted the crystallization temperatures toward higher values, consistent with facilitated heterogeneous nucleation of the PP phase. The finer dispersion of crosslinked EPDM domains reported previously [13] may provide additional potential nucleation sites within the PP matrix. Nevertheless, the earlier onset of crystallization was not consistently accompanied by a shorter crystallization half-time. The 30/70 EPDM/PP/OV-POSS(3) formulation exhibited shorter *t_0.5_* values than the corresponding EPDM/PP blend at cooling rates of 10–20 °C/min whereas the values at 30 °C/min were similar (0.51 and 0.50 min, respectively). At 5 °C/min, OV-POSS(3) and OV-POSS(7) exhibited *t_0.5_* values of 2.00 and 2.03 min, respectively, compared with 1.86 min for the blend and 2.64 min for neat PP. Increasing the coagent-to-DCP mass ratio from 3 to 7 increased *t_0.5_* from 1.04 to 1.30 min at 10 °C/min, whereas the values decreased slightly from 0.67 to 0.64 min at 20 °C/min and remained similar at 30 °C/min (0.51 and 0.52 min). Thus, increasing the OV-POSS loading did not produce a uniform change in the crystallization half-time. The higher crosslink density previously reported for the corresponding OV-POSS(7) formulation [13] supports a possible contribution of network-related constraints, which may hinder PP-chain rearrangement. However, this contribution alone does not explain the cooling-rate-dependent ordering of the half-times. Moreover, the formation of numerous nuclei may have caused earlier impingement of the growing crystalline structures, potentially influencing subsequent crystal growth. As the cooling rate increased from 5 to 30 °C/min, *t_0.5_* decreased from 2.00 to 0.51 min for OV-POSS(3) and from 2.03 to 0.52 min for OV-POSS(7). Thus, OV-POSS promoted crystallization onset, whereas its influence on the overall crystallization kinetics varied with loading and cooling rate. Competing contributions from nucleation and subsequent crystal development provide a possible interpretation of this response. The finer crystalline morphology observed by POM is compatible with enhanced nucleation but does not, by itself, establish a reduction in crystal-growth rate.

Overall, the Z_c_ and *t_0.5_* results indicate that the effects of coagent incorporation on the apparent crystallization kinetics of the 30/70 EPDM/PP formulations depended on coagent type, coagent-to-DCP mass ratio, and cooling rate. Increasing the coagent-to-DCP ratio did not uniformly shorten or prolong the crystallization half-time. Together with the DSC findings, these results suggest that an earlier crystallization onset does not necessarily correspond to faster subsequent conversion. Competing contributions from nucleation and crystal development may account for this behavior, although their individual effects cannot be distinguished from the fitted parameters alone.

Table 6 summarizes the apparent Avrami parameters and model-derived crystallization half-times for neat PP and the 70/30 EPDM/PP formulations. For the blend and corresponding TPVs, the n values ranged from 2.76 to 5.01 and varied with formulation and cooling rate. As discussed for the PP-rich systems, these apparent exponents describe the fitted non-isothermal conversion behavior and do not independently establish a specific crystal-growth geometry or nucleation mechanism.

For the 70/30 EPDM/PP systems, Z_c_ generally increased and *t_0.5_* decreased with increasing cooling rate. The *t_0.5_* of the unvulcanized blend decreased from 1.36 min at 5 °C/min to 0.36 min at 30 °C/min. At all the cooling rates investigated, the blend exhibited shorter half-times than neat PP, whose corresponding values decreased from 2.64 to 0.66 min. These results indicate that the EPDM-rich blend reached 50% relative crystallinity within a shorter time, without independently demonstrating enhanced nucleation or restricted crystal growth.

The incorporation of TAC generally increased *t_0.5_* relative to the corresponding 70/30 EPDM/PP blend. At 5 °C/min, *t_0.5_* increased from 1.36 min for the blend to 1.45 and 1.68 min for TAC(3) and TAC(7), respectively. An exception was observed for TAC(3) at 10 °C/min, where *t_0.5_* was 0.84 min compared with 0.96 min for the blend. At 30 °C/min, the values were 0.41 and 0.45 min for TAC(3) and TAC(7), respectively, compared with 0.36 min for the blend. TAC(7) exhibited longer half-times than TAC(3) at all investigated cooling rates. Therefore, although TAC increased the crystallization onset temperature, consistent with facilitated heterogeneous nucleation, it did not consistently enhance the overall crystallization rate of the EPDM-rich TPVs.

A loading and cooling rate dependent response was observed for the OV-POSS containing formulations. Increasing the coagent-to-DCP mass ratio from 3 to 7 decreased *t_0.5_* from 2.14 to 1.96 min at 5 °C/min and from 1.26 to 1.12 min at 10 °C/min. Conversely, longer half-times were obtained for OV-POSS(7) than for OV-POSS(3) at 20 and 30 °C/min. At 30 °C/min, *t_0.5_* was 0.67 min for OV-POSS(7), compared with 0.46 min for OV-POSS(3) and 0.36 min for the unvulcanized blend. Both OV-POSS-containing formulations exhibited longer half-times than the corresponding blend at all investigated cooling rates, indicating that facilitated crystallization onset did not translate into a shorter time to reach 50% relative crystallinity.

**Figure 10:**
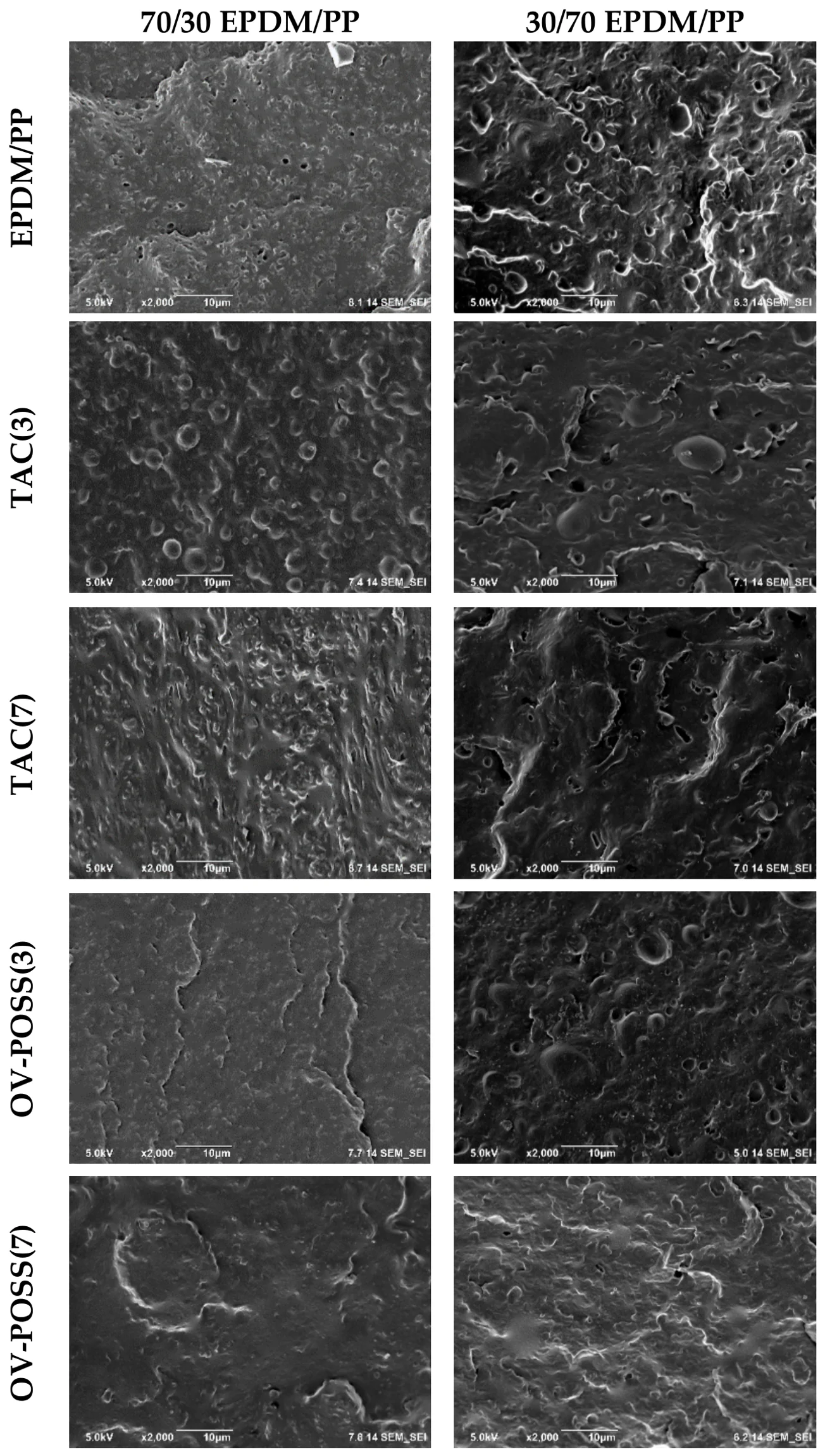
SEM micrographs of cryogenically fractured surfaces of the 70/30 EPDM/PP formulations (**left** column) and 30/70 EPDM/PP formulations (**right** column). From top to bottom: unvulcanized EPDM/PP blend, TAC(3), TAC(7), OV-POSS(3), and OV-POSS(7). Scale bars: 10 µm. Adapted from Ref. [13] under the Creative Commons Attribution 4.0 license; selected panels have been rearranged for comparison.

To provide morphological context for the crystallization results, selected SEM micrographs of the corresponding formulations from our previous study [13] are presented in Figure 10. The images illustrate differences in fracture-surface morphology between the unvulcanized blends and the coagent-containing TPVs. Together with the morphological analysis reported previously, these observations provide a basis for discussing how the dispersed-phase structure may influence crystallization initiation and subsequent crystal development. However, the SEM images do not directly establish changes in PP-chain mobility or crystal-growth rate.

As reported in our previous study [13], dynamic vulcanization of the 70/30 EPDM/PP system in the presence of peroxide and coagents induced phase inversion, transforming the initially continuous EPDM phase into fine crosslinked domains dispersed within the PP matrix. The same study also provides quantitative evidence of differences in network formation. For the 70/30 EPDM/PP formulations, the reported crosslink densities were 238.7 ± 17.2 and 245.1 ± 10.5 mol/m^3^ for TAC(3) and TAC(7), respectively, compared with 328.2 ± 25.6 and 511.6 ± 13.2 mol/m^3^ for OV-POSS(3) and OV-POSS(7). For the 30/70 formulations, the corresponding values were 505.2 ± 20.5 and 601.4 ± 13.2 mol/m^3^ for TAC(3) and TAC(7), respectively, and 748.9 ± 40.8 and 806.6 ± 50.6 mol/m^3^ for OV-POSS(3) and OV-POSS(7), respectively. The higher crosslink densities reported for the OV-POSS-containing formulations support the proposed contribution of network-related constraints to the crystallization response. Such constraints may influence the rearrangement of PP chains during subsequent crystal development. Alongside the previously reported changes in phase morphology, possible peroxide-induced PP-chain scission may also contribute to the observed crystallization behavior. The refined morphology, particularly in the presence of OV-POSS, may provide additional potential sites for heterogeneous nucleation, consistent with the higher crystallization onset temperatures. The crosslinked EPDM domains and the higher network densities reported previously may constrain PP-chain rearrangement and subsequent lamellar and spherulitic development. In addition, the development of crystals at numerous nucleation sites may promote earlier crystal impingement. These competing effects provide a possible explanation for the generally longer Avrami-derived *t_0.5_* values of the coagent-containing 70/30 TPVs relative to the corresponding unvulcanized blend. However, the cooling-rate-dependent response to OV-POSS loading indicates that crosslink density alone cannot account for the observed half-times. The results therefore suggest that facilitated crystallization initiation can coexist with a longer time to reach 50% relative crystallinity. This kinetic behavior is not inconsistent with the increased PP-normalized crystallinity observed for OV-POSS-containing TPVs, since the Avrami-derived half-time reflects the time required to reach 50% of the relative crystallization conversion rather than the final degree of crystallinity.

Overall, the Avrami-derived *t_0.5_* values indicate that higher crystallization onset temperatures do not necessarily correspond to shorter crystallization half-times. The effects of coagent incorporation depended on formulation and cooling rate, while the fitted parameters alone do not distinguish nucleation from crystal growth.

Both the Avrami-derived *t_0.5_* and experimental DSC t*_1/2_* values decreased with increasing cooling rate. The experimental *t_1/2_* was determined directly from the relative crystallinity-time curves, whereas *t_0.5_* was calculated using parameters fitted over 20–80% relative crystallinity. Thus, the model-derived half-time was evaluated within the fitted conversion range. For example, neat PP at 5 °C/min exhibited a calculated *t_0.5_* of 2.64 min, close to the experimental *t_1/2_* of 2.60 min. This agreement supports the description of conversion near its midpoint for this condition, without independently validating a specific crystallization mechanism.

Another widely used approach for analyzing non-isothermal crystallization kinetics is the Ozawa model [31]. Unlike the Avrami model, Ozawa considered the effect of the cooling rate and modified the Avrami approach by assuming that the sample is cooled at a constant rate within a very short period and that the non-isothermal crystallization process can be divided into an infinite number of short time intervals. Therefore, the time variable used in the Avrami model is replaced by the cooling rate in the Ozawa model. The relative crystallinity can be expressed according to Equation (8):

(8) $$1-X(T)=exp(\frac{-K\left(T\right)}{\beta^{m}})$$

where K(T) is the temperature-dependent Ozawa cooling function and m is the Ozawa exponent associated with the assumed nucleation and crystal-growth behavior. Taking the logarithm of Equation (8) gives:

(9) $$\ln\left[-\ln\left(1-X\left(T\right)\right)\right]=\ln K\left(T\right)-mln\beta$$

where β denotes the magnitude of the cooling rate. At a fixed temperature, plotting ln[−ln(1 − X(T))] against ln β gives a slope of −m and an intercept of lnK(T). Thus, m is obtained from the negative slope, while K(T) is calculated by exponentiating the intercept. Reliable determination of these parameters requires sufficient valid data and an adequately linear relationship (Equation (9)).

**Figure 11:**
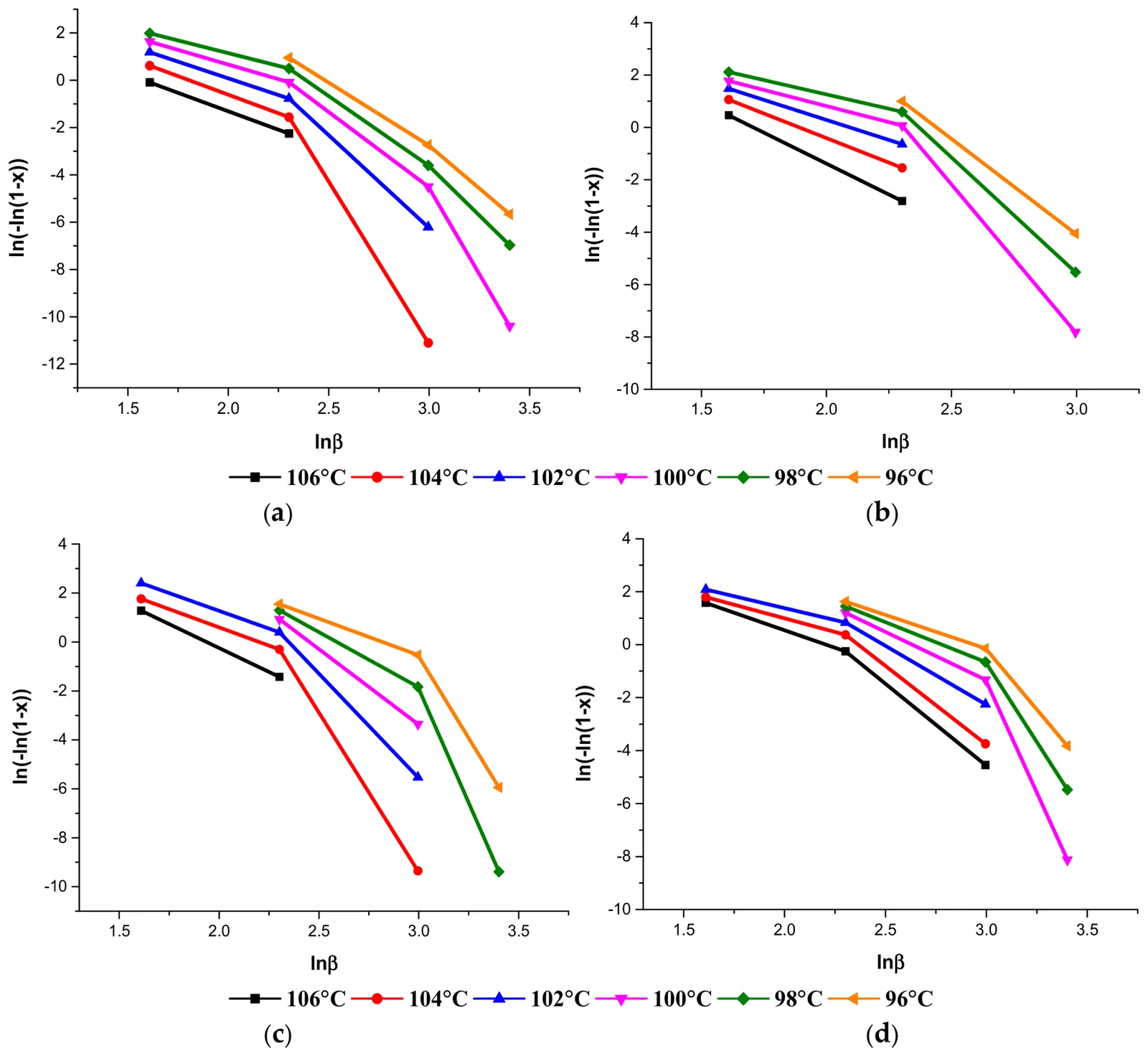

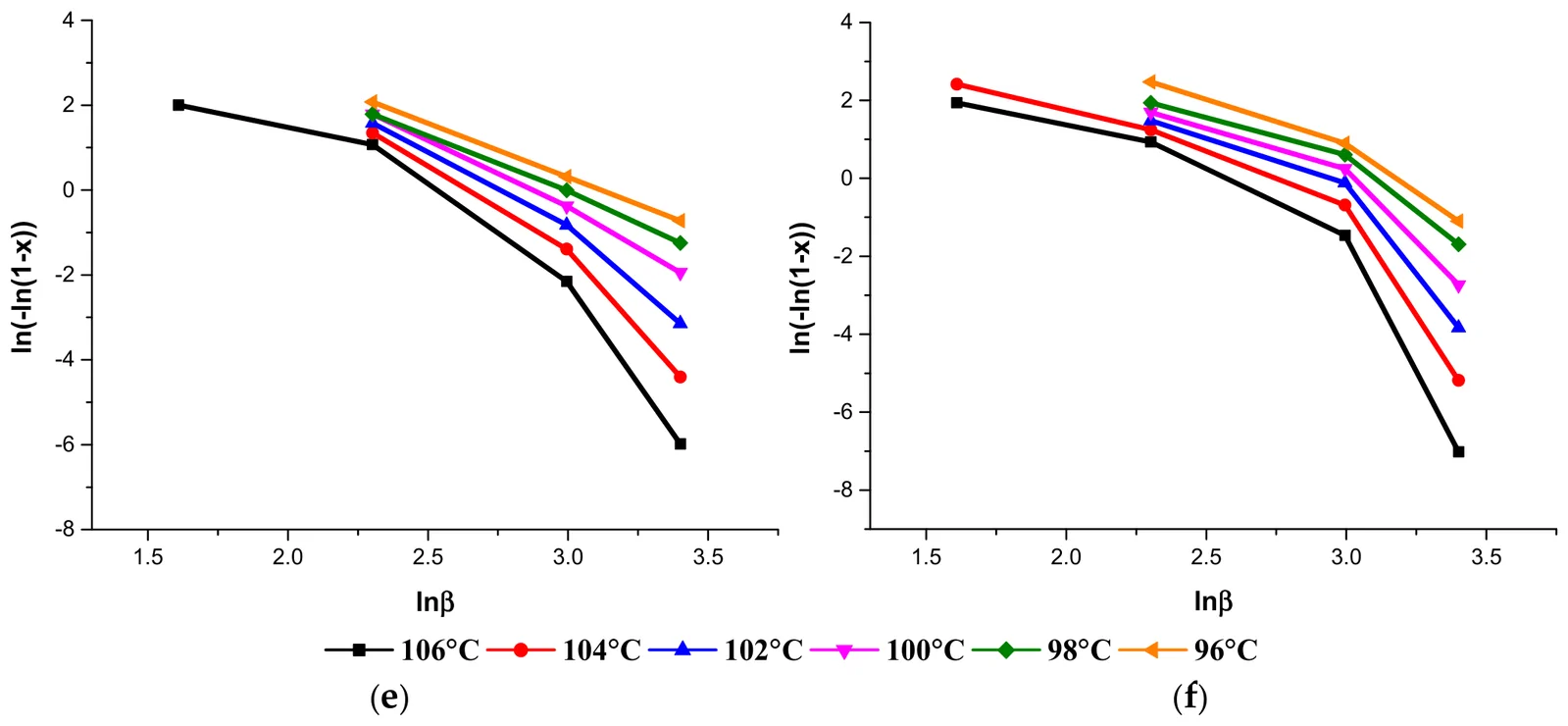
Ozawa plots of 30/70 EPDM/PP TPVs: (**a**) PP, (**b**) EPDM/PP, (**c**) EPDM/PP/TAC(3), (**d**) EPDM/PP/TAC(7), (**e**) EPDM/PP/OV-POSS(3), and (**f**) EPDM/PP/OV-POSS(7).

**Figure 12:**
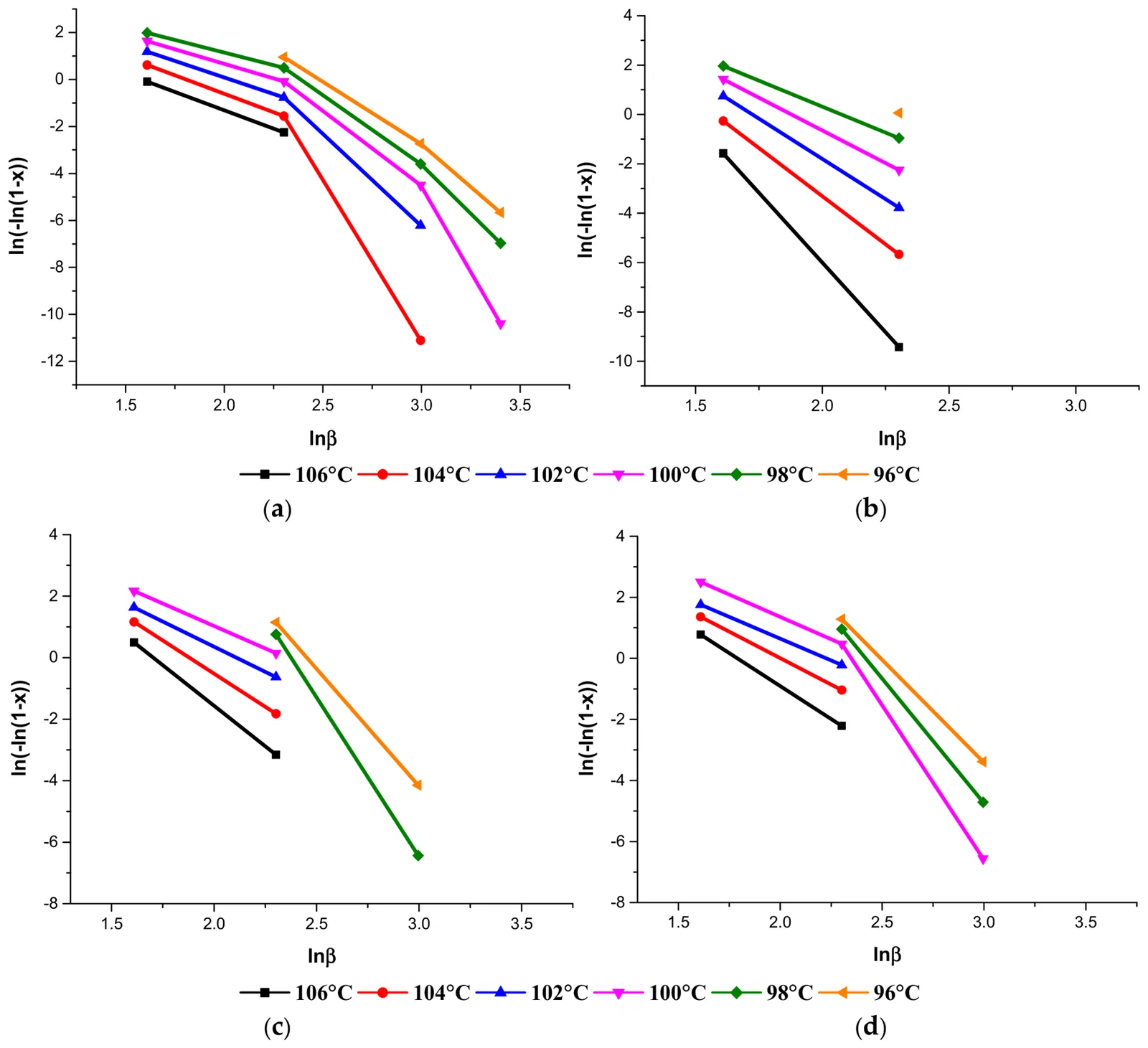

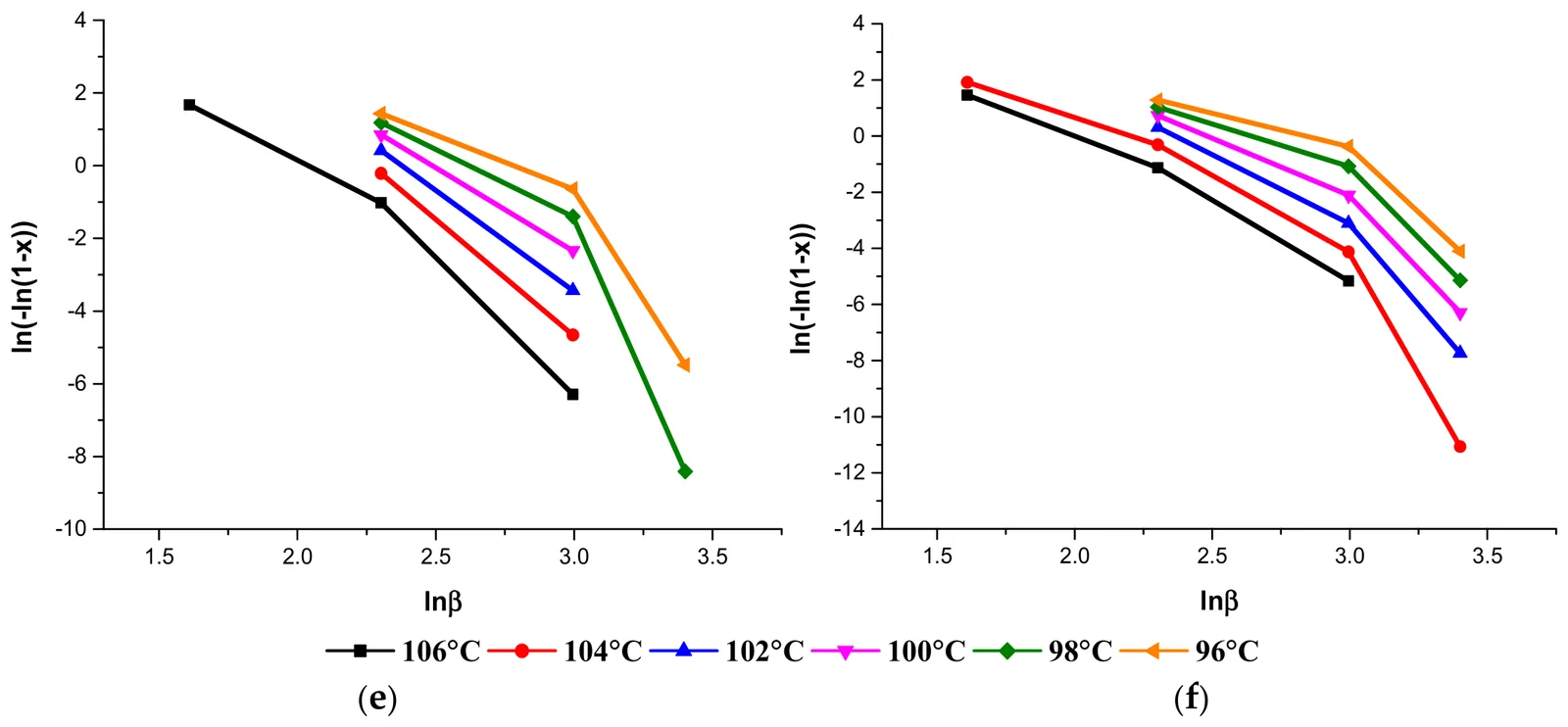
Ozawa plots of 70/30 EPDM/PP TPVs: (**a**) PP, (**b**) EPDM/PP, (**c**) EPDM/PP/TAC(3), (**d**) EPDM/PP/TAC(7), (**e**) EPDM/PP/OV-POSS(3), and (**f**) EPDM/PP/OV-POSS(7).

The Ozawa plots for the investigated samples are presented in Figure 11 and Figure 12. The analysis compares ln[−ln(1 − X(T))] with ln β at fixed temperatures. Six temperatures were initially selected for each EPDM/PP composition. However, the cooling-rate-dependent shifts in the crystallization intervals limited the availability of suitable relative crystallinity values at common temperatures. At some selected temperatures, certain samples had not yet started crystallizing or had already essentially completed crystallization. Values of X(T) approaching zero or unity cannot provide a reliable logarithmic transformation because the transformed values become undefined at the endpoints and highly sensitive to experimental uncertainty near them. Consequently, not all selected temperatures yielded valid data points at all four cooling rates, accounting for the apparent incompleteness of the plots.

Limitations of the Ozawa analysis have also been discussed in the literature [10,32,33]. In addition to the limited overlap of the crystallization intervals, deviations from the model assumptions may affect the analysis. For example, secondary crystallization, which is not explicitly accounted for in the conventional Ozawa formulation, has been discussed as a possible contributor to deviations from linearity [32]. Furthermore, cooling-rate-dependent changes in nucleation and growth behavior may limit the description of crystallization using a single Ozawa exponent at a given temperature. These effects may contribute to the difficulties encountered in analyzing the present multiphase systems; however, their individual contributions cannot be established from the Ozawa plots alone. Therefore, insufficient data coverage should be distinguished from poor linearity. The available data did not permit reliable determination and comparison of the Ozawa parameters across the investigated formulations.

Mo et al. [34] proposed a new method for analyzing the non-isothermal crystallization behavior of polymers by combining the Avrami and Ozawa models, as expressed in Equations (10) and (11):

(10) $$\ln Z+n\ln t=\ln K\left(T\right)-mln\beta$$

(11) $$\ln\beta =\ln F\left(T\right)-\alpha lnt$$

In the combined Avrami-Ozawa approach, F(T) represents the cooling rate required to reach a selected relative crystallinity in unit time. At the same relative crystallinity and using consistent units, a lower F(T) indicates that the specified conversion can be achieved at a lower cooling rate within unit time. The parameter α = n/m is the ratio of the Avrami exponent n to the Ozawa exponent m and describes the dependence of the required cooling rate on crystallization time within the combined formulation. Both exponents reflect nucleation and crystal-growth behavior within their respective model assumptions; therefore, α should not be interpreted as a direct measure of crystal-growth dimensionality. Neither F(T) nor α independently separates nucleation from crystal growth. For a plot of ln β against ln t, the slope is -α and the intercept is ln F(T); therefore, F(T) is obtained by exponentiating the intercept.

**Figure 13:**
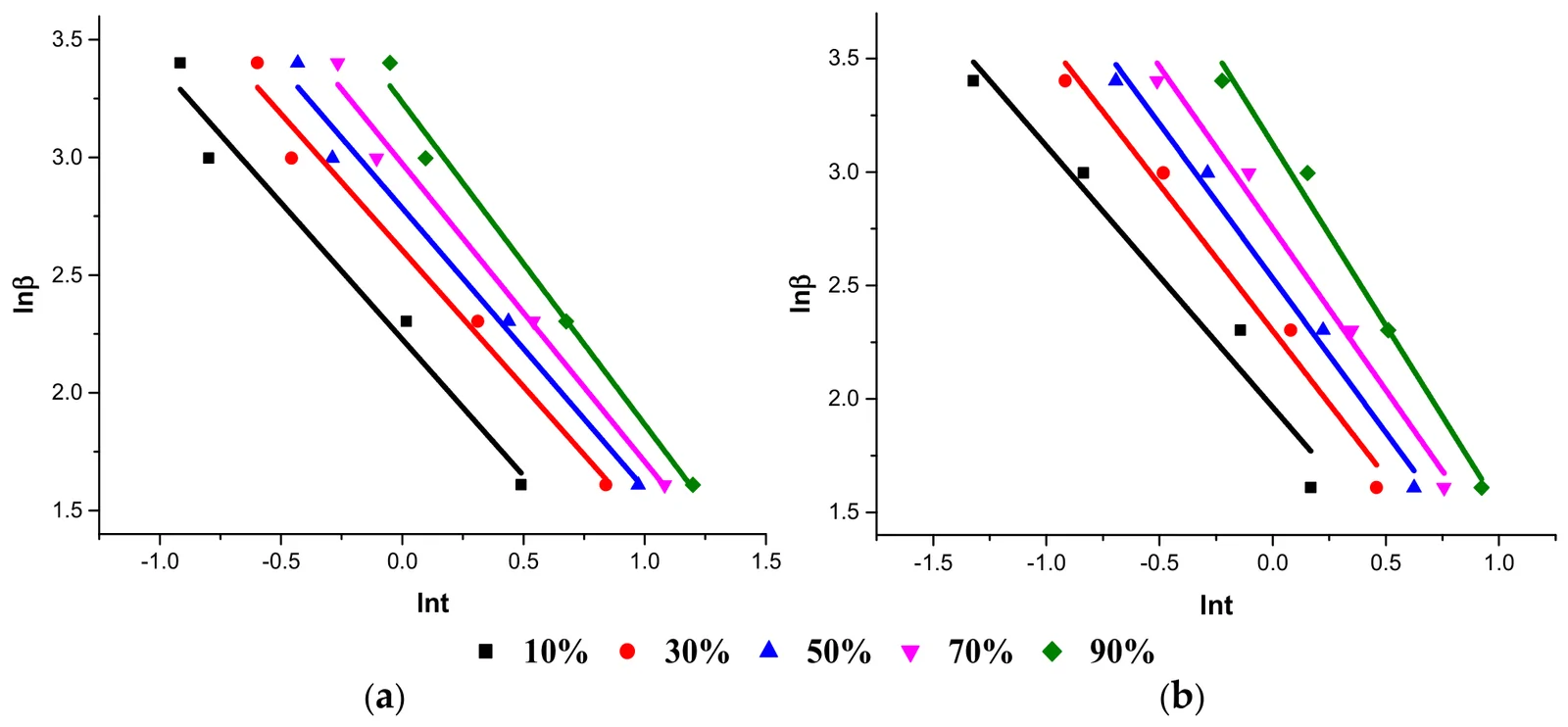

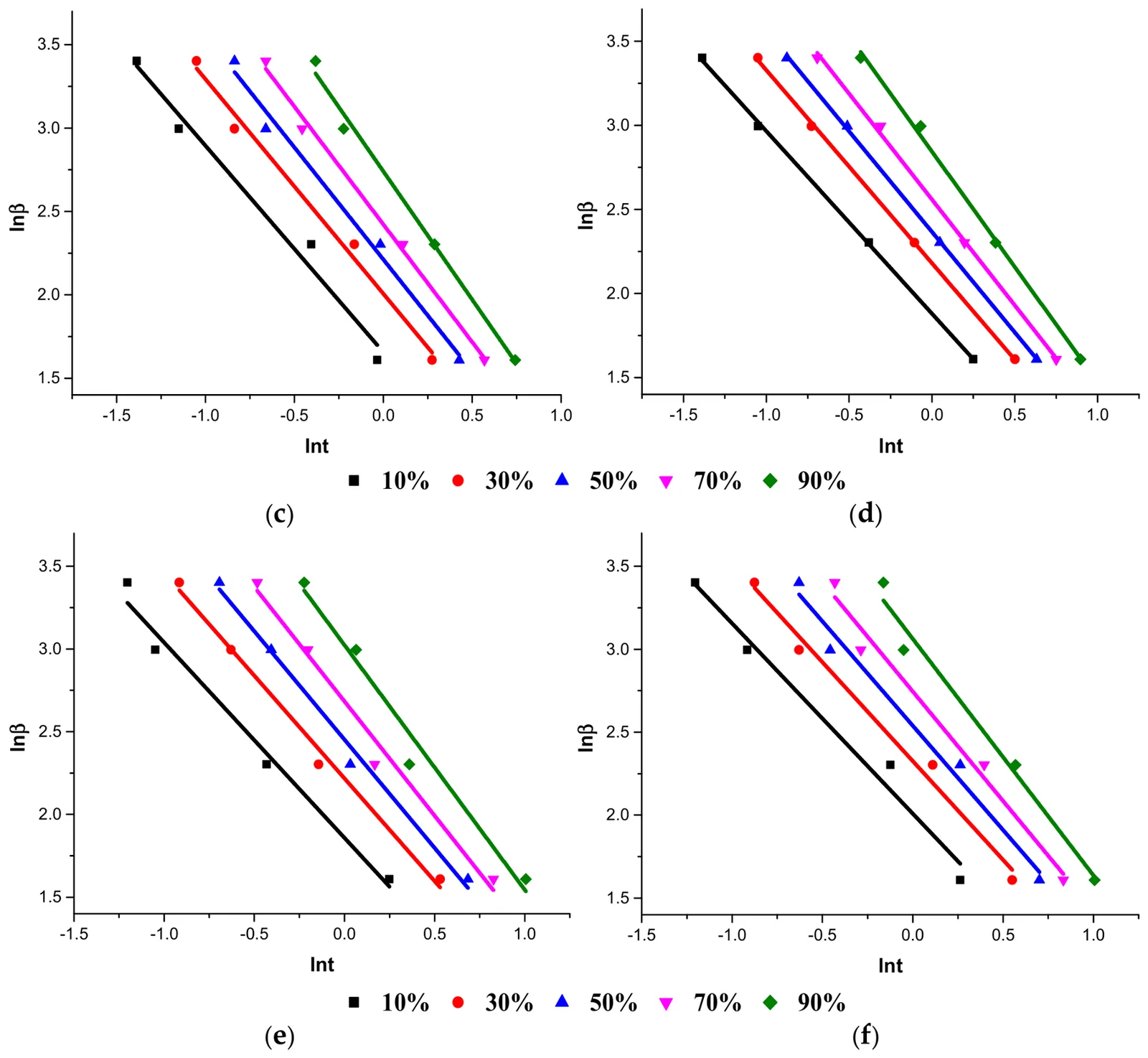
Combined Avrami-Ozawa plots of 30/70 EPDM/PP TPVs: (**a**) PP, (**b**) EPDM/PP, (**c**) EPDM/PP/TAC(3), (**d**) EPDM/PP/TAC(7), (**e**) EPDM/PP/OV-POSS(3), and (**f**) EPDM/PP/OV-POSS(7).

**Figure 14:**
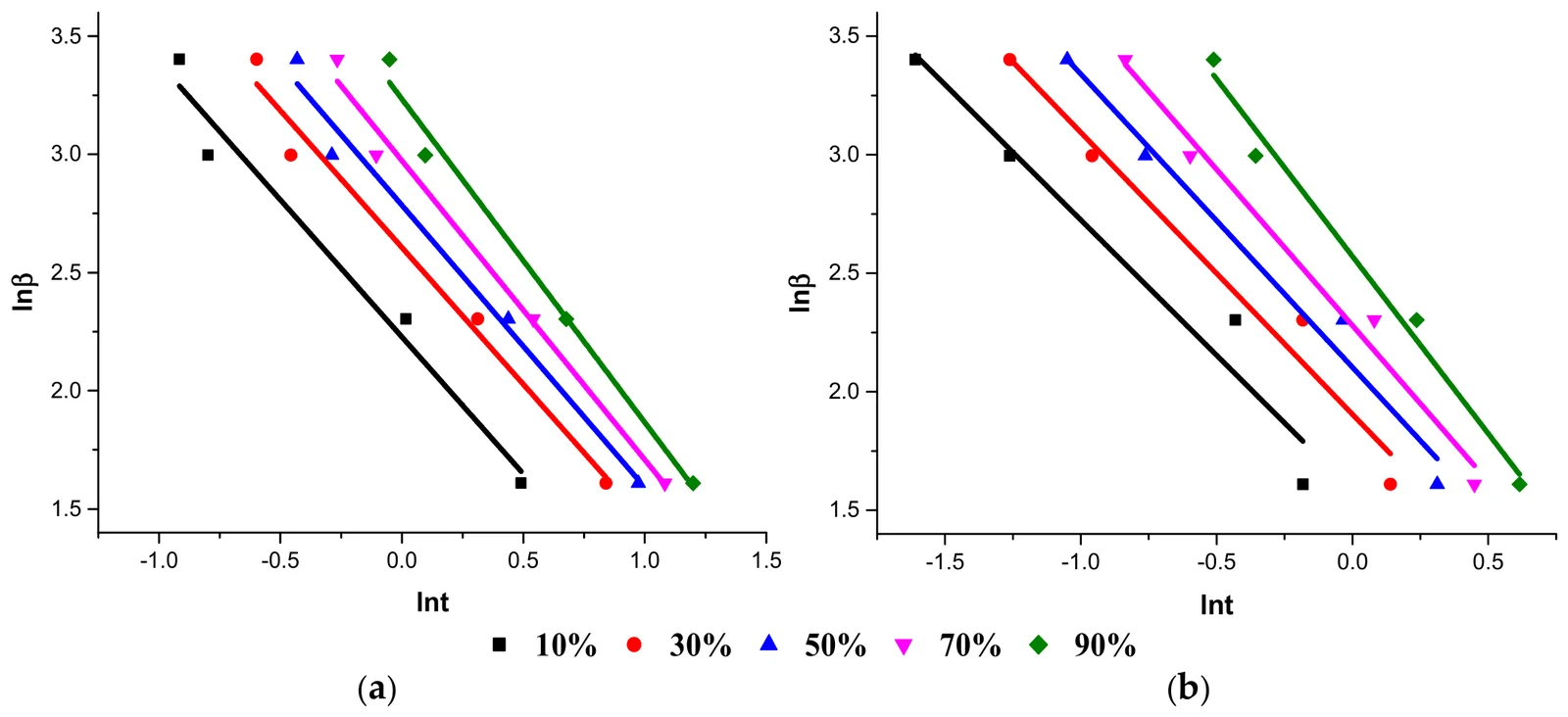

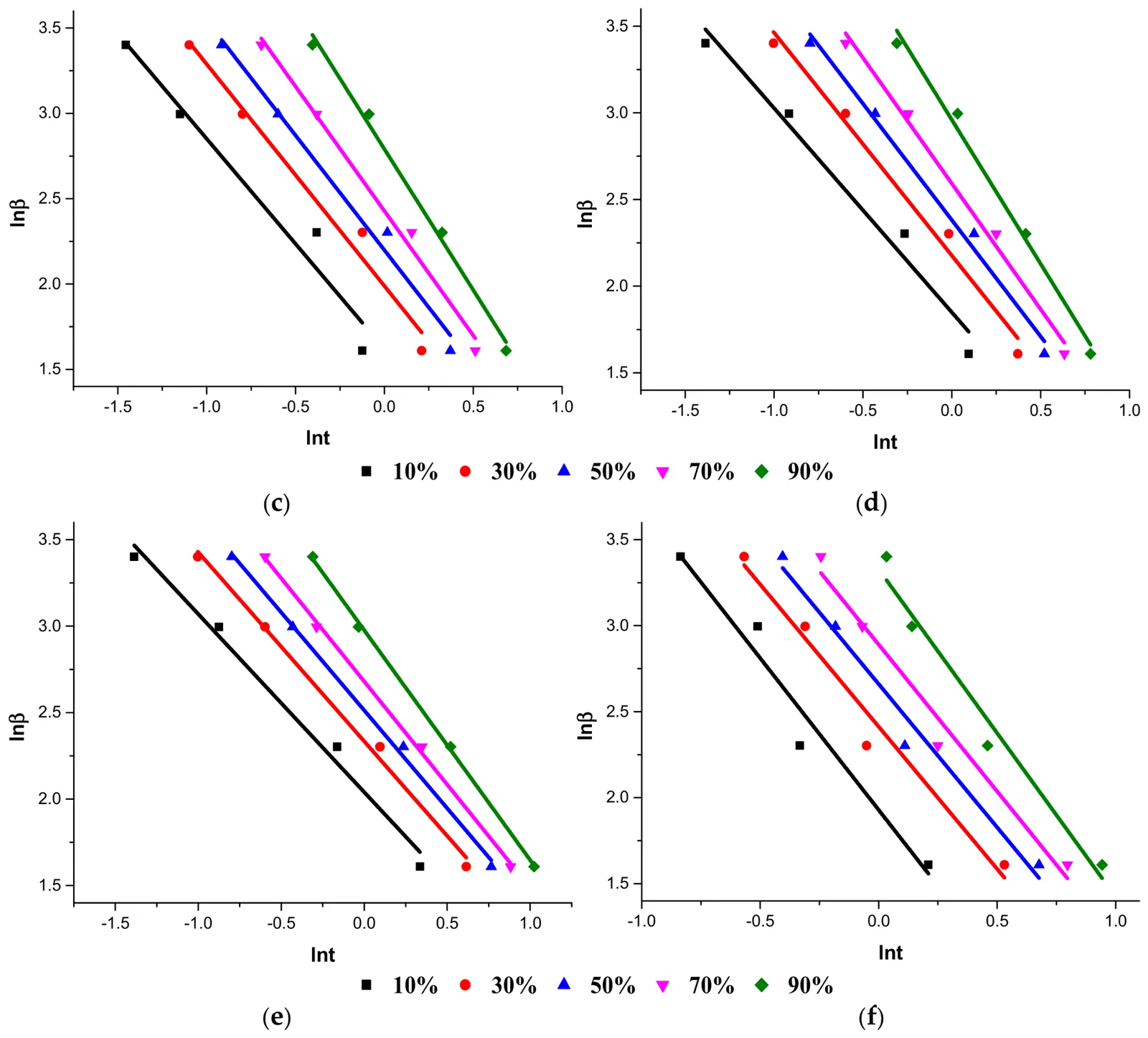
Combined Avrami-Ozawa plots of 70/30 EPDM/PP TPVs: (**a**) PP, (**b**) EPDM/PP, (**c**) EPDM/PP/TAC(3), (**d**) EPDM/PP/TAC(7), (**e**) EPDM/PP/OV-POSS(3), and (**f**) EPDM/PP/OV-POSS(7).

The plots obtained at different degrees of relative crystallinity for neat PP, 30/70 and 70/30 EPDM/PP blends, and the corresponding TPVs are presented in Figure 13 and Figure 14, respectively. The kinetic parameters calculated from these plots are summarized in Table 7 and Table 8.

The high R^2^ values indicate good linear correlations over the investigated range, although they do not independently validate a specific crystallization mechanism. As shown in Table 7 and Table 8, F(T) values increased with relative crystallinity for all formulations, indicating that reaching a higher conversion within unit time requires a higher cooling rate within the combined model. Comparisons between formulations should therefore be made at the same relative crystallinity, where a lower F(T) indicates a lower cooling rate required to achieve the selected conversion within unit time.

The F(T) values of neat PP ranged from 9.28 to 25.37 as relative crystallinity increased from 10% to 90%. The corresponding ranges were 7.13–22.72 for the 30/70 EPDM/PP blend and 4.87–13.08 for the 70/30 EPDM/PP blend (Table 7 and Table 8). At each investigated relative crystallinity, both blends exhibited lower F(T) values than neat PP, with the lowest values obtained for the EPDM-rich blend. Within the combined model, these results indicate that the blends required lower cooling rates to reach the selected conversion within unit time. This composition-dependent response characterizes the overall conversion kinetics and should not, by itself, be interpreted as evidence that EPDM enhances nucleation or restricts crystal growth.

For the 30/70 EPDM/PP formulations, TAC(3) and OV-POSS(3) exhibited lower F(T) values than both neat PP and the corresponding blend at all investigated relative crystallinities. Increasing the coagent-to-DCP mass ratio from 3 to 7 increased F(T) for both coagents. Nevertheless, TAC(7) retained lower F(T) values than the unvulcanized blend throughout the investigated range, whereas OV-POSS(7) exhibited values close to those of the blend. Thus, the higher coagent loading increased the cooling rate required to achieve a selected conversion within unit time relative to the lower-loading formulations, but did not uniformly retard crystallization relative to the blend.

For the 70/30 EPDM/PP formulations, all coagent-containing TPVs exhibited higher F(T) values than the corresponding blend at matched relative crystallinities. Within the combined model, these formulations therefore required higher cooling rates to reach the selected conversion within unit time. Most also exhibited lower F(T) values than neat PP; however, OV-POSS(7) exceeded neat PP at 90% relative crystallinity, with values of 27.85 and 25.37, respectively. These results indicate that the effects of coagent incorporation depended on blend composition, loading, and conversion level. The finer EPDM dispersion and differences in network formation reported previously [13] provide possible context for competing contributions from crystallization initiation and subsequent crystal development, but the fitted parameters alone do not establish their underlying mechanism.

At 50% relative crystallinity, α ranged from 1.20 to 1.36 in the 30/70 formulations and from 1.13 to 1.67 in the 70/30 formulations. The wider range in the EPDM-rich system indicates a more pronounced formulation-dependent variation in the cooling-rate-time relationship. In particular, the 70/30 OV-POSS(7) formulation exhibited higher α values than OV-POSS(3) at all investigated relative crystallinities. These differences characterize the empirical cooling-rate dependence and do not independently establish changes in nucleation mechanism or crystal-growth geometry.

It is well known that materials such as POSS can often act as effective nucleating agents in polymer systems [25,35]. To evaluate the nucleation activity of additives in polymers, Dobreva and Gutzow proposed a simple and widely used method [36,37]. The activation energy can be considered a measure of the reduction in the work required for three-dimensional nucleation in the presence of a foreign component. Typically, the nucleation activity parameter (Φ) ranges from 0 to 1. The lower the value of Φ, the higher the nucleation activity of the added component. The equation describing this approach is given in Equation (12):

(12) $$\Phi \;=\frac{B^{*}}{B}$$

Here, B* and B represent the heterogeneous and homogeneous nucleation parameters, respectively. If the additive does not exhibit any nucleating activity, the value of Φ will be equal to 1. Conversely, as the nucleating efficiency of the additive increases, the Φ value approaches 0. Neat PP was selected as the reference for assessing the effect of EPDM incorporation. For the coagent-containing TPVs, the corresponding unvulcanized EPDM/PP blend was used as the reference to assess changes at a fixed blend composition. Thus, the reported nucleation activity values describe changes relative to the specified reference material. Values calculated using different references should not be compared directly; comparisons among formulations should be made using a common reference. In addition, the B parameter can be calculated using Equation (13):

(13) $$B=\frac{{\omega \sigma }^{3}V_{m}^{2}}{3nk_{B}T_{m}^{0}{\Delta S}_{m}^{2}}$$

Here, ω is the geometric factor and σ is the specific surface energy. V_m_ denotes the molar volume of the crystallizing substance, n is the Avrami exponent, kᴮ is the Boltzmann constant, ΔS_m_ is the entropy of melting, and T_m_ is the equilibrium melting temperature. In addition, the B parameter can also be determined from experimental data using Equation (14).

(14) $$ln\beta =C-\frac{B}{{\Delta T}_{C}^{2}}$$

Here, β is the cooling rate, C is a constant, and ΔT_c_ is the difference between the melting temperature and the crystallization temperature (T_m_ − T_c_). By plotting lnβ versus 1/ΔTc^2^, the B and B* parameters can be determined. The B values for neat PP and the 30/70 and 70/30 EPDM/PP blends, and the B* values for the corresponding TPVs, are obtained from the slopes of the resulting linear plots.

**Figure 15:**
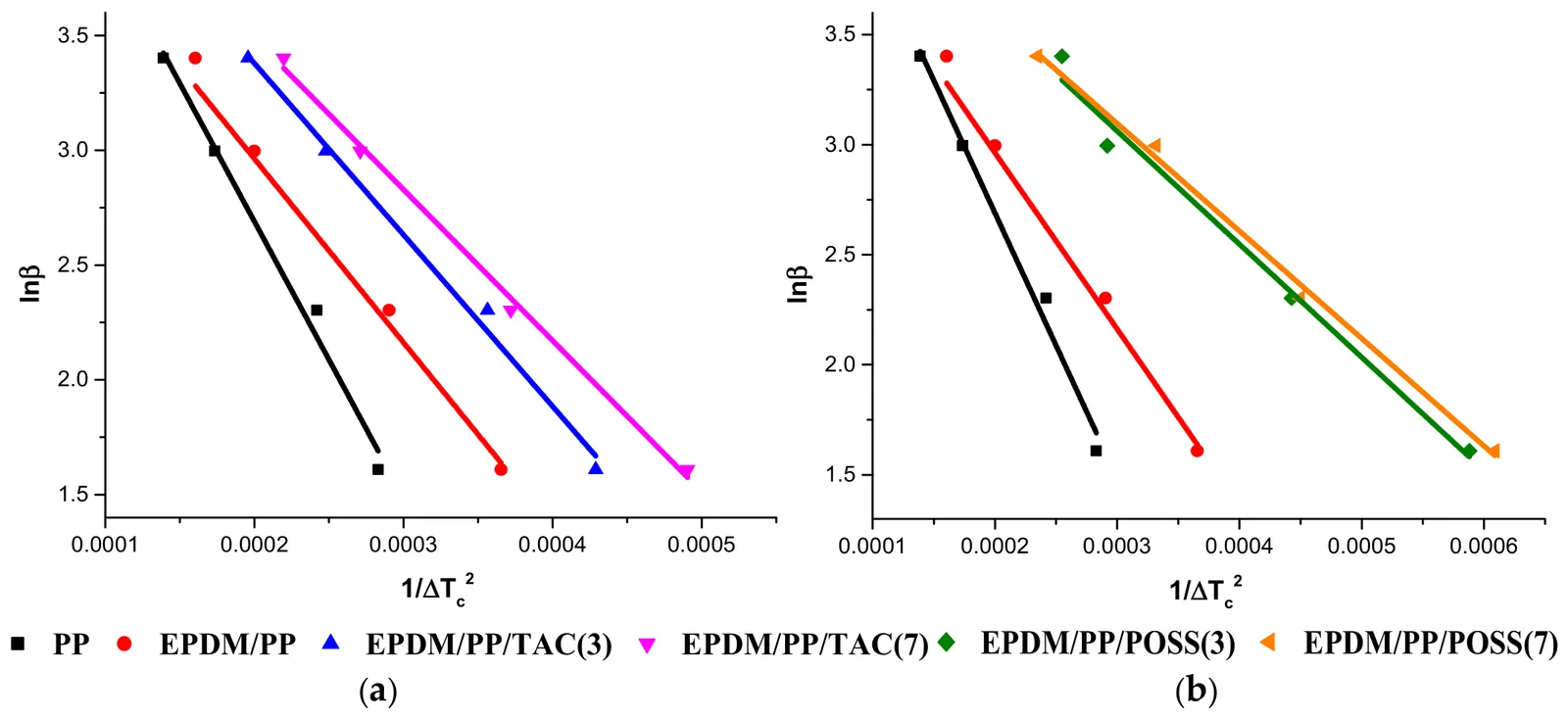
Nucleation activity plots of 30/70 EPDM/PP TPVs: (**a**) EPDM/PP/TAC and (**b**) EPDM/PP/OV-POSS.

**Figure 16:**
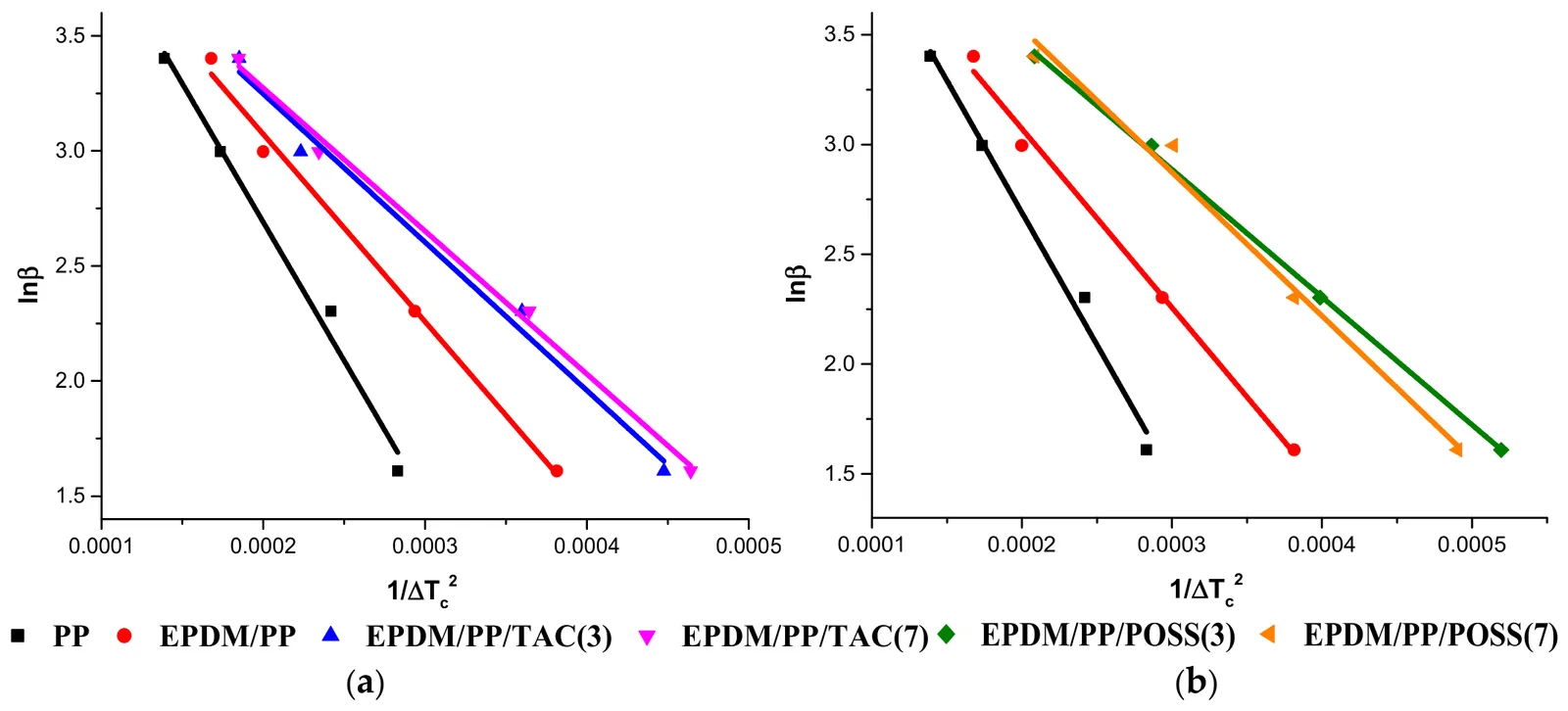
Nucleation activity plots of 70/30 EPDM/PP TPVs: (**a**) EPDM/PP/TAC and (**b**) EPDM/PP/OV-POSS.

The nucleation activity plots obtained for the neat components, blends, and TPVs prepared in this study are presented in Figure 15 and Figure 16, while the corresponding calculated data are summarized in Table 9 and Table 10.

For all coagent-containing formulations, the calculated Φ values were lower than unity, confirming that both TAC and OV-POSS contributed to heterogeneous nucleation. However, the Φ values ranged from 0.57 to 0.87, demonstrating that the magnitude of the nucleating effect depended on the EPDM/PP ratio, coagent type, and coagent loading.

For the 30/70 EPDM/PP formulations, the unvulcanized blend exhibited a Φ value of approximately 0.71 relative to neat PP. Using the corresponding blend as the reference, OV-POSS(3) and OV-POSS(7) exhibited Φ values of 0.60 and 0.57, respectively, whereas TAC(3) and TAC(7) exhibited values of 0.87 and 0.77 (Table 9). The blend-referenced TPV values should not be compared directly with the PP-referenced blend value of 0.71. All coagent-containing formulations exhibited values below unity, consistent with facilitated nucleation relative to the blend. On this common reference basis, the OV-POSS-containing formulations showed lower Φ values than their TAC-containing counterparts, with a slightly lower value at the higher OV-POSS loading.

For the 70/30 EPDM/PP formulations, the unvulcanized blend exhibited a Φ value of approximately 0.68 relative to neat PP. In contrast, the Φ values of 0.79, 0.76, 0.71, and 0.80 for TAC(3), TAC(7), OV-POSS(3), and OV-POSS(7), respectively, were calculated relative to the corresponding unvulcanized blend (Table 10).

These values likewise should not be compared directly with the blend value of 0.68. With the blend serving as the reference value of unity, all coagent-containing formulations showed apparent nucleating activity. Notably, OV-POSS(3) exhibited the lowest Φ value among these formulations, whereas increasing the OV-POSS-to-DCP mass ratio to 7 did not further improve this response. These findings are consistent with the higher crystallization onset temperatures observed by DSC, without necessarily implying faster overall crystallization. Relative to each composition’s corresponding blend, OV-POSS produced a larger reduction in the nucleation parameter in the PP-rich 30/70 system than in the EPDM-rich 70/30 system. This comparison describes the relative modification of each blend rather than an absolute ranking based on a common reference.

Kissinger [38] and Takhor [39] proposed different approaches for determining the crystallization activation energy (ΔE) by considering the influence of the cooling rate on the non-isothermal crystallization process. These methods are based on evaluating the variation in the crystallization temperature as a function of the cooling rate and are expressed by Equations (15) and (16), respectively:

(15) $$\frac{d\left[\ln\left(\frac{\beta }{T_{p}^{2}}\right)\right]}{d\left(\frac{1}{T_{p}}\right)}=-\frac{\Delta E}{R}$$

(16) $$\frac{d\left(ln\beta \right)}{d\left(\frac{1}{T_{p}}\right)}=-\frac{\Delta E}{R}$$

Here, T_p_ is the crystallization peak temperature, β is the cooling rate, and R is the universal gas constant.

**Figure 17:**
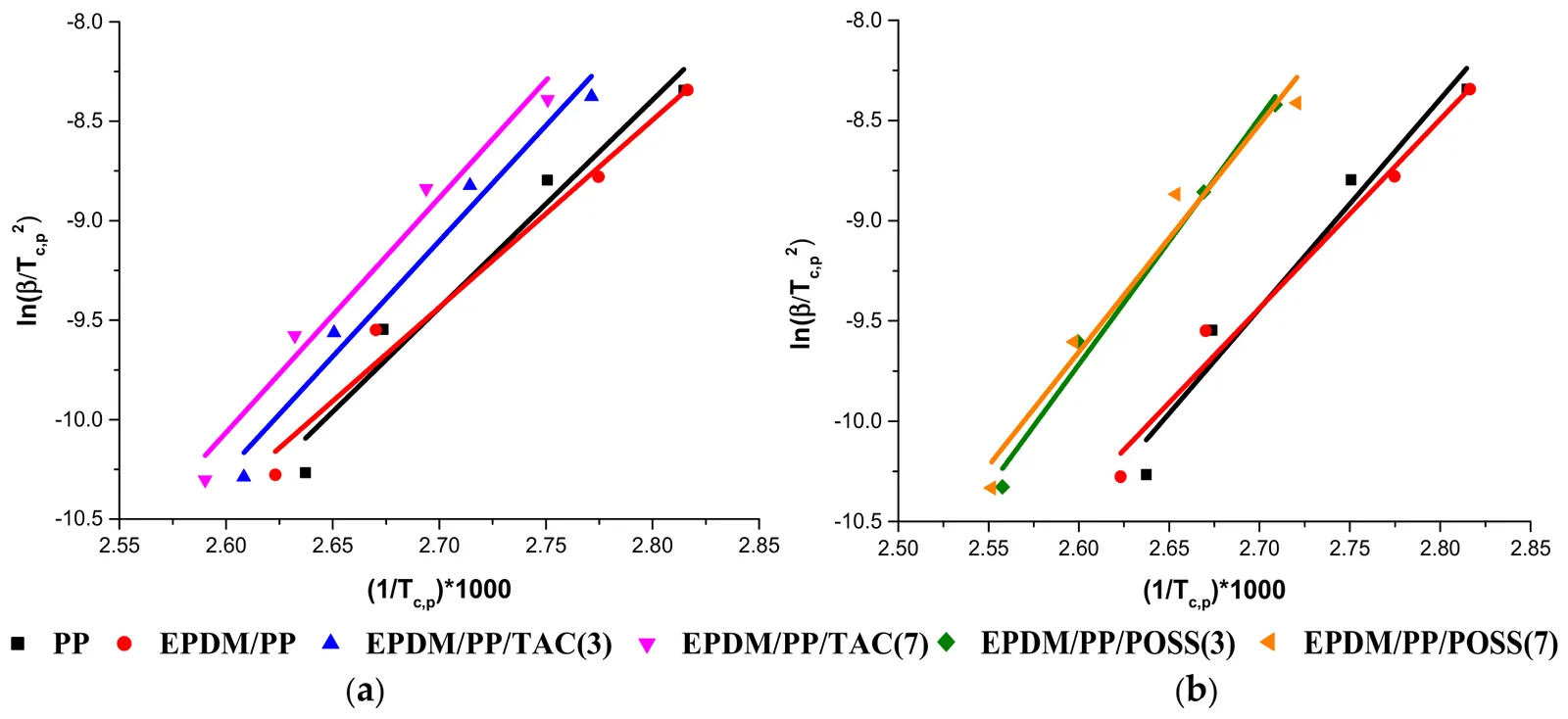
Kissinger plots for the 30/70 EPDM/PP TPVs: (**a**) EPDM/PP/TAC and (**b**) EPDM/PP/OV-POSS.

**Figure 18:**
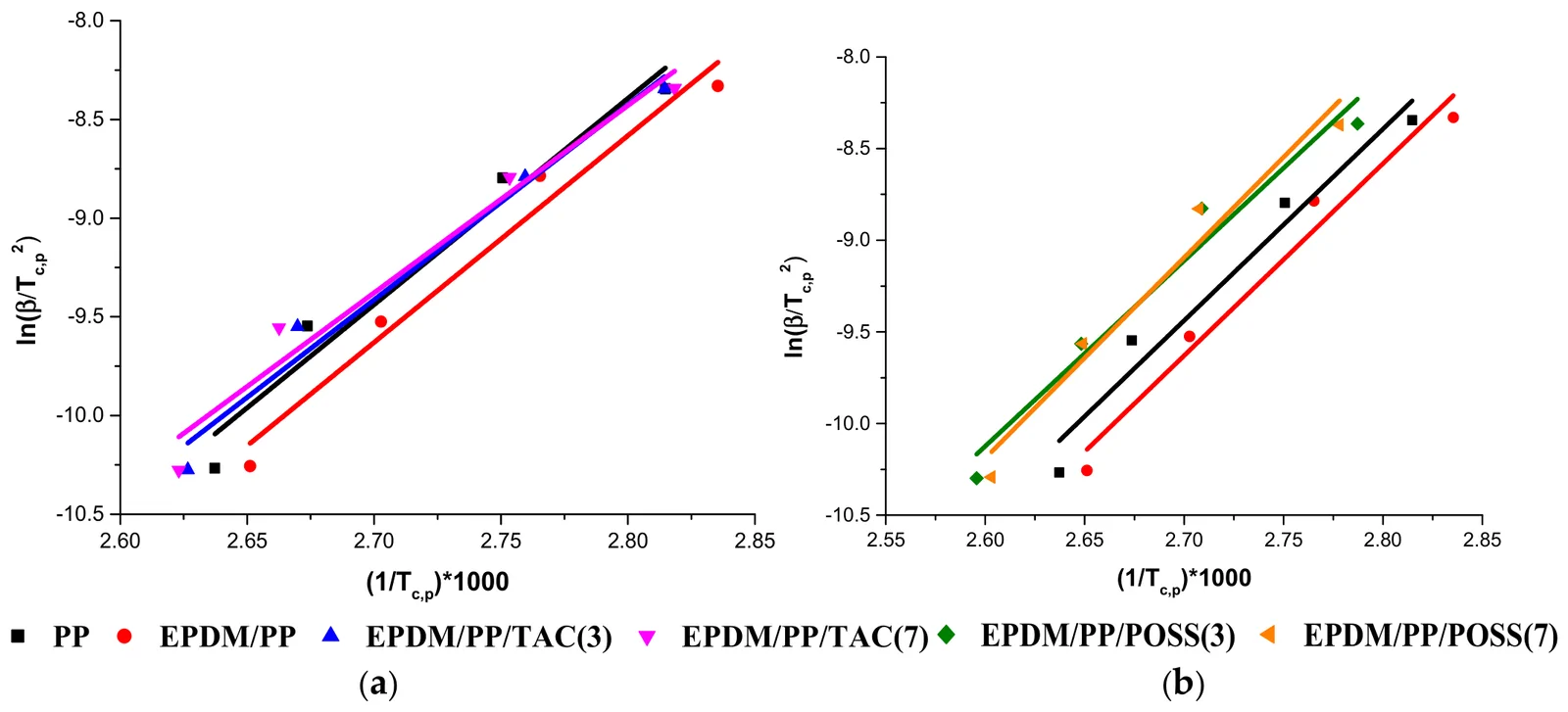
Kissinger plots for the 70/30 EPDM/PP TPVs: (**a**) EPDM/PP/TAC and (**b**) EPDM/PP/OV-POSS.

**Figure 19:**
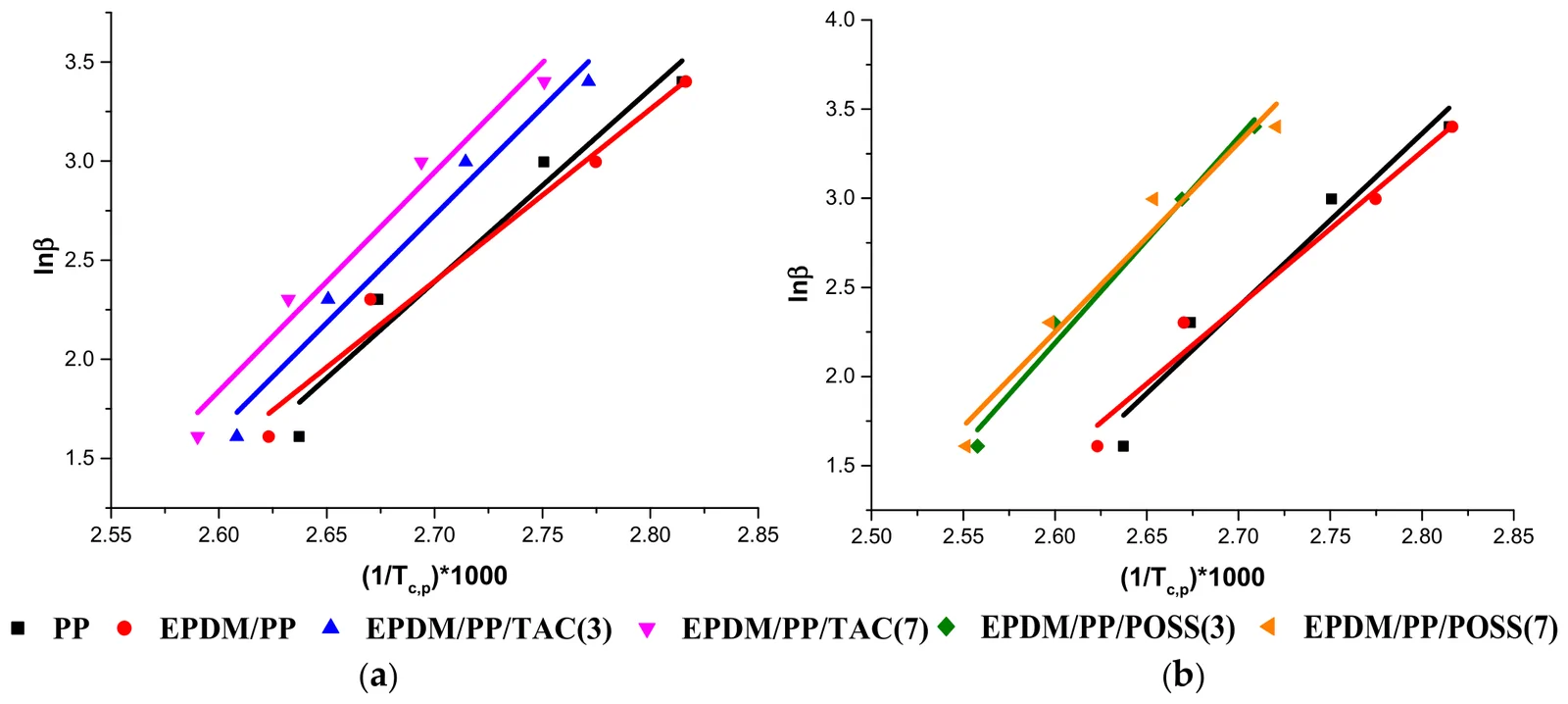
Takhor plots for the 30/70 EPDM/PP TPVs: (**a**) EPDM/PP/TAC and (**b**) EPDM/PP/OV-POSS.

**Figure 20:**
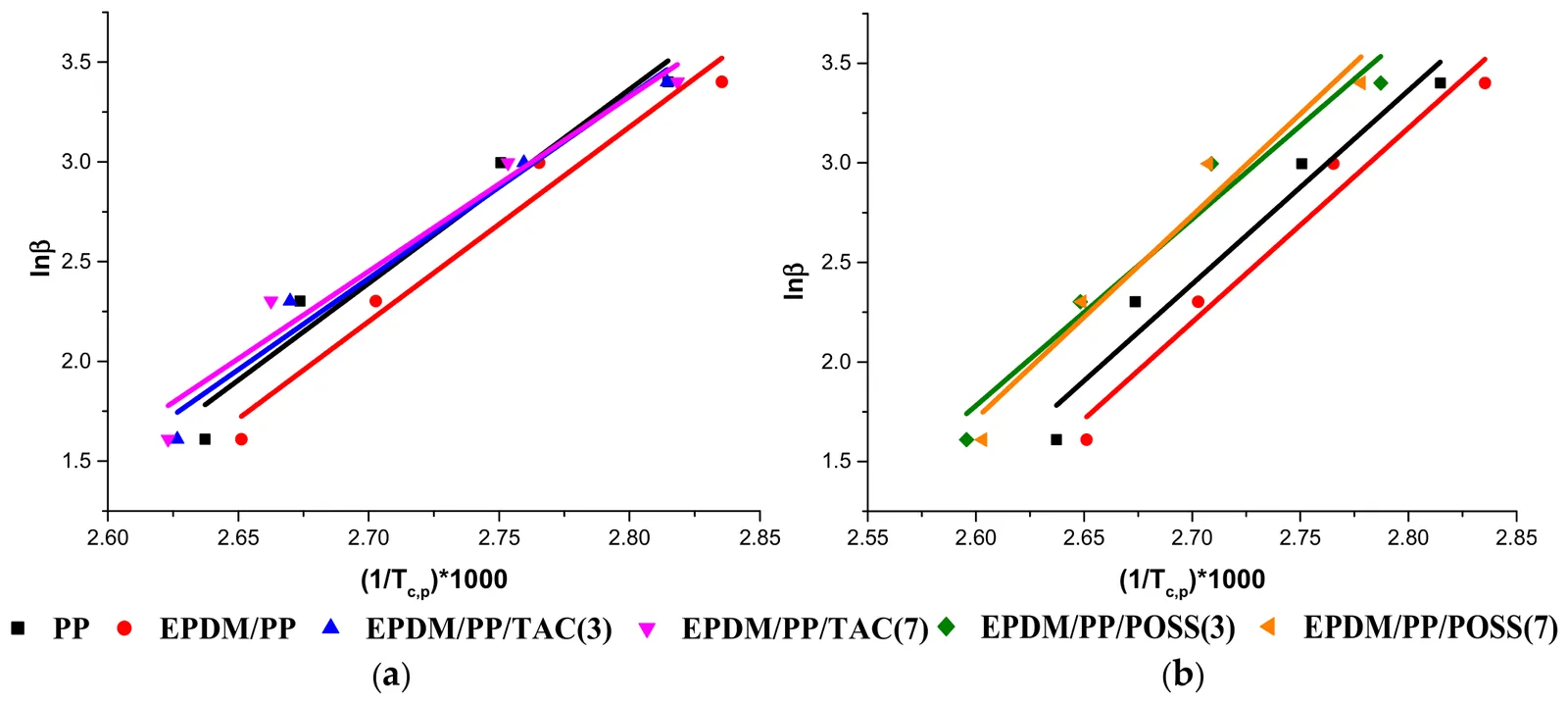
Takhor plots for the 70/30 EPDM/PP TPVs: (**a**) EPDM/PP/TAC and (**b**) EPDM/PP/OV-POSS.

The Kissinger and Takhor plots used to determine the activation energies of neat PP, 30/70 and 70/30 EPDM/PP blends, and the corresponding TPVs are presented in Figure 17, Figure 18, Figure 19 and Figure 20, respectively, while the calculated activation energy values are listed in Table 9 and Table 10.

According to the Kissinger model, a plot of ln(β/T_p_^2^) versus 1/T_p_ is obtained from Equation (14), whereas according to the Takhor model, a plot of lnβ versus 1/T_p_ is constructed from Equation (15). In both approaches, the value of −ΔE/R is determined from the slope of the resulting linear relationship.

When the activation energy values obtained using the Kissinger and Takhor methods for the samples containing 30/70 EPDM/PP are compared, it can be seen that the activation energy of PP increases upon blending with 30 phr EPDM, with the highest activation energy being obtained for the 30/70 EPDM/PP blend (Table 9). In contrast, the activation energy values of the 30/70 EPDM/PP TPVs were generally lower. Overall, the TPVs prepared with OV-POSS exhibited lower activation energy values than those prepared with TAC. The reduction in the particle size of the dispersed and crosslinked EPDM phase in the presence of the coagents lowered the energy barrier required to initiate crystallization.

The crystallization activation energy is also considered an important indicator of the crystallization ability of polymers [25]. Higher activation energy values correspond to lower crystallization ability. For the 30/70 EPDM/PP formulations, the incorporation of TAC and OV-POSS generally produced more negative apparent activation-energy values than the corresponding unvulcanized blend. However, the magnitude of this effect depended on coagent type and loading.

When the activation energy values obtained from the Kissinger and Takhor methods for the samples containing 70/30 EPDM/PP are compared, no significant change in the activation energy of PP was observed upon blending with 70 phr EPDM (Table 10). In general, only the 70/30 EPDM/PP/OV-POSS TPV containing the higher OV-POSS loading exhibited a lower activation energy than the corresponding 70/30 EPDM/PP blend. This behavior is believed to be associated with the smaller particle size of the dispersed EPDM phase formed after dynamic vulcanization, particularly at an OV-POSS-to-DCP mass ratio of 7. As discussed previously, the finer dispersion of EPDM particles increases the number of potential nucleation sites and reduces the energy barrier required for crystallization. Consequently, the crystallization process can proceed more readily. Among the 70/30 EPDM/PP formulations, a clear reduction in the apparent activation-energy barrier relative to the unvulcanized blend was primarily observed for the OV-POSS(7) formulation.

Although the Kissinger equation is one of the most widely used methods for evaluating the crystallization activation energy, another commonly employed approach for determining activation energy was developed by Friedman [40,41,42]. According to the Friedman method, different activation energy values can be calculated for each degree of crystallinity based on the following equation (Equation (17)):

(17) $$\ln\left(\frac{dX}{dt}\right)=C-\frac{\Delta E}{RT}$$

**Figure 21:**
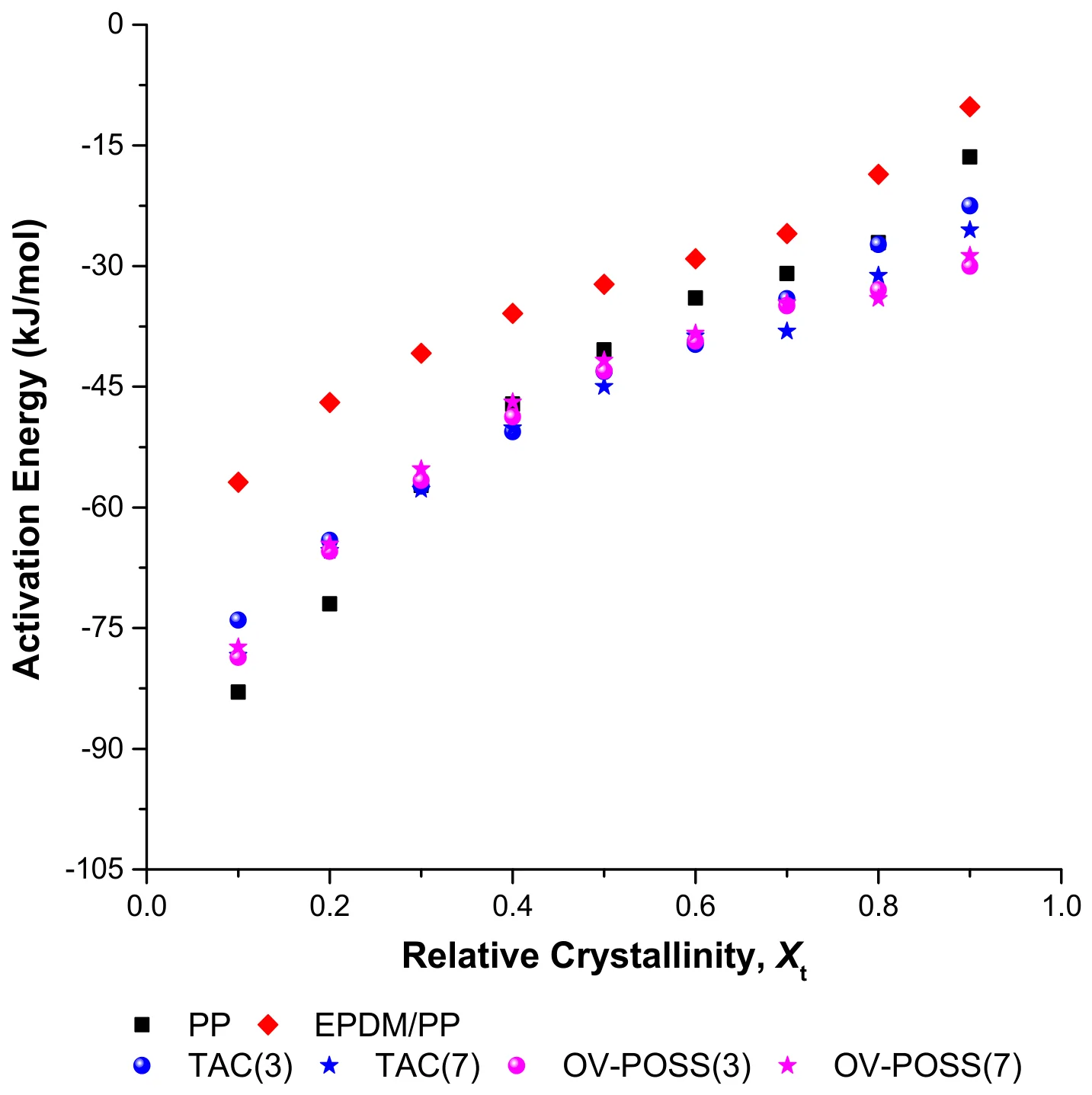
Activation energy versus relative crystallinity plots for 30/70 EPDM/PP TPVs.

**Figure 22:**
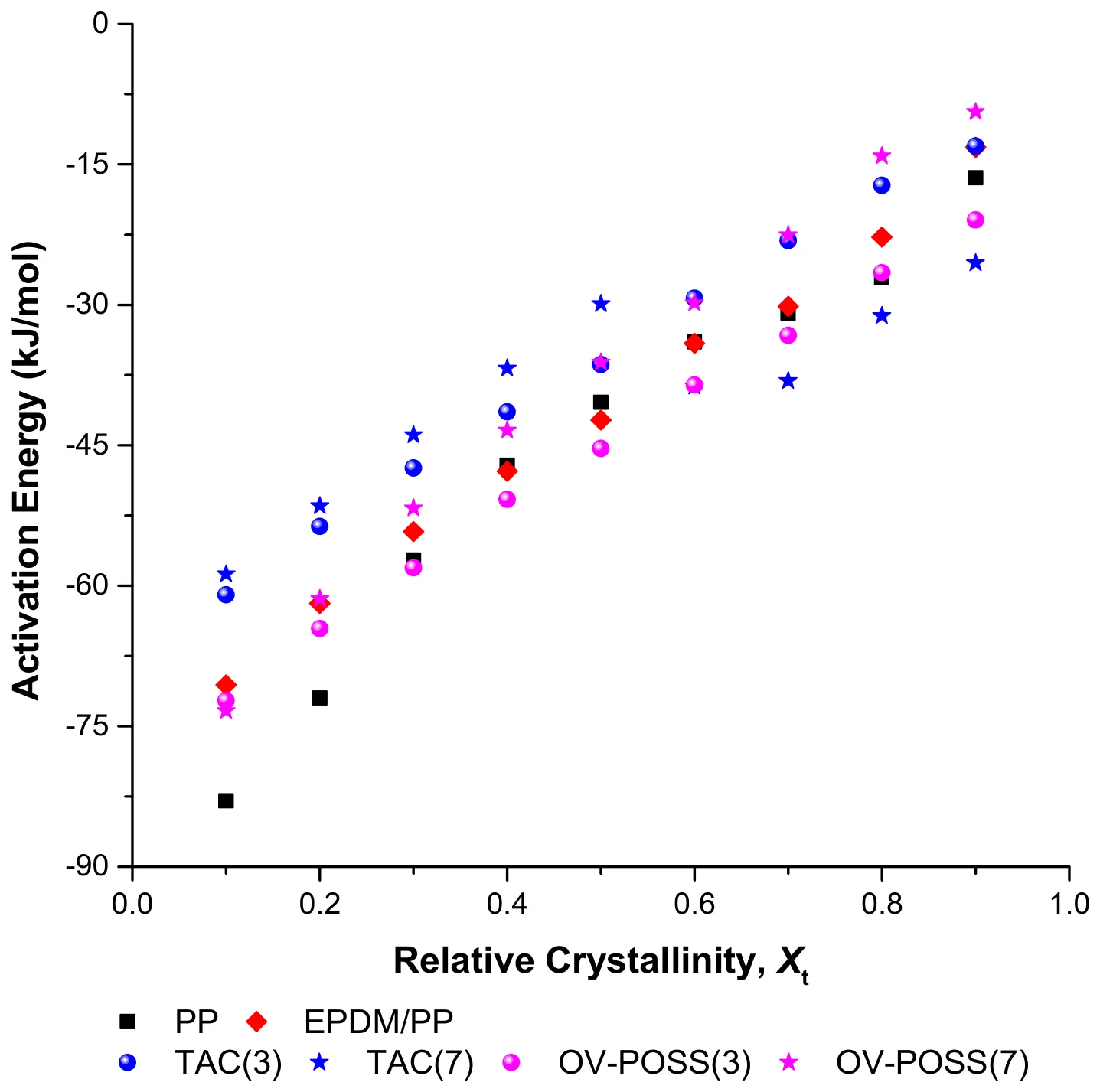
Activation energy versus relative crystallinity plots for 70/30 EPDM/PP TPVs.

Here, dX/dt represents the instantaneous crystallization rate as a function of time at a given relative crystallinity, X(t); ΔE is the activation energy corresponding to a specific X(t) value; R is the universal gas constant; and C is a constant. By plotting ln(dX/dt) versus 1/T at different cooling rates, the crystallization activation energy for the non-isothermal crystallization process can be determined from the slope of the resulting linear relationship. The activation energy versus relative crystallinity plots for neat PP, 30/70 and 70/30 EPDM/PP blends, and the corresponding TPVs are presented in Figure 21 and Figure 22.

The Friedman analysis shows that ΔE generally became less negative with increasing relative crystallinity. During the early stage (X_t_ ~ 0.1–0.3), the strongly negative values are consistent with crystallization being promoted by increasing supercooling. Both nucleation and the initial growth of crystalline entities contribute to the measured response. As conversion progresses through the intermediate stage (X_t_ ~ 0.3–0.7), continued crystal growth contributes to the development of crystallinity. At higher conversion (X_t_ ~ 0.7–0.9), crystal impingement, the decreasing availability of uncrystallized material, and possible constraints on chain rearrangement may increasingly influence crystallization. These contributions, together with the changing temperature, provide a possible explanation for the conversion dependence of ΔE. However, the Friedman plots alone do not establish distinct nucleation and growth dominated stages or confirm the occurrence of secondary crystallization.

For the 30/70 formulations, the coagent-containing TPVs exhibited more negative ΔE values than the corresponding unvulcanized blend throughout the investigated conversion range. Differences between TAC and OV-POSS varied with relative crystallinity, indicating that their effects cannot be represented by a single ranking over the entire crystallization process. In the 70/30 system, the relative positions of the formulations changed more noticeably with conversion. In particular, OV-POSS(7) exhibited more negative values than OV-POSS(3) at low conversion but less negative values at higher conversion. Considered alongside the DSC results, these observations are compatible with facilitated crystallization initiation and a loading-dependent response during subsequent crystal development. The higher crosslink density previously reported for the 70/30 OV-POSS(7) formulation provides a possible context for network-related constraints at later stages, although such constraints cannot be established from the Friedman results alone.

At a given relative crystallinity, the activation energies of different formulations are evaluated over different temperature ranges. Consequently, part of the variation in ΔE may reflect shifts in the crystallization temperature range rather than changes in the underlying mechanism. More negative ΔE values should therefore not be interpreted directly as evidence of faster overall crystallization or a lower nucleation barrier. Furthermore, because each value was determined using four cooling rates, small differences between formulations should be interpreted cautiously. The Friedman analysis thus complements the Kissinger and Takhor results by revealing conversion-dependent changes in the apparent temperature sensitivity of crystallization.

### 3.2. Polarized Optical Microscopy (POM) Results

Polarized optical microscopy (POM) analyses were performed to investigate the crystal morphology of neat PP, 30/70 and 70/30 EPDM/PP blends, and EPDM/PP/coagent TPVs. The crystallization behavior of the samples was examined by first heating them to 220 °C, holding them at this temperature for 5 min to erase their previous thermal history, and subsequently cooling them to 115 °C at a cooling rate of 5 °C/min. Based on the DSC results, representative images extracted from the recorded videos at selected temperatures between 135 and 115 °C were used to compare the development of crystalline morphology among the samples.

**Figure 23:**
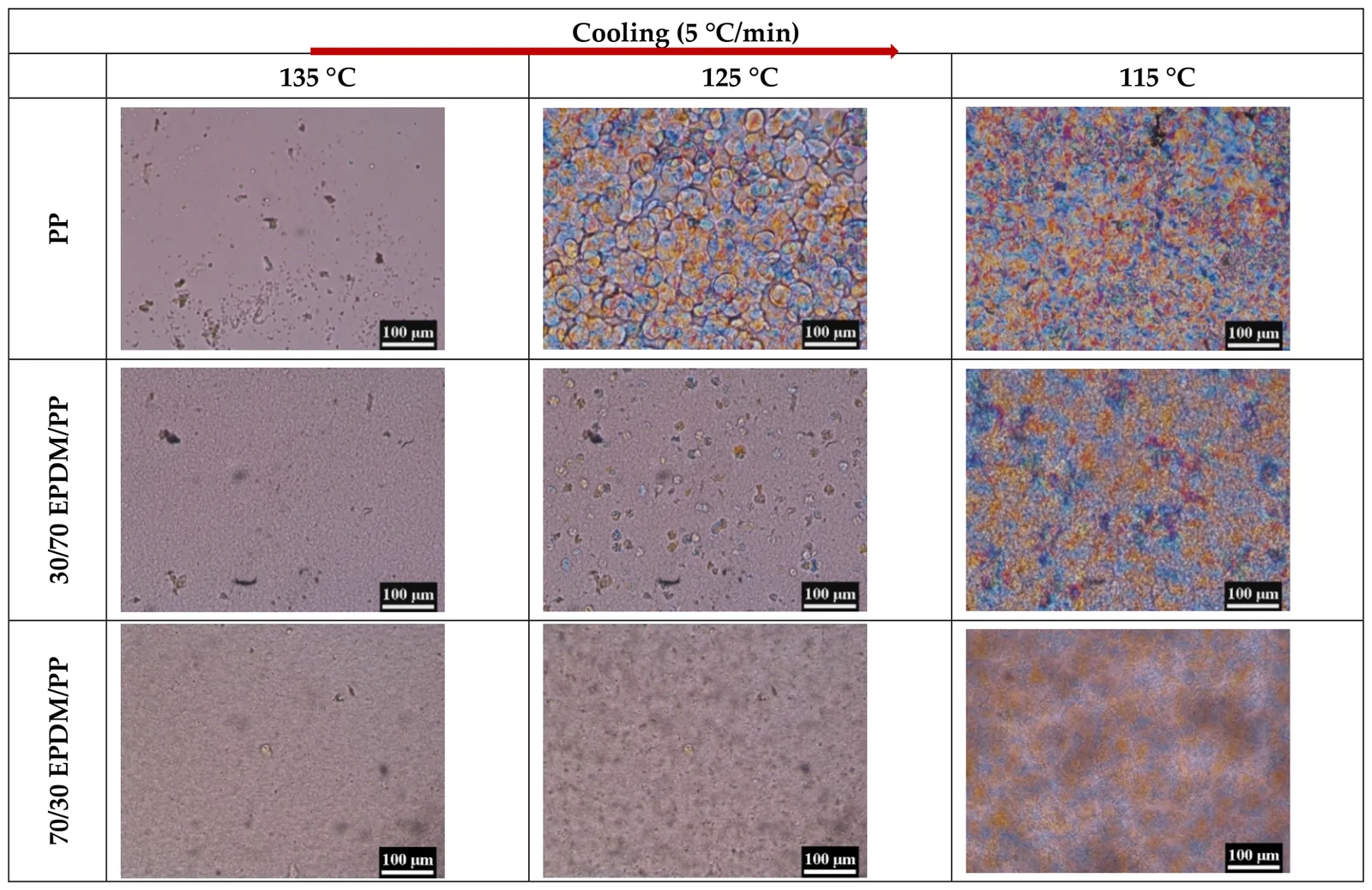
POM micrographs obtained at different cooling temperatures for PP, 30/70 EPDM/PP, and 70/30 EPDM/PP samples.

**Figure 24:**
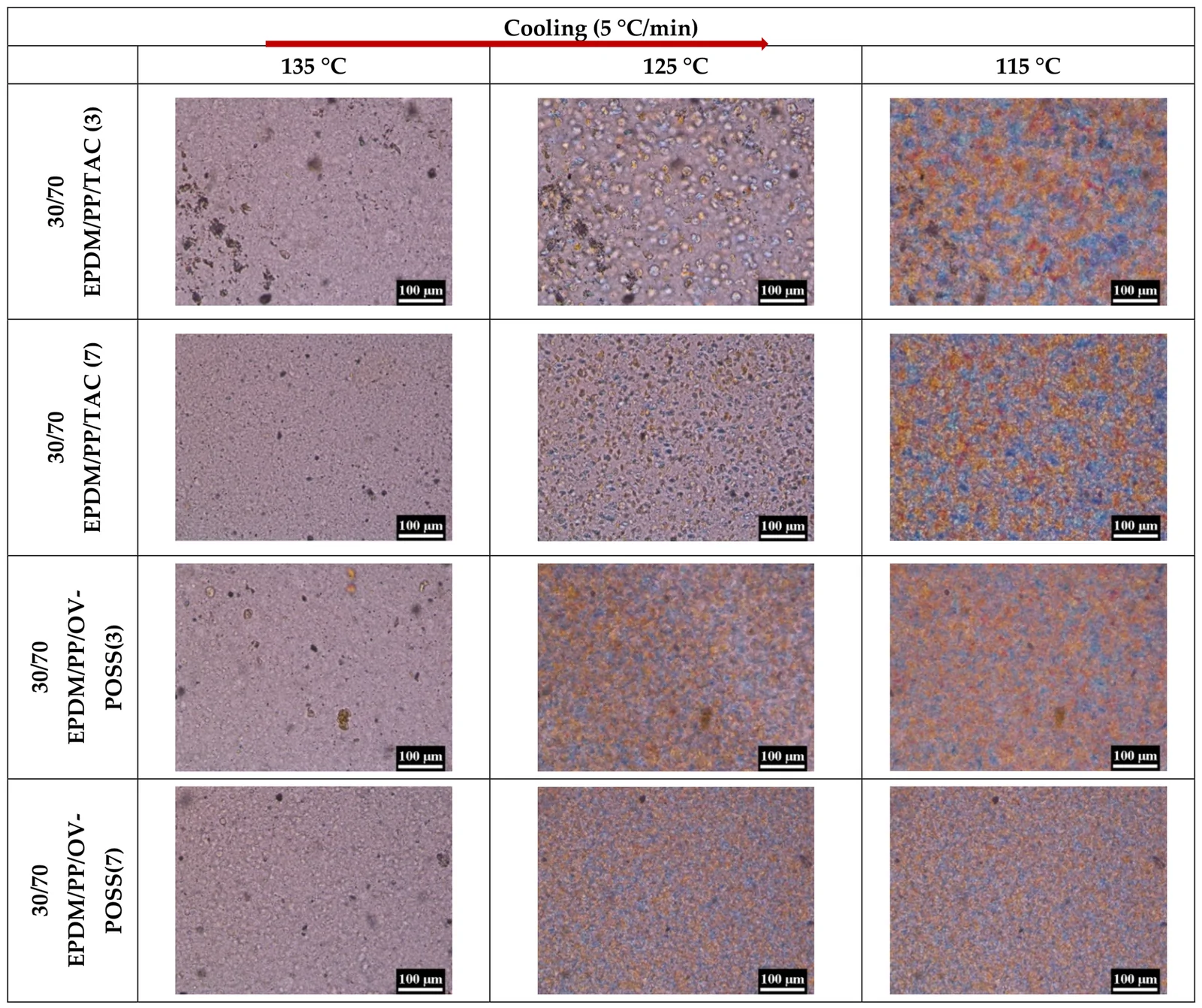
POM micrographs obtained at different cooling temperatures for 30/70 EPDM/PP/coagent TPVs.

**Figure 25:**
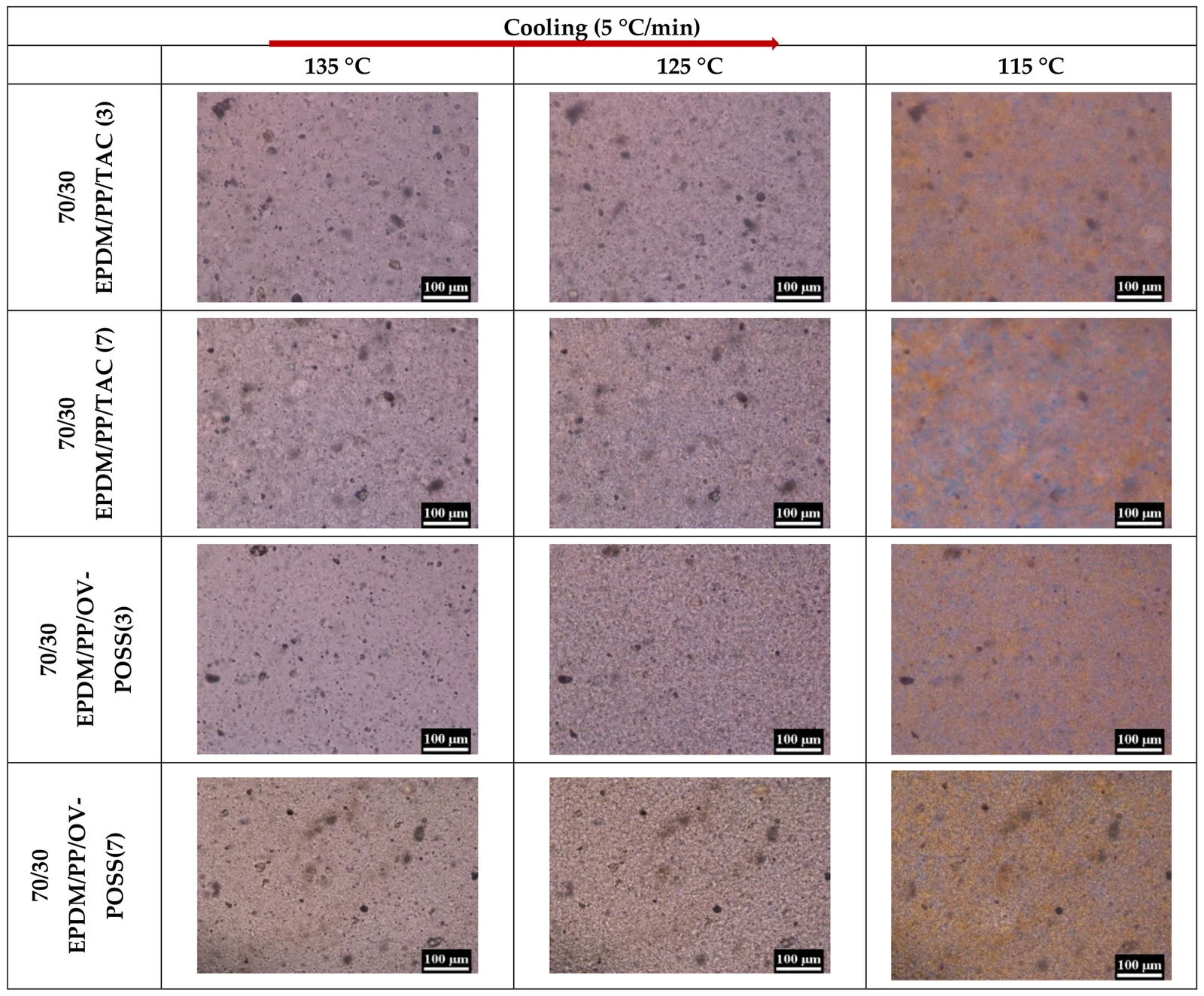
POM micrographs obtained at different cooling temperatures for 70/30 EPDM/PP/coagent TPVs.

Figure 23, Figure 24 and Figure 25 present the crystal morphologies obtained from the POM analyses of neat PP, 30/70 EPDM/PP and 70/30 EPDM/PP blends, and the corresponding TPVs prepared at coagent-to-DCP mass ratios of 3 and 7, during cooling. As can be seen from Figure 23, no crystalline structures were observed in neat PP at 135 °C. However, upon cooling to 125 °C, crystalline entities became visible, and crystallization was nearly complete at 115 °C. The resulting crystals predominantly exhibited a spherical morphology. The diameters of the observed spherulites were approximately 30–35 μm.

As shown in Figure 24, the 30/70 EPDM/PP TPVs containing TAC and OV-POSS exhibited earlier crystal formation and visually finer spherulitic structures than the corresponding blend. In particular, crystalline structures were already visible at 135 °C in the OV-POSS-containing formulations, including at the lower coagent-to-DCP mass ratio. Smaller spherulites and a greater number of visible crystalline entities per unit area were qualitatively observed. These observations are consistent with facilitated heterogeneous nucleation in the OV-POSS-containing formulations and with the higher crystallization onset temperatures observed by DSC.

As shown in Figure 25, the 70/30 EPDM/PP TPVs containing TAC and OV-POSS exhibited earlier crystal formation and visually finer spherulitic structures than the corresponding blend. In particular, OV-POSS-containing formulations showed a greater number of visible crystalline entities per unit area at 125 °C, including at the lower coagent-to-DCP mass ratio. These qualitative observations are consistent with facilitated heterogeneous nucleation, particularly in the presence of OV-POSS, and with the higher crystallization onset temperatures observed by DSC. The smaller spherulites observed in the coagent-containing formulations may reflect a higher number of nucleation sites and earlier impingement of neighboring crystals. Therefore, the POM observations support differences in crystalline morphology but do not, by themselves, establish restricted PP-chain mobility or a lower crystal-growth rate.

The POM micrographs were analyzed quantitatively to determine the spherulite size. The images were spatially calibrated using the 100 µm scale bar. Spherulites were segmented along their boundaries, and the equivalent circular diameter was calculated for each spherulite.

For neat PP, the average spherulite diameter at 125 °C was 38.7 ± 11.2 µm. In the 30/70 formulations, the crystalline entities at 125 °C were still growing as discrete spherulites. Their average diameters were 13.7 ± 3.1 µm for the EPDM/PP blend, 16.6 ± 7.1 µm for TAC(3), and 12.5 ± 3.4 µm for TAC(7). For the OV-POSS-containing TPVs and the 70/30 formulations, the crystalline texture was very fine and individual spherulite boundaries could not be reliably resolved. This suggests the formation of finer crystalline structures, although reliable quantitative size determination was not possible for these formulations.

These quantitative results confirm the reduction in spherulite size upon coagent incorporation, particularly with OV-POSS, and are consistent with the higher crystallization onset temperatures (Table 2 and Table 3) and the lower Dobreva-Gutzow nucleation activity values (Table 9 and Table 10).

### 3.3. X-Ray Diffraction (XRD) Results

**Figure 26:**
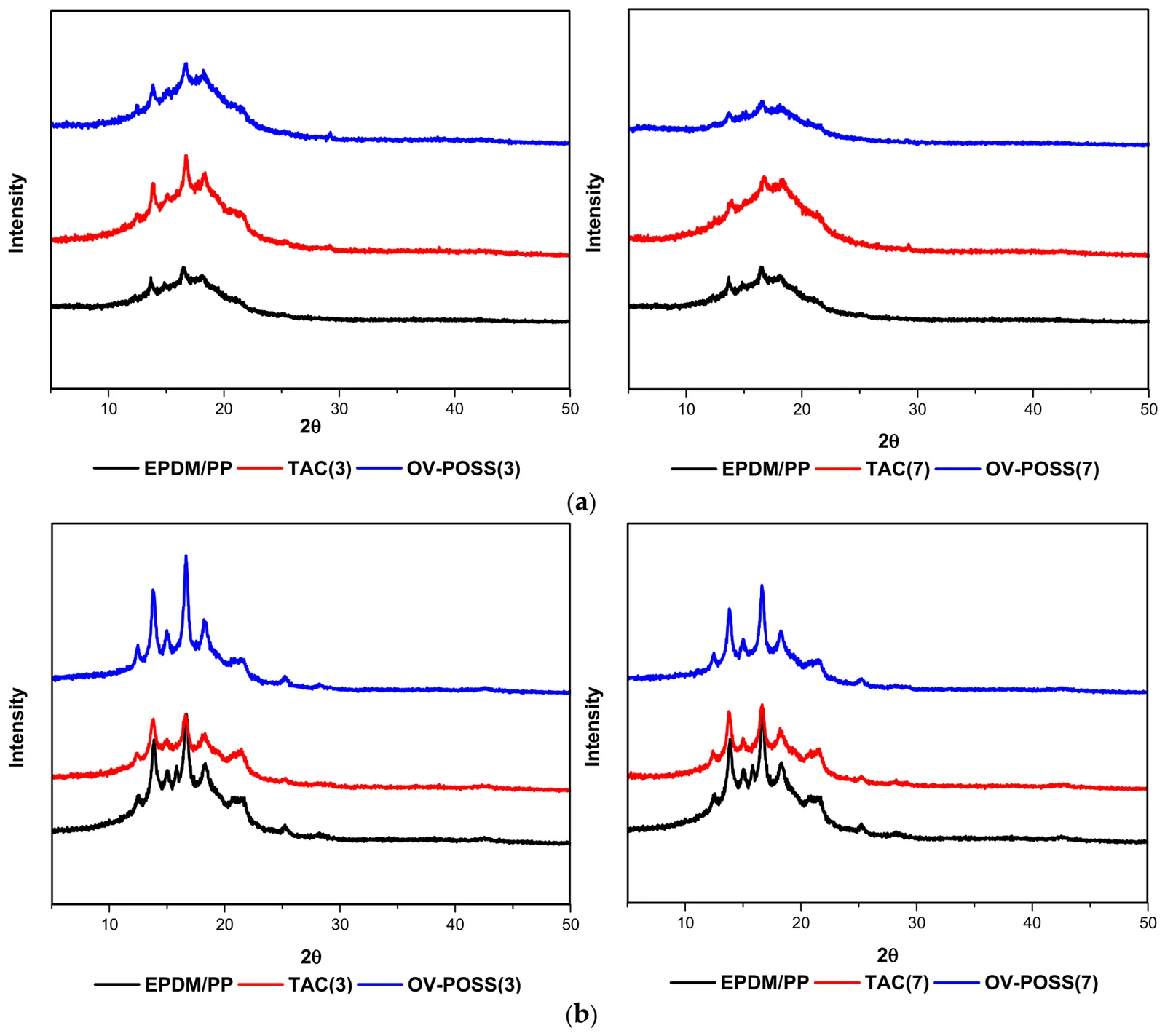
XRD patterns of (**a**) 70/30 and (**b**) 30/70 EPDM/PP blends, and EPDM/PP/coagent(3) and EPDM/PP/coagent(7) TPVs.

To determine the crystal structures of the EPDM/PP blends and EPDM/PP/coagent(X) TPV components, X-ray diffraction (XRD) measurements were carried out in the 2θ range of 5–50° (Figure 26). As described previously, the characteristic diffraction peaks observed at 2θ = 13.9° [110], 16.7° [040], 18.3° [130], 20.8° [131], 21.6° [111], and 25.2° [060] for the 30/70 EPDM/PP blend are associated with the monoclinic α-crystalline structure of the PP component [43,44,45]. The diffraction peak observed at 2θ = 15.8° corresponds to the β-crystalline form of PP and is assigned to the [300] crystallographic plane [43].

The XRD patterns revealed distinct differences depending on the EPDM/PP composition. The PP-rich 30/70 formulations exhibited more pronounced crystalline reflections, whereas the EPDM-rich 70/30 formulations showed a greater relative contribution from the amorphous phase and less distinct crystalline peaks. In particular, several higher-angle α-crystalline reflections and the characteristic β-crystalline reflection were not clearly resolved in the 70/30 formulations. Nevertheless, the remaining α-crystalline reflections confirmed the presence of crystalline PP structures.

To quantitatively evaluate the crystalline structures, the overall degree of crystallinity (X_c,XRD_) was calculated from the integrated areas of the crystalline diffraction peaks (A_c_) and amorphous scattering contribution (A_a_), using the following Equation (18):

(18) $$X_{c,XRD}\left(\%\right)=\frac{A_{c}}{A_{c}\times A_{a}}\times 100$$

**Figure 27:**
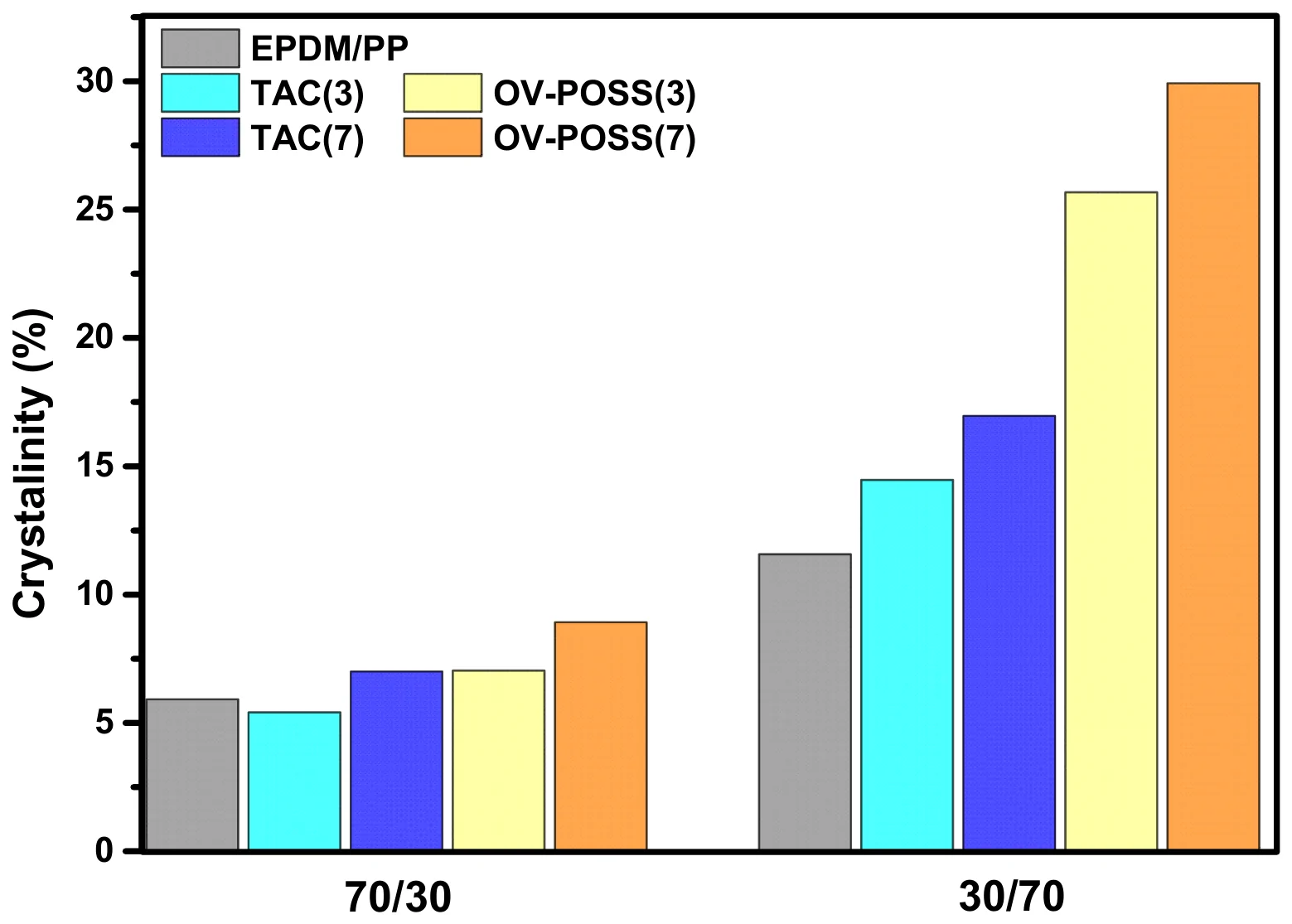
XRD-derived degree of crystallinity of 70/30 and 30/70 EPDM/PP blends and the corresponding TAC and OV-POSS-containing TPVs.

The integrated crystalline peak areas were also used to distinguish the contributions of the α- and β-crystalline forms. The calculated overall crystallinity values are presented in Figure 27.

As shown in Figure 27, the overall crystallinity was strongly influenced by the EPDM/PP composition and coagent type. In the PP-rich 30/70 system, both TAC and OV-POSS increased crystallinity, although the effect was substantially more pronounced for OV-POSS. At the higher OV-POSS loading, the crystallinity increased from 11.52% for the reference blend to 29.91%. Analysis of the individual crystalline contributions revealed that this increase was mainly associated with the α-crystalline form, which accounted for 27.96%, while the β-crystalline contribution remained limited to 1.95%. These findings indicate that OV-POSS incorporation enhanced the development of crystalline PP structures without substantially changing the predominance of the α-crystalline form.

A similar but less pronounced trend was observed for the EPDM-rich 70/30 formulations. The overall crystallinity remained relatively low due to the reduced PP content, while OV-POSS incorporation increased the crystalline fraction, particularly at the higher coagent loading. In contrast, TAC produced only minor changes. The crystalline reflections in these formulations were predominantly associated with the α-form, with no clearly resolvable β-crystalline contribution.

Overall, the XRD results demonstrated that OV-POSS incorporation increased the crystalline fraction in both EPDM/PP compositions, predominantly through an enhanced α-crystalline contribution. These findings are qualitatively consistent with the DSC results discussed previously. The increase in crystallinity, together with the higher crystallization onset temperatures and POM observations, suggests that OV-POSS promotes heterogeneous nucleation and facilitates the development of crystalline PP structures. However, the higher crosslink densities reported previously for OV-POSS-containing TPVs [13] may also restrict polymer chain mobility and subsequent crystal growth. Thus, enhanced nucleation may coexist with constraints on crystal development, without necessarily reducing the final degree of crystallinity. This suggests that the crystallization behavior of OV-POSS-containing TPVs is governed by a balance between nucleation promotion and mobility-related restrictions, depending on blend composition and coagent loading.

## 4. Conclusions

The non-isothermal crystallization of EPDM/PP blends and the corresponding TPVs dynamically vulcanized with TAC or OV-POSS was investigated using DSC, kinetic modeling, POM, and XRD.

Both coagents shifted the onset of PP crystallization to higher temperatures at all cooling rates. The shift was generally larger for OV-POSS. In the 30/70 formulations at 10 °C/min, T_c,o_ increased from 113.6 °C for the blend to 124.2 °C for OV-POSS(7). In the 70/30 formulations, OV-POSS increased T_c,o_ by approximately 9–11 °C. Dobreva-Gutzow Φ values below unity relative to the corresponding unvulcanized blends (0.57–0.87) are consistent with facilitated heterogeneous nucleation. The experimental *t_1/2_* and Avrami-derived *t_0.5_* both decreased with increasing cooling rate. However, the effect of the coagents on the time needed to reach 50% relative crystallinity was not uniform. It depended on blend composition, coagent loading, and cooling rate. Higher OV-POSS loading did not consistently shorten *t_0.5_*, and in the EPDM-rich 70/30 system both OV-POSS formulations showed longer half-times than the unvulcanized blend. The combined Avrami-Ozawa model gave good linear correlations for all formulations. The Ozawa model could not be applied reliably because the crystallization intervals at different cooling rates overlapped too little. OV-POSS increased the PP-normalized crystallinity determined by DSC in both compositions, from 35.1% to 41.9% (30/70) and from 32.7% to 40.5% (70/30) at the higher loading. XRD likewise showed a larger crystalline fraction, mainly in the α-form, with the most pronounced effect in the PP-rich formulations. POM showed earlier appearance of crystalline entities and finer spherulitic textures in the coagent-containing TPVs, particularly with OV-POSS.

Taken together, these results show that a higher crystallization onset temperature and higher final crystallinity do not necessarily come with faster overall crystallization. A possible interpretation is that OV-POSS incorporation and the finer, more homogeneously dispersed crosslinked EPDM domains may provide additional sites for heterogeneous nucleation. Meanwhile, the higher network density previously reported for the OV-POSS-containing TPVs and earlier impingement of densely nucleated crystals may influence subsequent crystal development. From a practical standpoint, the higher crystallization temperatures suggest earlier solidification during processing, while the effect on overall cycle time depends on blend composition and coagent loading.

## Figures and Tables

**Table 1:** Composition and designation of the investigated samples.

EPDM * (phr)	PP (phr)	DCP (phr)	X (Coagent/DCP Ratio)	Antioxidants (phr)	Abbreviation
140	30	0	-	0	70/30 EPDM/PP
60	70				30/70 EPDM/PP
140	30	1.75	3 or 7	0.3	70/30 EPDM/PP/Coagent(X)
60	70	0.75		0.7	30/70 EPDM/PP/Coagent(X)

* EPDM includes 50 wt.% paraffin oil (All phr values are expressed per 100 parts by weight of the combined dry EPDM and PP components).

**Table 2:** Characteristic crystallization parameters of neat PP, 30/70 EPDM/PP, and 30/70 EPDM/PP/Coagent TPVs obtained at different cooling rates.

	Cooling Rate (β, °C/min)	T_c,o_ (°C)	T_c,p_ (°C)	T_c,e_ (°C)	ΔT (°C)	Crystallization Half Time (t*_1/2_*, min)
**PP**	5	119.7	106.0	96.5	23.2	2.6
	10	116.1	100.9	86.8	29.3	1.5
	20	104.1	90.4	65.7	38.4	0.7
	30	100.0	82.1	53.1	46.9	0.6
**EPDM/PP**	5	117.3	108.1	96.9	20.4	1.9
	10	113.6	101.4	85.6	28.0	1.2
	20	101.4	87.2	58.7	42.7	0.7
	30	95.4	81.9	52.6	42.8	0.5
**EPDM/PP/TAC(3)**	5	117.9	110.2	101.9	16.0	1.5
	10	113.8	104.1	89.9	23.9	1.0
	20	104.5	95.3	75.1	29.4	0.5
	30	98.6	87.7	57.0	41.6	0.4
**EPDM/PP/TAC(7)**	5	122.2	112.9	101.4	20.8	1.9
	10	116.6	106.8	89.6	27.0	1.1
	20	108.8	98.1	72.4	36.4	0.6
	30	101.3	90.4	66.2	35.1	0.4
**EPDM/PP/OV-POSS(3)**	5	127.5	117.8	104.4	23.1	2.0
	10	121.4	111.6	96.8	24.6	1.0
	20	113.2	101.5	75.5	37.7	0.7
	30	109.1	96.0	71.4	37.7	0.5
**EPDM/PP/OV-POSS(7)**	5	128.7	118.8	103.7	25.0	2.0
	10	124.2	111.9	95.8	28.4	1.3
	20	115.3	103.7	81.4	33.9	0.6
	30	108.1	94.4	64.7	43.4	0.5

**Table 3:** Characteristic crystallization parameters of neat PP, 70/30 EPDM/PP, and 70/30 EPDM/PP/Coagent TPVs obtained at different cooling rates.

	Cooling Rate (β, °C/min)	T_c,o_ (°C)	T_c,p_ (°C)	T_c,e_ (°C)	ΔT (°C)	Crystallization Half Time (t*_1/2_*, min)
**PP**	5	119.7	106.0	96.5	23.2	2.6
	10	116.1	100.9	86.8	29.3	1.5
	20	104.1	90.4	65.7	38.4	0.7
	30	100.0	82.1	53.1	46.9	0.6
**EPDM/PP**	5	111.0	104.0	97.0	14.0	1.3
	10	106.5	96.8	84.8	21.7	0.9
	20	97.0	88.5	71.0	26.0	0.4
	30	89.2	79.5	57.2	32.0	0.3
**EPDM/PP/TAC(3)**	5	115.0	107.6	99.5	15.5	1.4
	10	111.6	101.4	90.0	21.6	1.0
	20	99.3	89.2	66.2	33.1	0.5
	30	93.4	82.2	59.4	34.0	0.4
**EPDM/PP/TAC(7)**	5	116.8	108.1	100.0	16.8	1.7
	10	113.8	102.4	88.5	25.3	1.1
	20	102.0	90.0	65.4	36.6	0.6
	30	93.6	81.7	56.0	37.6	0.4
**EPDM/PP/OV-POSS(3)**	5	123.0	112.1	104.1	18.9	2.1
	10	117.0	104.5	89.3	27.7	1.2
	20	108.1	96.0	74.1	34.0	0.6
	30	98.3	85.6	61.9	36.4	0.4
**EPDM/PP/OV-POSS(7)**	5	121.2	111.0	102.7	18.5	2.0
	10	115.3	104.3	91.3	24.0	1.1
	20	108.6	96.2	76.8	31.8	0.8
	30	100.1	86.8	58.6	41.5	0.7

**Table 4:** Crystallization enthalpy (ΔH_c_) determined during cooling at 10 °C/min, and melting enthalpy (ΔH_m_) and PP-normalized degree of crystallinity (X_c,DSC_) determined from the subsequent second heating scans of EPDM/PP blends and TPVs.

	w_PP_ *	ΔH_c_ (J/g)	ΔH_m_ (J/g)	X_c,DSC_ (%)
**30/70 EPDM/PP**	0.5385	42.4	39.5	35.1
**30/70 EPDM/PP/TAC(3)**	0.5237	42.0	39.2	35.8
**30/70 EPDM/PP/TAC(7)**	0.5123	46.3	42.0	39.2
**30/70 EPDM/PP/OV-POSS(3)**	0.5237	48.5	40.4	36.9
**30/70 EPDM/PP/OV-POSS(7)**	0.5123	50.9	44.9	41.9
**70/30 EPDM/PP**	0.1765	13.8	12.1	32.7
**70/30 EPDM/PP/TAC(3)**	0.1693	13.8	12.0	34.0
**70/30 EPDM/PP/TAC(7)**	0.1629	13.7	12.0	35.2
**70/30 EPDM/PP/OV-POSS(3)**	0.1693	15.0	13.2	37.4
**70/30 EPDM/PP/OV-POSS(7)**	0.1629	15.5	13.8	40.5

* w_PP_ represents the mass fraction of PP in the total formulation, including the paraffinic oil contained in the commercial EPDM and all other formulation components. The DSC-derived crystallinity values were normalized to the PP content.

**Table 5:** Avrami kinetic parameters obtained at different cooling rates for PP, 30/70 EPDM/PP, 30/70 EPDM/PP/TAC, and EPDM/PP/OV-POSS TPVs.

	β (°C/min)	n	Z	Z_c_	*t_0.5_* (min)	R^2^
**PP**	5	4.92	5.83 × 10^−3^	3.57 × 10^−1^	2.64	0.997
	10	5.11	7.44 × 10^−2^	7.71 × 10^−1^	1.55	1.000
	20	3.45	1.79	1.03	0.76	0.994
	30	3.55	3.01	1.04	0.66	0.999
**30/70 EPDM/PP**	5	4.10	5.40 × 10^−2^	5.58 × 10^−1^	1.86	1.000
	10	4.59	2.41 × 10^−1^	8.67 × 10^−1^	1.26	0.999
	20	3.17	1.64	1.03	0.76	0.999
	30	2.82	4.92	1.05	0.50	0.999
**30/70 EPDM/PP/TAC(3)**	5	4.01	1.21 × 10^−1^	6.56 × 10^−1^	1.54	1.000
	10	4.41	7.23 × 10^−1^	9.68 × 10^−1^	0.99	0.999
	20	3.27	5.53	1.09	0.53	0.998
	30	3.00	8.64	1.07	0.43	0.997
**30/70 EPDM/PP/TAC(7)**	5	4.83	3.25 × 10^−2^	5.04 × 10^−1^	1.88	1.000
	10	3.92	5.56 × 10^−1^	9.43 × 10^−1^	1.06	0.999
	20	3.11	3.26	1.06	0.61	0.999
	30	2.15	1.02 × 10^1^	1.08	0.29	0.997
**30/70 EPDM/PP/OV-POSS(3)**	5	4.13	3.97 × 10^−2^	5.25 × 10^−1^	2.00	0.999
	10	3.95	6.04 × 10^−1^	9.51 × 10^−1^	1.04	1.000
	20	2.73	2.04	1.04	0.67	0.999
	30	2.96	5.08	1.06	0.51	0.998
**30/70 EPDM/PP/OV-POSS(7)**	5	4.31	3.31 × 10^−2^	5.06 × 10^−1^	2.03	1.000
	10	4.41	2.15 × 10^−1^	8.58 × 10^−1^	1.30	0.999
	20	3.57	3.35	1.06	0.64	0.996
	30	2.79	4.36	1.05	0.52	0.999

**Table 6:** Avrami kinetic parameters obtained at different cooling rates for PP, 70/30 EPDM/PP, 70/30 EPDM/PP/TAC, and EPDM/PP/OV-POSS TPVs.

	β (°C/min)	n	Z	Z_c_	*t_0.5_* (min)	R^2^
**PP**	5	4.92	5.83 × 10^−3^	3.57 × 10^−1^	2.64	0.997
	10	5.11	7.44 × 10^−2^	7.71 × 10^−1^	1.55	1.000
	20	3.45	1.79	1.03	0.76	0.994
	30	3.55	3.01	1.04	0.66	0.999
**70/30 EPDM/PP**	5	4.00	2.01 × 10^−1^	7.26 × 10^−1^	1.36	1.000
	10	4.78	8.37 × 10^−1^	9.82 × 10^−1^	0.96	1.000
	20	3.45	9.71	1.12	0.47	0.997
	30	2.76	1.16 × 10^1^	1.09	0.36	0.997
**70/30 EPDM/PP/TAC(3)**	5	3.98	1.58 × 10^−1^	6.91 × 10^−1^	1.45	0.999
	10	4.43	1.52	1.04	0.84	0.999
	20	2.89	3.71	1.07	0.56	0.997
	30	2.97	9.58	1.08	0.41	0.996
**70/30 EPDM/PP/TAC(7)**	5	4.66	6.14 × 10^−2^	5.72 × 10^−1^	1.68	1.000
	10	4.67	3.85 × 10^−1^	9.09 × 10^−1^	1.13	0.999
	20	3.34	2.71	1.05	0.66	0.995
	30	2.89	6.98	1.07	0.45	0.996
**70/30 EPDM/PP/OV-POSS(3)**	5	4.64	2.03 × 10^−2^	4.59 × 10^−1^	2.14	1.000
	10	4.73	2.30 × 10^−1^	8.63 × 10^−1^	1.26	1.000
	20	3.71	3.40	1.06	0.65	0.997
	30	3.00	7.31	1.07	0.46	0.997
**70/30 EPDM/PP/OV-POSS(7)**	5	4.42	3.53 × 10^−2^	5.12 × 10^−1^	1.96	1.000
	10	4.05	4.32 × 10^−1^	9.19 × 10^−1^	1.12	0.999
	20	5.01	1.63	1.02	0.84	0.995
	30	3.46	2.74	1.03	0.67	0.993

**Table 7:** Combined Avrami-Ozawa kinetic parameters for PP, 30/70 EPDM/PP, and 30/70 EPDM/PP/Coagent TPVs.

	X(t) (%)	α	F(T)	R2
**PP**	10	1.16	9.28	0.97
	30	1.16	13.54	0.98
	50	1.19	16.19	0.98
	70	1.27	19.55	0.99
	90	1.37	25.37	0.99
**30/70 EPDM/PP**	10	1.15	7.13	0.96
	30	1.29	9.98	0.98
	50	1.36	12.58	0.99
	70	1.43	15.67	0.99
	90	1.60	22.72	0.99
**30/70 EPDM/PP/TAC(3)**	10	1.24	5.24	0.98
	30	1.29	7.44	0.99
	50	1.34	9.14	0.99
	70	1.41	11.28	1.00
	90	1.54	15.50	0.99
**30/70 EPDM/PP/TAC(7)**	10	1.09	6.56	1.00
	30	1.15	8.85	1.00
	50	1.20	10.66	1.00
	70	1.26	12.93	1.00
	90	1.37	17.19	1.00
**30/70 EPDM/PP/OV-POSS(3)**	10	1.18	6.42	0.98
	30	1.24	9.19	0.99
	50	1.31	11.62	0.99
	70	1.38	14.64	0.98
	90	1.48	20.57	0.97
**30/70 EPDM/PP/OV-POSS(7)**	10	1.15	7.46	0.98
	30	1.19	10.24	0.99
	50	1.26	12.67	0.98
	70	1.32	15.57	0.98
	90	1.43	21.38	0.98

**Table 8:** Combined Avrami-Ozawa kinetic parameters for PP, 70/30 EPDM/PP, and 70/30 EPDM/PP/Coagent TPVs.

	X(t) (%)	α	F(T)	R2
**PP**	10	1.16	9.28	0.97
	30	1.16	13.54	0.98
	50	1.19	16.19	0.98
	70	1.27	19.55	0.99
	90	1.37	25.37	0.99
**70/30 EPDM/PP**	10	1.14	4.87	0.95
	30	1.19	6.72	0.97
	50	1.24	8.19	0.98
	70	1.32	9.77	0.98
	90	1.50	13.08	0.99
**70/30 EPDM/PP/TAC(3)**	10	1.23	5.05	0.96
	30	1.30	7.31	0.98
	50	1.34	9.01	0.99
	70	1.46	11.34	0.99
	90	1.65	16.29	0.99
**70/30 EPDM/PP/TAC(7)**	10	1.18	6.36	0.97
	30	1.29	8.82	0.99
	50	1.34	10.82	0.99
	70	1.45	13.36	0.99
	90	1.66	19.29	0.99
**70/30 EPDM/PP/OV-POSS(3)**	10	1.03	7.69	0.99
	30	1.09	10.32	1.00
	50	1.13	12.34	1.00
	70	1.19	14.59	1.00
	90	1.33	19.61	1.00
**70/30 EPDM/PP/OV-POSS(7)**	10	1.77	6.88	0.96
	30	1.65	11.16	0.97
	50	1.67	14.30	0.98
	70	1.71	17.99	0.98
	90	1.90	27.85	0.97

**Table 9:** Dobreva-Gutzow parameters and activation energy values obtained from different mathematical models for PP, 30/70 EPDM/PP, and 30/70 EPDM/PP/Coagent TPVs.

	B	B*	ϕ = B*/B	Activation Energy (kJ/mol) Kissinger	Activation Energy (kJ/mol) Takhor
**PP**	12,039	-	-	−86.90	−80.79
**30/70 EPDM/PP**	-	8556 *	0.71	−78.33	−72.22
	8556 **	-	-		
**30/70 EPDM/PP/TAC(3)**	-	7472	0.87	−96.51	−90.33
**30/70 EPDM/PP/TAC(7)**	-	6581	0.77	−98.10	−91.87
**30/70 EPDM/PP/OV-POSS(3)**	-	5137	0.60	−102.2	−95.89
**30/70 EPDM/PP/OV-POSS(7)**	-	4881	0.57	−94.41	−88.10

* The EPDM/PP blend value is B* when compared with neat PP. ** The same blend value is the reference B when compared with coagent-containing TPVs.

**Table 10:** Dobreva-Gutzow parameters and activation energy values obtained from different mathematical models for PP, 70/30 EPDM/PP, and 70/30 EPDM/PP/Coagent TPVs.

	B	B*	ϕ = B*/B	Activation Energy (kJ/mol) Kissinger	Activation Energy (kJ/mol) Takhor
**PP**	12,039	-	-	−86.90	−80.79
**70/30 EPDM/PP**	-	8143 *	0.68	−87.12	−81.06
	8143 **	-	-		
**70/30 EPDM/PP/TAC(3)**	-	6444	0.79	−82.19	−76.07
**70/30 EPDM/PP/TAC(7)**	-	6216	0.76	−78.93	−72.82
**70/30 EPDM/PP/OV-POSS(3)**	-	5816	0.71	−84.10	−77.93
**70/30 EPDM/PP/OV-POSS(7)**	-	6546	0.80	−91.02	−84.84

* The EPDM/PP blend value is B* when compared with neat PP. ** The same blend value is the reference B when compared with coagent-containing TPVs.

## Data Availability

The data presented in this study are available on request from the corresponding author.
